# Implications of ethylene biosynthesis and signaling in soybean drought stress tolerance

**DOI:** 10.1186/s12870-015-0597-z

**Published:** 2015-09-03

**Authors:** Fabricio Barbosa Monteiro Arraes, Magda Aparecida Beneventi, Maria Eugenia Lisei de Sa, Joaquin Felipe Roca Paixao, Erika Valeria Saliba Albuquerque, Silvana Regina Rockenbach Marin, Eduardo Purgatto, Alexandre Lima Nepomuceno, Maria Fatima Grossi-de-Sa

**Affiliations:** Federal University of Rio Grande do Sul, Campus do Vale, Av. Bento Gonçalves 9500, Postal Code 15005 CEP 91501–970, Porto Alegre, RS Brazil; Embrapa Genetic Resources and Biotechnology, PqEB, Av. W5-Norte, Postal Code 02372 CEP 70770–910, Brasilia, DF Brazil; Brasilia University – Biology Institute, Brasilia, DF Brazil; Agricultural Research Company of Minas Gerais State, Rua Afonso Rato 1301, Postal Code 311 CEP 38001–970, Uberaba, MG Brazil; Embrapa Soybean, Rodovia Carlos João Strass, SN, Acesso Orlando Amaral, Distrito de Warta, Postal Code 231 CEP 86001–970, Londrina, PR Brazil; Food Chemistry and Biochemistry Laboratory, Sao Paulo University, Av. Lineu Prestes 580, Bloco 14, Cidade Universitaria, CEP 05508–000, Sao Paulo, SP Brazil; Catholic University of Brasilia, SGAN 916, Modulo B, Av. W5, Asa Norte, CEP 70790–160, Brasilia, DF Brazil

## Abstract

**Background:**

Ethylene is a phytohormone known for inducing a triple response in seedlings, leaf abscission and other responses to various stresses. Several studies in model plants have evaluated the importance of this hormone in crosstalk signaling with different metabolic pathways, in addition to responses to biotic stresses. However, the mechanism of action in plants of agricultural interest, such as soybean, and its participation in abiotic stresses remain unclear.

**Results:**

The studies presented in this work allowed for the identification of 176 soybean genes described elsewhere for ethylene biosynthesis (108 genes) and signal transduction (68 genes). A model to predict these routes in soybean was proposed, and it had great representability compared to those described for *Arabidopsis thaliana* and *Oryza sativa*. Furthermore, analysis of putative gene promoters from soybean gene orthologs permitted the identification of 29 families of *cis*-acting elements. These elements are essential for ethylene-mediated regulation and its possible crosstalk with other signaling pathways mediated by other plant hormones.

From genes that are differentially expressed in the transcriptome database, we analyzed the relative expression of some selected genes in resistant and tolerant soybean plants subjected to water deficit. The differential expression of a set of five soybean ethylene-related genes (*MAT*, *ACS*, *ACO*, *ETR* and *CTR*) was validated with RT-qPCR experiments, which confirmed variations in the expression of these soybean target genes, as identified in the transcriptome database. In particular, two families of ethylene biosynthesis genes (*ACS* and *ACO*) were upregulated under these experimental conditions, whereas *CTR* (involved in ethylene signal transduction) was downregulated. In the same samples, high levels of ethylene production were detected and were directly correlated with the free fraction levels of ethylene’s precursor. Thus, the combination of these data indicated the involvement of ethylene biosynthesis and signaling in soybean responses to water stress.

**Conclusions:**

The *in silico* analysis, combined with the quantification of ethylene production (and its precursor) and RT-qPCR experiments, allowed for a better understanding of the importance of ethylene at a molecular level in this crop as well as its role in the response to abiotic stresses. In summary, all of the data presented here suggested that soybean responses to water stress could be regulated by a crosstalk network among different signaling pathways, which might involve various phytohormones, such as auxins, ABA and jasmonic acid. The integration of *in silico* and physiological data could also contribute to the application of biotechnological strategies to the development of improved cultivars with regard to different stresses, such as the isolation of stress-specific plant promoters.

**Electronic supplementary material:**

The online version of this article (doi:10.1186/s12870-015-0597-z) contains supplementary material, which is available to authorized users.

## Background

Phytohormones are organic compounds that exist naturally in plants and that even in low concentrations, orchestrate a broad range of physiological processes, including growth and development, as well as responses to abiotic and biotic stresses [1]. These hormones overlap signal transduction pathways or gene expression profiles by rapid induction or by preventing the degradation of transcriptional regulators [2–5].

Among all of the described phytohormones, ethylene, a naturally occurring triple response growth regulator (shoot elongation, stem thickening and horizontal growth habit) in seedlings, has been studied since ancient times [6]. Ethylene is also involved in leaf abscission, fruit ripening and senescence [6, 7] as well as seed germination, growth of adventitious roots under flooding conditions, epinasty stimulation, inhibition of shoot growth and stomatal closing and flowering [8, 9]. Moreover, it is involved in a wide variety of stresses, including wounding, pathogen attack, flooding, drought, hypoxia, and temperature shifts [10, 11].

Ethylene biosynthesis is derived from the amino acid methionine provided by the Yang cycle [12], in which the precursor S-adenosylmethionine (AdoMet or SAM) is synthesized from ATP and methionine by S-adenosylmethionine synthetase (SAMS; EC 2.5.1.6) [13]. AdoMet is then converted into 1-aminocyclopropane-1-carboxylic acid (ACC) and 5-methylthioadenosine (MTA) by the enzyme 1-aminocyclopropane-1-carboxylase synthase (ACS, EC 4.4.1.14) [13]. MTA is recycled through a series of Yang cycle reactions back to methionine [14].

Active ACSs are encoded by eight genes in *Arabidopsis thaliana*, and at least one encodes a catalytically inactive ACS (AtACS1) [15–17]. Based on the sequence present in its C-terminal region, these proteins can be divided into three main groups: *type I* proteins, which are the targets for phosphorylation by mitogen-activated protein kinase 3 and/or 6 (AtMPK3-6; EC 2.7.11.24) [18] as well as by calcium-dependent protein kinase (AtCDPK2; CDPK or CPK; EC 2.7.11.1); *type II* proteins, which show phosphorylation sites for only CPK [19]; and *type III* proteins, which have the C-terminal portion greatly reduced and do not present phosphorylation sites for either kinase. Furthermore, the ACSs can be regulated by putative endogenous signal receptors (*i.e.*, phytohormones) and/or intracellular accumulation of secondary metabolites, such as calcium. In the absence of an endogen signal, type II ACSs are degraded by 26S proteasome. This degradation is mediated by ETO proteins (ethylene overproducer*)* and EOL (ETO-like), which are members of specific plant proteins with E3 ubiquitin ligase domain [20]. This process activates kinase protein signaling, which culminates in the stabilization of type II ACSs. Furthermore, MPK3-6 kinases are able to phosphorylate the C-terminal of type I ACSs, which preserve and stabilize their degradation via the 26S proteasome pathway, thereby increasing the production of ethylene and inducing other ethylene-dependent signaling pathways [21].

The enzyme directly responsible for the ethylene biosynthesis is 1-aminociclopropane-1-acid carboxylic oxidase (ACO or EFE - ethylene forming enzyme; EC 1.14.17.4), which converts ACC into this plant hormone [22].

Several reports have suggested that the ACC metabolite could combine with other organic molecules. Different studies have demonstrated that the ACC N-malonyzation pathway in various plant tissues is involved in the regulation of ethylene production, wherein the conjugate 1-malonyl-ACC (MACC) is formed by 1-aminocyclopropane-1-acid carboxylic acid-N-malonyltransferase, an enzyme that has been purified from plant protein extracts but without reference to its respective gene [23, 24]. In addition to MACC formation through a metabolic route, ACC can also be conjugated in the form of 1-glutamyl-ACC (GACC) in a reaction that is catalyzed by γ-glutamyl transpeptidase (GGT; EC 2.3.2.2) [25].

Another possible ACC metabolic pathway is the reaction catalyzed by the enzyme ACC deaminase (ACD; EC 3.5.99.7), a protein that degrades ACC into oxobutyrate (or OXB; 2-oxobutanoate) and ammonia (NH_3_), thus decreasing the levels of ACC that are available for ethylene production [26, 27]. The *ACD* gene was first identified in *A. thaliana* and *Populus,* and studies of tomato plants have shown that ACD activity varies during fruit ripening and that its peak activity coincides with the reduction in ethylene synthesis [28, 29].

The classic routes of ethylene intracellular signal transduction, initially described in *A. thaliana*, are triggered by the gas interaction with membrane receptors (encoded by *ETR* genes - *ethylene receptor*) and the modulation of CTR1 (constitutive triple response – MKKK; EC 2.7.11.1) activity to regulate the expression of several genes, such as *EIN3/EIL* (*ethylene insensitive 3*; *EIN3-like*). Both receptors and CTR1 function as negative regulators of the signal transduction pathway in the absence of ethylene. The kinase CTR1 phosphorylates the EIN2 (ethylene insensitive 2) C-terminal domain, allowing for the degradation of this protein. ETP1 and ETP2 (EIN2 targeting protein) play important roles in EIN2 proteolysis. These proteins, which have F-box domains, interact with the conserved EIN2 C-terminal domain that was previously phosphorylated by CTR1. Thus, in the absence of ethylene, the phosphorylated EIN2 C-terminal domain is ubiquitinated and then degraded by the 26S proteasome [30]. However, in the presence of ethylene, instead of being phosphorylated, the EIN2 domain is cleaved and transported to the nucleus to stimulate EIN3/EIL activity by repressing EBF (EIN3 binding F-box protein). Thus, EIN3/EINL induce the transcription of target genes, mainly the AP2/ERF transcription factor superfamily [31]. Earlier studies have also suggested an EIN3/EIL activation route independent of EIN2 and CTR via a phosphorylation cascade of kinase proteins, MKK4-5-9 (EC 2.7.12.2) → MPK3-6, which is mitogen activated [21, 32, 33]. In the presence of a signal, EIN3/EIL transcription factors are phosphorylated by MPK3-6 and do not interact with the F-box protein EBF (EIN3 binding F-box protein), preventing their degradation through the 26S proteasome. Thus, these factors that accumulate in the nucleus interact with target gene promoters and trigger different ethylene responses [33]. In addition, the exoribonuclease 5’-3’ EIN5 (EC 3.1.1.3.-), another positive regulator, promotes EBF mRNA decrease and thereby increases EIN3/EIL protein levels in the nucleus [34].

Ethylene signal transduction triggers substantial changes in the gene expression of plant cells. Promoter region analyses of the genes induced by ethylene led to the identification of *cis*-acting elements as well as the *trans*-acting protein EREBP (ethylene responsive element binding protein) family, which interacts with DNA and ERFs (ethylene response factors) [35–37]. Recent studies have demonstrated that EIN3/EIL are ERF1 (ethylene response factor 1) gene activators, constituting an ERF family member that establishes a hierarchy of ethylene-mediated signaling [38]. The homodimers EIN3/EIL interact with *cis-*acting elements in the *ERF1* promoter region that once transcribed and translated, interact with other *cis*-acting elements present in the promoter regions of target genes [38]. EIN3 can induce transcription not only of *ERF1* but also of other members of the AP2/ERF transcription factor superfamily [39].

The mechanism underlying environmental stress tolerance has been extensively studied in model plants in attempts to determine its impact on agriculture [40]. The metabolic pathways induced under drought in *A. thaliana* have been associated with abscisic acid (ABA)-dependent and ABA-independent pathways governing drought-inducible gene expression [41, 42] as well as the existence of an interconnection between both signaling pathways [43, 44]. Furthermore, advanced ABA and ethylene signaling research has revealed that under stress, both hormones act antagonistically among yield-impacting processes [45].

Although ethylene has been extensively studied in the plant senescence process, its role during drought-induced senescence is less well known. It has been demonstrated that under drought conditions, ethylene caused leaf abscission and consequently reduced water loss [46]. Under water deficit, ethylene production was paralleled by an increase and subsequent decrease in ACC, suggesting that water stress induced the *de novo* synthesis of ACC synthase, which is the rate-controlling enzyme along the pathway of ethylene biosynthesis. Moreover, ethylene and its metabolic process are important for activating plant responses to flooding and water deficit [47, 48]. It activates a signal transduction network that culminates in the synthesis of several transcription factors that regulate gene activation/repression during stress, such as ERF1 [41, 49, 50].

Despite important insights having been reported in ethylene signaling pathways, the available studies have not addressed the soybean *(Glycine max* [L.] Merrill), an economically important crop. This commodity is the second largest source of edible oil and the most important high-quality vegetable protein for feeding both humans and animals worldwide. However, deficiency in water supply can negatively impact this crop, reducing yields and posing threats to farmers and food production in several countries [51, 52].

Considering the important position that soybean occupies in the Brazilian economy, the second largest world soybean producer, *the Brazilian Soybean Genome Consortium* (GENOSOJA) was created to identify the genes related to different biotic and abiotic stresses. Because there have been no reports concerning ethylene molecular mechanisms in soybean, this work described the ethylene metabolic pathway *in silico* in the soybean genome using various databases. The gene expression profile data obtained from the GENOSOJA database was validated by RT-qPCR experiments, and determinations of free ACC levels and ethylene production in susceptible and tolerant soybean genotypes under water deficit conditions were also performed. Moreover, transcriptional regulation was studied by analyzing putative *cis*-acting elements present in the possible promoters. These data allowed for the inference of the first accurate *in silico* models for soybean ethylene biosynthesis and signaling, which facilitated a better understanding of the molecular mechanisms involved in this important phytohormone.

## Results and discussion

### *In silico* reconstruction of soybean ethylene molecular models

To evaluate the influence of ethylene in soybean water stress response, it was necessary to reconstruct the metabolic pathways to improve those available in public databases. Hence, we conducted an extensive search in the crop genome for genes previously associated with ethylene biosynthesis and signal transduction. Thus, a total of 322 genes were analyzed, of which 146 corresponded to model plants (74 from *Arabidopsis thaliana* and 72 from *Oryza sativa*) and 176 to *Glycine max* (Table 1). All of the soybean genes were mapped on their respective chromosomes (Additional file 1: Figure S1) and were functionally annotated (Additional file 1: Figure S2). The proteins identified in model plants *A. thaliana* (Additional file 2: Table S1 and S2) and *O. sativa* (Additional file 2: Table S3 and S4) as well as in *Glycine max* (Additional file 2: Table S5 and S6) were thoroughly characterized *in silico*, making possible the identification of the main characteristic domains. The soybean orthologous proteins in *A. thaliana* and *O. sativa* were investigated by BBH (best bidirectional hit) analysis, comparing the three species databases (Additional file 1: Figure S3; Additional file 2: Table S7 and S8). According to these data (see Additional file 3), accurate soybean models of ethylene biosynthesis and signal transduction have been proposed.

**Table 1:** Ethylene biosynthesis and signal transduction gene summary in different plants

Group	Number of genes		
	*Arabidopsis thaliana*	*Glycine max*	*Oryza sativa*
Biosynthesis	44	108	38
Signal Transduction	30	68	34
Total	74	176	72

The putative soybean proteins that participate in the metabolic pathways involved in ethylene biosynthesis and signaling mediated by this molecule are highly conserved, with domains that have already been described for their homologs in model organisms. The BBH experiment suggested a higher phylogenetic proximity of soybean to *A. thaliana*, corroborating that both are classified as dicotyledonous, although significant portions of these proteins are conserved in all three species. The ontological analysis indicated the same conclusion, showing that both function and molecular processes as well as the cell localization of these proteins were similar in different species.

### Soybean ethylene biosynthesis model

Based on the model for ethylene biosynthesis in *A. thaliana,* the 108 genes of soybean related to this metabolic route were divided into three groups: Yang cycle genes (21.3 %); ethylene biosynthesis (44.4 %); and ACC conjugation or degradation (34.3 %) (Additional file 2: Table S5).

**Fig. 1:**
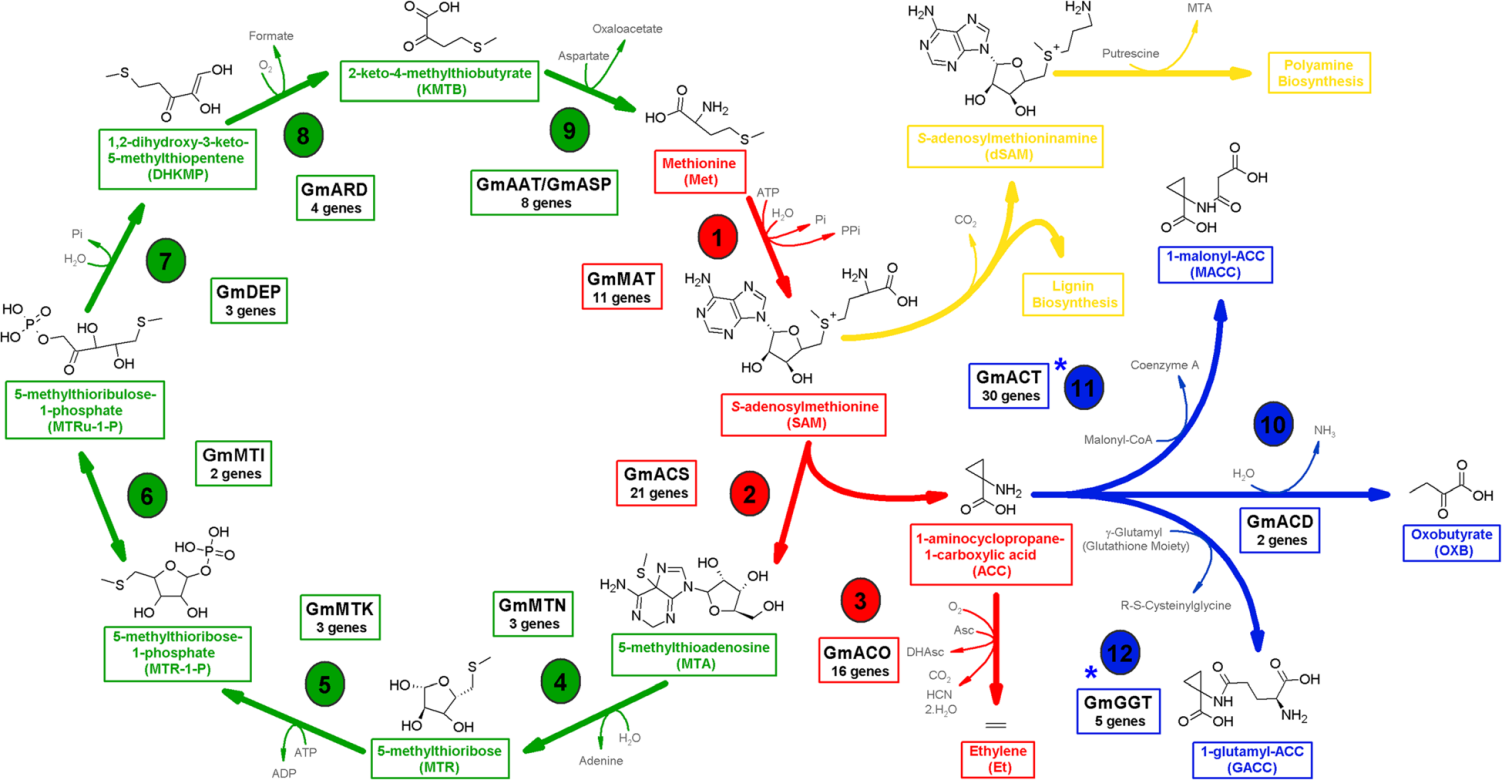
Soybean Model of Ethylene Biosynthesis. *In silico* experiments identified 108 proteins that could be involved directly or indirectly in soybean ethylene biosynthesis. In this putative model: *green* - Yang cycle; *red* - ethylene biosynthesis; *blue* - ACC (1-aminocyclopropane-1-carboxylic acid) degradation and conjugation with other metabolites (malonyl and glutamyl groups); *yellow* - lignin and polyamine biosynthesis (example of S-adenosylmethionine production deviation for other metabolic pathways). Enzymes: **1 - MAT** (methionine adenosyltransferase) or *SAMS* (S-adenosylmethionine synthetase); **2 - ACS** (1-aminocyclopropane-1-carboxylic acid synthase); **3 - ACO** (1-aminocyclopropane-1-carboxylic acid oxidase); **4 - MTN** (5-methylthioadenosine nucleosidase); **5 - MTK** (5-methylthioribose kinase); **6 - MTI** (5-methylthioribose-1-phosphate isomerase); **7 - DEP** (dehydratase-enolase-phosphatase complex); **8** - **ARD** (acireductone dioxygenase); **9** - **AAT** (amino acid transferase) or **ASP** (aspartate aminotransferase); **10** - **ACD** (1-aminocyclopropane-1-carboxylic acid deaminase); **11** - **ACT** (acyltransferase; N-malonyltransferase); **12** - **GGT** (γ-glutamyltranspeptidase). Other abbreviations: *Asc* - ascorbate; *DHAsc* - dihydroxyascorbate; *HCN* - hydrogen cyanide. The blue asterisks (*) present in numbers **11** and **12** indicate enzymes that could be candidates to play the roles described in the model, but their functions described *in vitro* and *in vivo* are not primarily associated with these metabolic pathways. Each enzyme is represented by a generic name (Additional file 2: Table S5)

Pommerrenig et al. [53] described a model for methionine recycling reactions through the Yang cycle in *Plantago* and *A. thaliana* [53]. Based on this work, we proposed an *in silico* model for this route in soybean, in which the homologs for all components were identified: MTN (5-methylthioadenosine nucleosidase; EC 3.2.2.16), MTK (5-methylthioribose kinase; EC 2.7.1.100), MTI (5-methylthioribose-1-phosphate isomerase; EC 5.3.1.23), DEP (dehydratase-enolase-phosphatase complex; EC 4.2.1.109 and 3.1.3.77), ARD (acireductone dioxigenase; EC 1.13.11.53 and 1.13.11.54) and AAT (amino acid transferase) or ASP (aspartate aminotransferase) (EC 2.6.1.1) (Fig. 1). Each of the identified enzymes had at least one ortholog in *A. thaliana* and/or *O. sativa* identified *in silico* through the BHH experiment, suggesting plausible conservation of the pathway in different plant species.

The first enzyme in the biosynthesis pathway, MAT (methionine adenosyltransferase) or SAMS*,* is responsible for the production of the AdoMet used for ethylene production and also for lignin and polyamine synthesis [10, 54]. Among the eleven MAT proteins in soybean, five were BHH-positive with possible orthologs in *A. thaliana* and/or *O. sativa*.

Subsequently, the classification of 21 soybean ACSs was proposed by Tucker et al. [55], who reported phylogenetic relationships with similar ACSs in *A. thaliana,* suggesting that they are expressed when the plant is infected by the nematode *Heterodera glycines* [55]. In our work, we studied the phylogenetic relationships of ACS amino acids residues between *G. max* and *A. thaliana* and also with its homologues in *O. sativa.* We also determined *in silico* the possible phosphorylation sites of the respective kinases (Additional file 1: Figure S4). The distribution of the sequences is similar to that presented by Tucker [55] because they are distributed uniformly, indicating high conservation between species. Moreover, although the sequences of GmACS#003, GmACS#013, GmACS#016 and GmACS#019 present high similarity with ACS, they are phylogenetically unrelated to the rest because differences were found in the catalytic domain. Therefore, these sequences were named ACS-like*, i.e.*, belonging to the family of AATs (amino acid transferases). Among the seventeen ACS sequences identified in soybean, six were possible orthologs of *A. thaliana* and/or *O. sativa,* of which two were determined to be type I (GmACS#011 and GmACS#014), two to be type II (GmACS#017 and GmACS#020) and two to be type III (GmACS#006 and GmACS#012) (Additional file 2: Table S7; Additional file 1: Figure S4).

Regarding the conversion of ACC into ethylene, sixteen *ACO* genes were identified in the soybean genome, with 6 of them encoding ortholog proteins in *A. thaliana* and/or *O. sativa* (GmACO#004, GmACO#006, GmACO#007, GmACO#008, GmACO#009 and GmACO#014) (Additional file 2: Table S7).

Furthermore, ACC can also be used in combination with malonyl and glutamyl in the synthesis of MACC (1-malonyl-ACC) and GACC (1-glutamyl-ACC) [25, 56]. We selected thirty possible candidate genes with this function in soybean, based on six *acyltransferases* (*ACT*) from *A. thaliana* and *O. sativa* (Additional file 2: Table S1 and S3). Five were considered BBH-positive with *A. thaliana* and/or *O. sativa* (GmACT#003, GmACT#006, GmACT#017, GmACT#020 and GmACT#023) (Additional file 2: Table S5)*.* It is important to emphasize that although most of the malonyltransferase enzymes play roles in fatty acids, they could also have N-malonyzation activity. Thus, it would be interesting to characterize them *in vitro* and *in vivo* after selecting them *in silico.* With regard to the formation of GACC, five γ-glutamyl transpeptidases (GGTs) were identified in soybean, and two of them (GmGGT#001 and GmGGT#003) were BBH-positive with *A. thaliana* and *O. sativa* (Additional file 2: Table S7)*.*

Finally, ACC could be the substrate of ACC deaminase (ACD) in soybean because we identified two genes that codified for homologous ACD enzymes in *A. thaliana* (GmACD#001 and GmACD#002)*,* of which only one was BHH-positive (GmACD#001) (Additional file 2: Table S7)*.*

### Model for soybean ethylene-mediated signal transduction

**Fig. 2:**
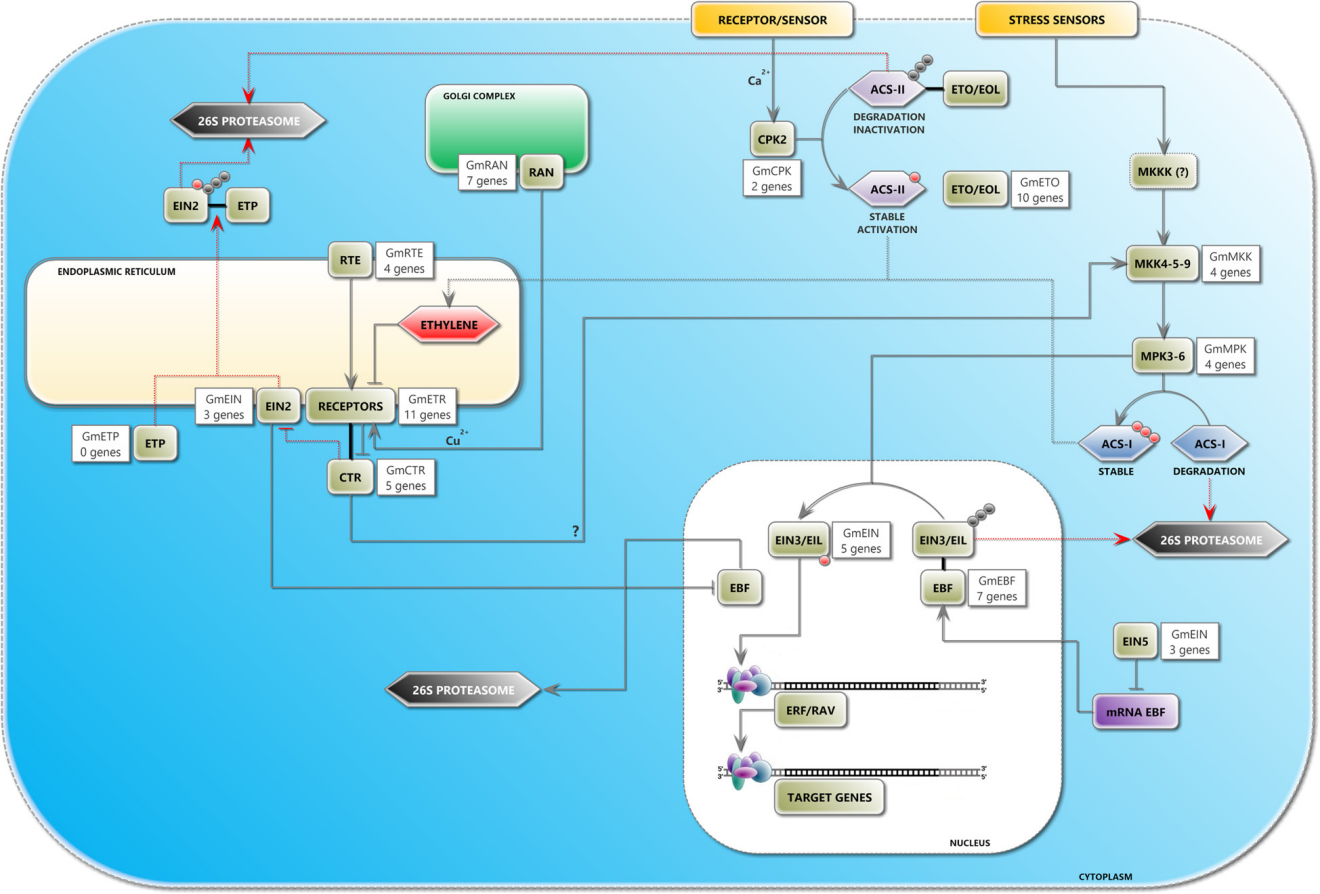
Soybean Model of Ethylene Signal Transduction. *In silico* experiments identified 68 proteins that could be involved directly or indirectly in soybean signal transduction initiated by ethylene. In this putative model, *brown rectangles* show the route-identified proteins in *A. thaliana,* and *white rectangles* show the soybean genes that encode proteins homologous to this plant model; *orange rectangles* illustrate membrane sensors that respond to biotic and abiotic stress in addition to receptors/sensors for endogenous signals (*i.e.*, other phytohormones); the *purple rectangle* represents mRNAs related to ETP proteins; the *rectangle with dotted outline* (accompanied by a question mark) represents a protein in this pathway that has not been identified in the studied plants; *blue and purple hexagons* represent ACSs types I and II, respectively; *black and red circles* correspond to ubiquitin and phosphate groups, respectively; *gray arrows* correspond to routes that occur in the presence of ethylene and/or biotic/abiotic stress; *dotted arrows in red and gray* represent pathways that occur in the absence of this hormone and routes that culminate in ethylene biosynthesis, respectively; *black lines* indicate interactions among proteins. Cellular compartments represented: *endoplasmic reticulum* (*beige*), *Golgi complex* (*green*), *nucleus* (*white*) and cytoplasm (*blue*). Symbols: **ACS**: 1-aminocyclopropane-1-carboxylic acid synthase; **CPK** (or **CDPK**): calcium-dependent protein kinase; **CTR**: constitutive triple response protein; **EBF**: EIN3 binding F-Box protein; **EIL**: EIN protein like; **EIN**: ethylene insensitive; **EOL**: ETO protein like; **ERF**: ethylene response factor; **ETP**: EIN2 targeting protein; **ETO**: ethylene overproducer; **MKKK** (or **MAPKKK**): MAP kinase kinase kinase; **MKK** (or **MAPKK**): MAP kinase kinase; **MPK** (or **MAPK**): mitogen-activated protein kinase; **RAN**: responsive to antagonist; **RAV**: related to ABI3/VP1; **RTE**: reversion to ethylene sensitivity. The route of intracellular signal transduction is initiated by the interaction of ethylene with a membrane receptor (encoded by *ETR* genes) and through the modulation of CTR activity, which regulates the activity of several genes, such as EIN3. The receptors with CTR (similar to the protein kinase RAF - MKKK) work similarly to negative regulators of the pathway and, in the absence of ethylene, suppress downstream positive components of signal transduction. The hormone binding blocks the receptors in an inactive conformation, reducing the repression of metabolic pathway-positive regulators [11]. In the absence of ethylene, CTR phosphorylates the EIN2 C-terminal domain, promoting its interaction with ETP F-box protein (not identified in soybean) and its subsequent degradation via proteasome 26S [30]. In the absence of EIN2 C-terminal phosphorylation (presence of the hormone), this domain is cleaved and moves to the nucleus, where it stimulates EIN3/EIL activity by EBF repression (stimulating the degradation of this F-box protein by unknown mechanisms), which in turn induces target genes transcription through some members of the AP2/ERF superfamily of transcriptional factors [31]. In addition to the interaction with the C-terminus of EIN2, EIN3/EIL activity can be influenced by the MKK4-5-9 → MPK3-6 phosphorylation cascade, which is CTR/EIN2-independent. In the presence of a signal, the EIN3/EIL transcriptional factors are phosphorylated by MPK3-6, preventing the interaction with EBF and their degradation via the 26S proteasome. Thus, EIN3 and EIL accumulate in the nucleus, interact with gene target promoters and trigger ethylene responses [33]. Another positive regulator is EIN5, a 5’-3’-exoribonuclease that promotes EBF mRNA decay, increasing the levels of EIN3/EIL in the nucleus [34]. Additionally, ethylene biosynthesis is also regulated. Possible receptors for endogenous signals (*i.e.*, other phytohormones) can induce the secondary metabolites accumulation (*i.e.*, calcium) in an intracellular environment and activate protein kinases (*i.e.*, CPK2), culminating in the stabilization of type II ACSs, an important enzyme in ethylene biosynthesis. Then, type II ACSs (in *A. thaliana* AtACS5 and AtACS9) are phosphorylated by CPK2, which prevents the interaction of these enzymes with ETO/EOL and their subsequent degradation by the 26S proteasome. This event induces an increase in ethylene production and the activation of signal transduction pathways [109]. Moreover, various stress conditions (biotic and abiotic) induce the activation of MAPK modules (in *Arabidopsis thaliana* MKK4-5-9 and MPK3-6). The MPK3 and MPK6 kinases are able to phosphorylate the C-terminal type I ACSs (in *A. thaliana* AtACS2 and AtACS6), which stabilize and protect these enzymes against 26S proteasome degradation [21]. There is no consensus regarding the direct participation of CTR in a route involving MPK3-6 [39]. The receptor activity is associated with two proteins: RAN, a copper carrier protein (copper is an important cofactor in receptor activity) [110]; and RTE, a protein with an unknown mechanism of action that facilitates the transition among active and inactive states of one receptor, ETR1 [33, 111]. Each protein is represented by a generic name: *EIN2*: GmEIN#002, GmEIN#004 and GmEIN#007; *EIN3*: GmEIN#001, GmEIN#005, GmEIN#006, GmEIN#008 and GmEIN#010; *EIN5*: GmEIN#003, GmEIN#009 and GmEIN#011; *MKK4*: GmMKK#001 and GmMKK#003; *MKK5*: without representatives identified in soybean; *MKK9*: GmMKK#002 and GmMKK#004; *MPK3*: GmMPK#003 and GmMPK#004; *MPK6*: GmMPK#001 and GmMPK#002; ***Receptors*** **:** *EIN4*: GmETR#002, GmETR#004, GmETR#008 and GmETR#011; *ERS1*: GmETR#001 and GmETR#007; *ERS2*: without representatives identified in soybean; *ETR1* : GmETR#003 and GmETR#006; *ETR2*: GmETR#005, GmETR#009 and GmETR#010 (Additional file 2: Table S6)

In this work, we identified 68 genes related to ethylene-mediated signal transduction. We found that 38.3 % of the proteins coded by these genes had orthologs in *A. thaliana* and/or *O. sativa* (Additional file 2: Table S8)*.* The main components of this signal route were represented because 32.4 % were specific receptors (ETR) and proteins important for receptor activity (RTE and RAN), 7.4 % were CTR, 4.4 % were EIN2 proteins, approximately 19.0 % were kinases (CPK, MKK, MPK), 7.4 % were EIN3/EINL transcription factors, 25.0 % were important in proteolysis routes (EBF and ETO), and 4.4 % were orthologs of EIN5 exoribonuclease, which is important for EIN3/EINL activity regulation (Fig. 2; Additional file 2: Table S6).

Four of the five ethylene receptors described in soybean were found to be homologs of ETR1 and ETR2 (subfamily I - GmETR#001, GmETR#003, GmETR#006 and GmETR#007) and of ERS1 and EIN4 (subfamily II - GmETR#002, GmETR#004, GmETR#005, GmETR#008, GmETR#009, GmETR#010 and GmETR#011) [57–60].

The receptors in soybean have four principal domains similar to those in *A. thaliana*: (i) receptor response regulation domain (PF00072); (ii) *histidine kinase A domain* (PF00512); (iii) *GAF domain* (PF01590); and (iv) *histidine kinase*^*−*^, *DNA girase B*^*−*^ and *ATPase-like* (PF02518). The different combinations of these four domains comprise the different families of receptors in soybean. For example, the ETR1 homologs have the four domains in their structure because homologs to ETR2 and EIN4 have only the (i), (ii) and (iii) domains and ERS1 has the (ii), (iii) and (iv) domains.

Regarding canonical ethylene signal transduction, we identified five soybean homologs of CTR1, four of RTE genes, seven RAN transporters and three homologs of EIN2 (GmEIN#002, GmEIN#004 and GmEIN#007) (Fig. 2). It is worth mentioning that homologs encoding the ETP proteins could not be found in soybean, suggesting either that other proteins are performing this role or that other mechanisms regulating EIN2 exist but have not yet been discovered. Furthermore, we also found five homologs of *EIN3/EIL* (*GmEIN#001*, *GmEIN#005*, *GmEIN#006*, *GmEIN#008* and *GmEIN#010*) and three of *EIN5* (*GmEIN#003*, *GmEIN#009* and *GmEIN#011*) in the *G. max* genome.

Finally, with regard to the main kinases and F-box proteins related to ethylene signal transduction, thirteen homologs of the kinases were found in the soybean genome, with four of them being homologs of *MKK4/MKK9*, four of *MPK3/MPK6* and five of *CPK2* as well as seven of *EBF* and ten homologs of *ETO/EOL* (Fig. 2).

### Transcriptional regulation of soybean ethylene genes

**Fig. 3:**
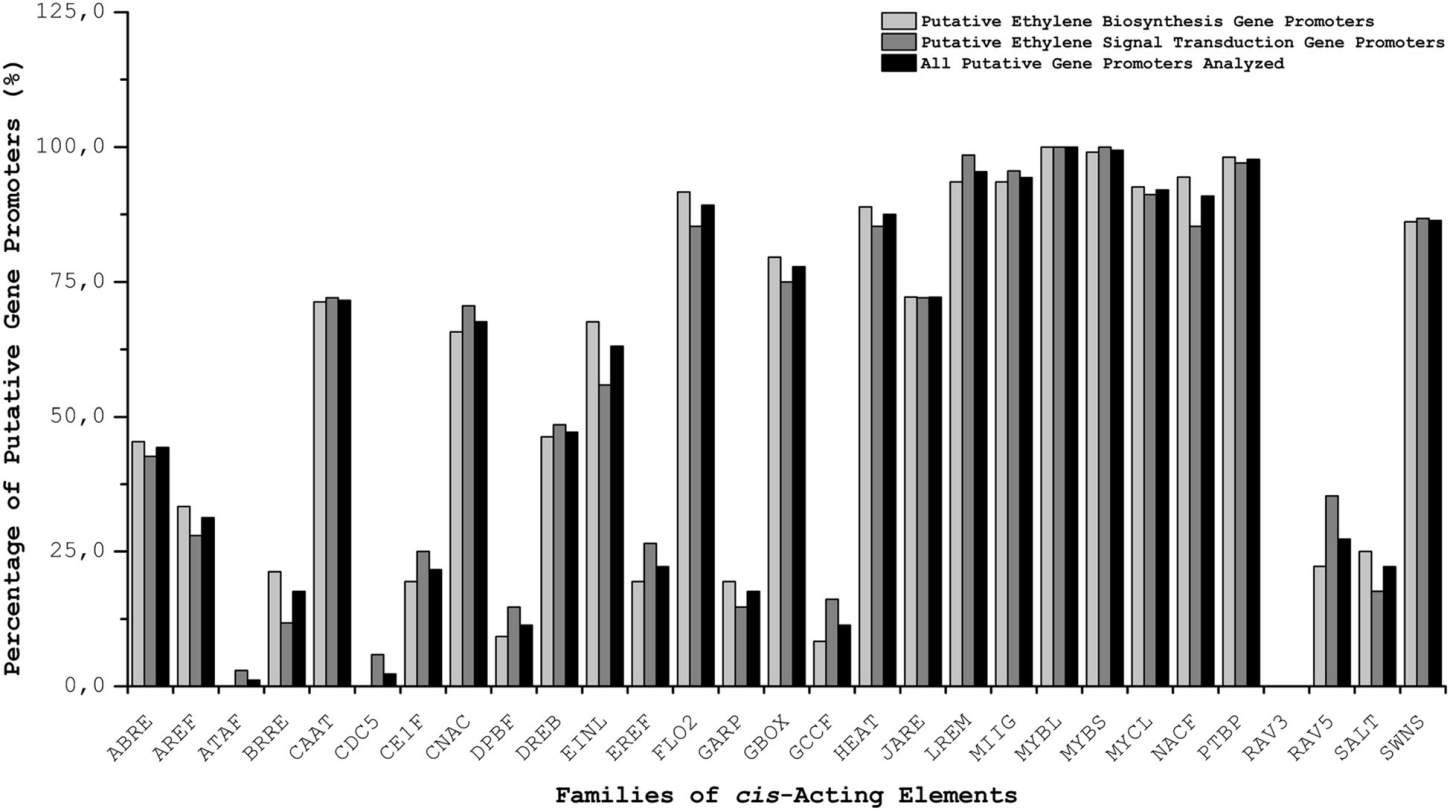
Distribution of *cis*-Acting Elements in Putative Soybean Gene Promoters. The graph shows the distribution of *cis*-acting elements in promoter regions of soybean genes, related to ethylene biosynthesis and signal transduction. The *cis*-acting element families identified were as follows: *ABRE* (ABA response elements); *AREF* (auxin response elements); *ATAF* (ATAF-like NAC domain containing proteins); *BRRE* (brassinosteroid response elements); *CAAT* (CCAAT binding factors); *CDC5* (*A. thaliana* CDC5 homologs); *CE1F* (coupling elements 1 binding factors); *CNAC* (calcium regulated NAC-factors); *DPBF* (*Dc3* promoter binding factors); *DREB* (dehydration responsive element binding factors); *EINL* (ethylene insensitive 3 like factors); *EREF* (ethylene response element factors); *FLO2* (floral homeotic protein APETALA2); *GARP* (MYB-related DNA binding proteins - Golden2, ARR, Psr); *GBOX* (plant *G-box*/*C-box* bZIP proteins); *GCCF* (*GCC-box* family); *HEAT* (heat shock factors); *JARE* (jasmonate response elements); *LREM* (light responsive element motifs, not modulated by different light qualities); *MIIG* (MYB IIG-type binding sites); *MYBL* (MYB-like proteins); *MYBS* (MYB proteins with single DNA binding repeat); *MYCL* (MYC-like basic helix-loop-helix binding factors); *NACF* (plant specific NAC transcriptional factors); *PTBP* (plant TATA binding protein factors); *RAV3* (3’-part of bipartite RAV1 binding site); *RAV5* (5’-part of bipartite RAV1 binding site); *SALT* (salt/drought responsive elements); *SWNS* (secondary wall NACS)

To understand better their transcriptional regulation mechanisms, we performed an *in silico* analysis of the putative promoter regions of the 176 soybean genes. We identified 14,385 elements in these putative promoters, corresponding to 29 *cis*-acting element families described in the literature for their transcriptional regulation in different plant species (Fig. 3; Additional file 4: Table S9).

As expected, all of the promoter regions contained elements from *PTPB* (plants *TATA-box*) and/or *CAAT* (*CCAAT-box*), suggesting that the analyzed sequences have a strong likelihood of being real gene promoters.

Apart from the *PTPB* and *CAAT* families, the most represented families in this analysis were those related to transcription factors MYB, MYC and NAC (Additional file 4: Table S9; Fig. 3) and to elements known for heat and light response (*LREM* and *HEAT,* respectively*)*. Interestingly, no *cis*-acting elements were found from the *RAV3* family in any of the putative promoters, indicating that there are possible variations in recognizing the sequence of the B3 domain that is representative of the RAV family in soybean. Another possibility could be that the regulation occurs because of the interaction of the AP2 domain with the *RAV5 cis*-acting element, which is broadly dispersed in the analyzed regions [61].

The families *EINL* (*ethylene insensitive 3-like*) and *GCCF* (*GCC-box*) of *cis*-acting elements are most likely directly related to the regulation of metabolic pathways in which ethylene plays a critical role. *EINL* and *GCCF* were present in 63.1 and 11.4 %, respectively, of the putative promoters analyzed (Fig. 3). The *DREB* (*dehydration responsive element binding factors*) and *EREF* (*ethylene response element factors*) elements are known for their involvement in the response to different stresses, and they were found in 47.2 and 22.2 %, respectively, of the analyzed sequences.

When we analyzed the *cis*-acting elements contained in the putative promoters of the ethylene biosynthesis genes, we observed that 67.6 % had *EINL* elements and that 8.3 % had *GCCF* elements. Moreover, other *cis*-acting elements that respond to other phytohormones were detected, of which the *JARE* family (*jasmonic acid*) was present in more than 70.0 % of the putative promoters, followed by the *ABRE* and *CE1F* (*ABA response*) families, which were present in 45.4 and 19.4 %, respectively, of the putative promoters. Moreover, 30.0 % of them have elements that respond to auxin (*AREF*) and 21.3 % to brassinosteroids (*BRRE*). Finally, the elements *DREB* and *EREF* could be detected in 46.3 and 19.4 % of the putative promoters, respectively.

Considering the group with an ethylene-mediated transduction signal, we observed the presence of *EINL* elements in 55.9 % and *GCCF* in 16.8 % of the putative promoters. We also detected the *JARE* element in more than 70.0 % of the sequences analyzed, *ABRE* and *CE1F* in 42.6 and 25.0 %, respectively, the auxin and brassinosteroid response elements in 28.0 and 11.8 %, respectively, and the *DREB* and *EREF* elements in 48.5 and 26.5 % of the putative promoters, respectively.

**Fig. 4:**
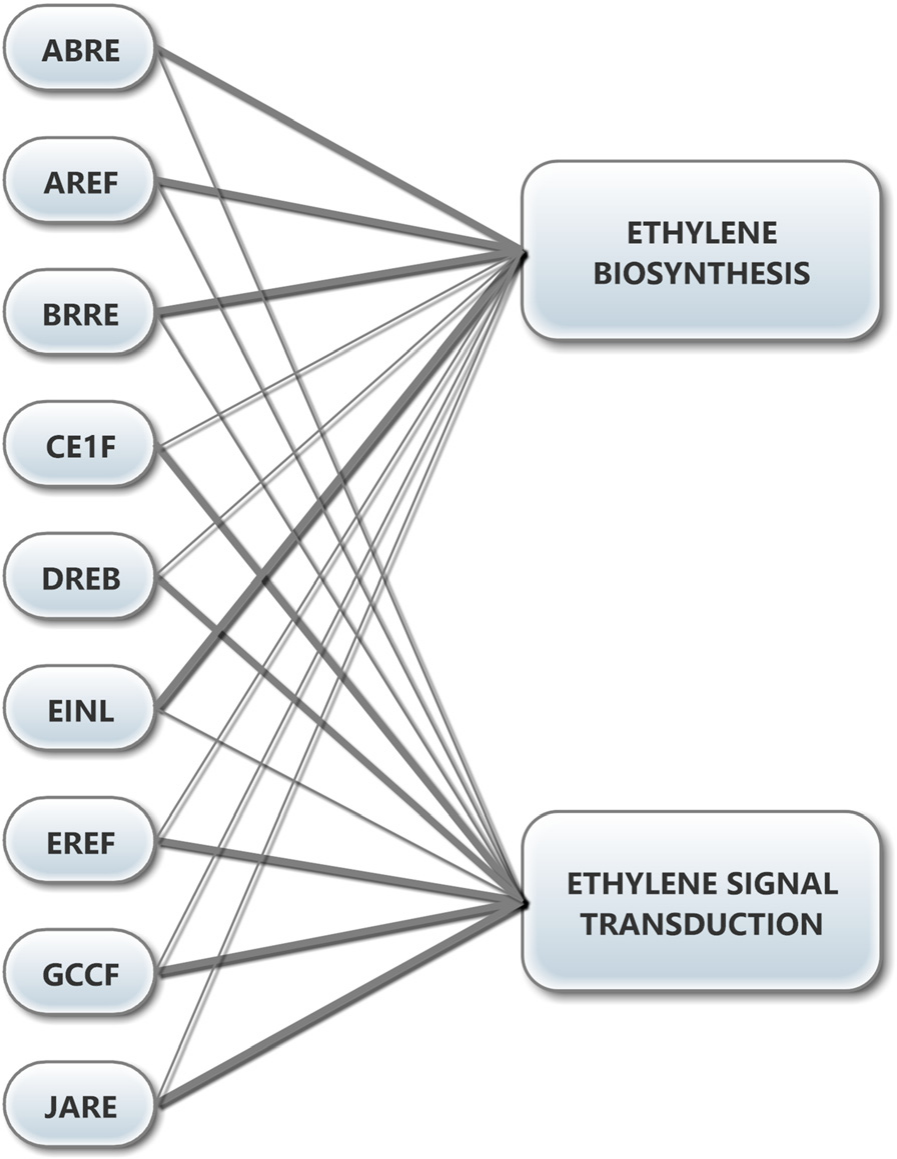
Distribution of *cis*-Acting Element Families Important in Ethylene Biosynthesis and Signaling in Putative Soybean Promoters. The diagram corresponds to the number of possible soybean promoters and the number of *cis*-acting elements present in each group analyzed: ethylene biosynthesis and signal transduction. The line thickness is directly related to the contribution of each family of *cis*-acting elements in each group: the thinnest lines correspond to the fewest number of elements and putative promoters that have them, and the thickest line corresponds to the highest number of elements and putative promoters that have them. *ABRE* - ABA response elements; *AREF* - auxin response elements; *BRRE* - brassinosteroid response elements; *CE1F* - coupling elements 1 binding factors; *DREB* - dehydration responsive element binding factors; *EINL* - ethylene insensitive 3 like factors; *EREF* - ethylene response element factors; *GCCF* - *GCC-box* family; *JARE* - jasmonate response elements

The analysis of the putative promoters showed that the activation or repression of the transcription of a gene in soybean is not likely to be regulated by isolated transcription factors but rather by the interaction of different proteins in a set of DNA-regulatory sequences. In accordance with this hypothesis, this study supported the results of other studies that had proposed crosstalk between the regulation of ethylene metabolism with other development mechanisms, homeostasis and response to various stresses. This affirmation was confirmed by the detection in the possible promoters of different *cis*-acting elements important for responses to other phytohormones, in addition to elements involved in different biotic and abiotic stress responses (heat shock, pathogen resistance, mechanic injuries, *etc.*). The presence of *cis*-acting elements in the 176 global soybean genes analyzed showed that the *JARE* elements were the most abundant, followed by *EINL*, *DREB* and *ABRE*. The putative promoter analysis indicated that each *cis*-acting element family could contribute in distinct ways to the regulation of the considered soybean genes: *ABRE*, *EINL*, *AREF* and *BRRE* are the most represented in the putative promoters of ethylene biosynthesis genes, and *JARE*, *DREB*, *EREF*, *CE1F* and *GCCF* are the most represented in the putative promoters of ethylene-mediated signal transduction (Fig. 4). Few (11.4 %) of the putative promoters presented *GCCF cis*-acting elements (responsive to ethylene)*,* whereas almost half of them had the very similar *DREB* element, which responds first to drought stress. These proportions were the same in the genes that were differentially expressed in drought stress. Recent ChIP (chromatin immunoprecipitation) experiments showed that the transcription factor ERF1 from *A. thaliana* could interact directly with both *cis*-acting element families. More interestingly, this transcription factor interacted with *GCCF* elements under biotic stress conditions and with *DREB* elements under abiotic stress conditions but never with both at the same time [62].

The data showed that 95.5 % of the putative promoters have the *LREM cis*-acting element (light-responsive elements, not mediated by different types of light) and that 87.5 % have *HEAT* elements (heat shock elements) (Fig. 3). In *A. thaliana,* the response to low light intensity could be regulated by ethylene and auxins (induction of AUX22, ACS6, ACS8, ACS9). Similarly, ethylene biosynthesis and ethylene signal transduction, regulated by phytochrome B, are affected by antiphase light and temperature cycles [63, 64]. Complementary studies with etiolated pea stems showed that in addition to light intensity, red light also regulates ethylene biosynthesis and gravitropism [65]. Additionally, mutants in receptors or orthologs of EIN2 sensitive to ethylene produce high levels of the gas, whereas *ctr1-1* mutants produce lower levels of ethylene than wild plants [66]. However, although the double mutants *ein3/einl1* have similar phenotypes to *ein2* mutants, they produce low levels of ethylene when grown under long day periods but high levels when grown under dark conditions and even lower levels of ethylene than in *etr1* and *ein2* mutants [67]. Thus, it is suggested that there is a parallel route to EIN3/EIL that is responsible for the negative control of ethylene biosynthesis, a mechanism that is light dependent. Transcriptional regulation could be associated with the light-responsive transcription factors that interact with *LREM* elements, which can modulate the response depending on the variation of the *G-box* sequences that commonly flank the *LREM* elements [68]. Because more than 77.8 % of the putative promoters have *GBOX* elements and are associated with a high rate of *LREM*, we believe that the mechanisms involving EIN3/EINL, its partners or regulated factors, and other light-responsive factors play important roles in the regulation of soybean ethylene biosynthesis.

Many differentially expressed transcripts identified in soybean transcriptomes have been described in the literature as being important in the response to drought. The functions of these transcripts could be associated with not only ethylene biosynthesis and signaling but also with other metabolic pathways. For example, the enzymes responsible for AdoMet production in ethylene biosynthesis also contribute to other metabolic pathways that are ethylene-independent. Plant polyamines in *A. thaliana* are involved in the response to different environmental stresses*,* and recent studies have indicated that polyamine signaling is involved in direct interactions with different metabolic pathways and intricate hormonal crosstalks, such as ABA regulation in response to abiotic stresses [69]. Because MAT (SAMS) enzymes provide the substrate for polyamine synthesis, it is very probable that these enzymes are induced by ABA in the response to abiotic stresses, as was demonstrated in tomato plants that had high levels of these enzyme transcripts under NaCl stress conditions and after ABA treatment [70]. Thus, it could be suggested that high levels of ABA are related to low levels of ethylene because of a possible redirection of AdoMet toward the biosynthesis of polyamines. We observed that among the *MAT* genes in soybean, 54.6 % have *ABRE* in their putative promoters, indicating induction of these genes by ABA in response to abiotic stresses.

The presence of elements responsive to other phytohormones must also be considered in the regulation of ethylene biosynthesis. Zhang and coworkers [71] demonstrated that ABA could induce the genes that encode the enzymes ACC synthase and ACC oxidase, stimulating ethylene biosynthesis and fruit ripening [71]. Additionally, studies have shown that one of the first actions of auxins is the induction of *ACSs*, which increase ethylene production [72]. Along with auxins, brassinosteroids and methyl-jasmonate could also induce ACO enzymes, increasing ethylene production in maize and olive plants [73, 74].

These studies with putative soybean promoters are important not only for a better understanding of ethylene signaling in this crop but also for the production of genetically modified plants with genes regulated under different stress conditions separately and/or simultaneously.

### Analysis and validation of soybean transcriptomes in water deficit conditions

#### Transcriptome databank analysis of water deficit contrasted with soybean genotypes

To investigate the expression of soybean genes, we studied the transcriptome of two cultivars with contrasting responses to drought stress (sensitive to drought BR16 and tolerant to drought EMBRAPA48). The plants were grown hydroponically and under different water stress conditions. The transcriptomes, provided by the GENOSOJA project, were constructed using *subtraction library hybridization* (SSH), which detects differential expression of transcripts under water stress. In this database, 40.9 % of the genes identified were expressed differentially in at least one of the listed situations. Among them, 43.1 % were related to ethylene biosynthesis and 56.9 % to its signal transduction (Additional file 1: Figure S5 and S6). Furthermore, we found that 25.0 % of differentially expressed genes were detected in sensitive BR16, 47.2 % were detected in drought-resistant EMBRAPA48, and 27.8 % were present in both cultivar databases. These contrasting results might be explained by the genetic basis of each cultivar providing the relative variations in the gene expression or by a discrepancy between the obtained unique sequences and the cultivar databases (42.3 million unique sequences generated, of which 27.8 % are from BR16 and 72.2 % from EMBRAPA48) [75].

We observed that 37.5 % of the differentially expressed genes were detected uniquely in roots (among which 3.7 % were from BR16 and 96.3 % were from EMBRAPA48), 26.4 % were detected exclusively in leaves (among which 84.2 % were from BR16, 10.5 % were from EMBRAPA48, and 5.3 % were found in both cultivar databases), and 36.1 % were expressed in both roots and leaves. These results, together with the normalized data presented (Additional file 1: Figure S7), suggested that the expression of genes in the roots was preferentially observed in the drought-tolerant EMBRAPA48, whereas in the leaves, the differential expression was more proportionate depending on the group of genes and the duration of stress.

Furthermore, the expression of both groups of genes was analyzed. We observed that 28.7 % of the biosynthesis genes were expressed in roots and leaves of the sensitive and tolerant cultivars. Among them, 16.1 % were expressed in only sensitive BR16, mainly in the leaves. Conversely, 43.9 % differential expression was detected exclusively in tolerant EMBRAPA48, mainly in the roots (Additional file 1: Figure S5).

In ethylene-mediated signal transduction, 61.8 % of the genes were differentially expressed under water stress conditions. In the sensitive cultivar, 33.3 % were differentially expressed, mostly in the leaves, and in the tolerant cultivar, 42.9 % had a differential expression, mainly in the roots (Additional file 1: Figure S6).

### Transcriptome functional validation of the candidate genes

To validate the data obtained *in silico*, the levels of some differentially expressed genes were assessed by RT-qPCR in both leaf and root tissues exposed to drought stress. The plants were grown under the same conditions as those used for transcriptome analysis. The *C*_*t*_ (cycle threshold) values obtained are listed (Additional file 5: Table S10 and S11).

**Fig. 5:**
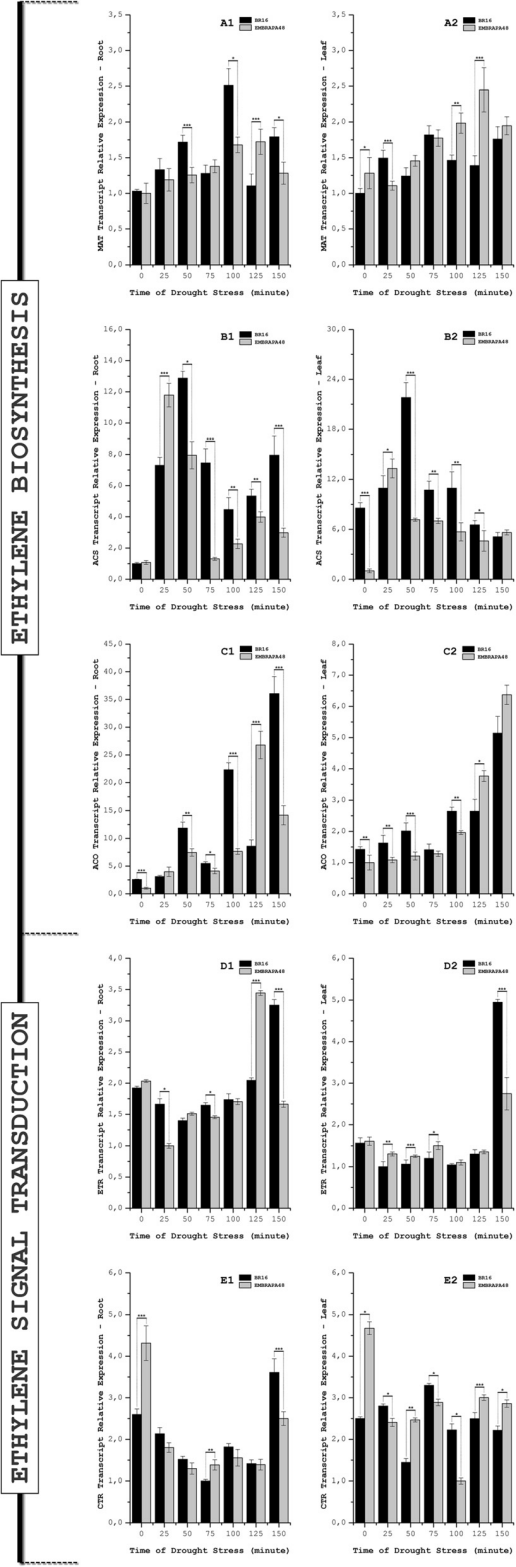
Expression of Ethylene-Related Genes in Soybean Under Drought Stress Conditions. The graphs show the expression levels, obtained by RT-qPCR, of five soybean genes related to ethylene biosynthesis [*MAT* (**a**), *ACS* (**b**) and *ACO* (**c**)] and ethylene signal transduction [*ETR* (**d**) and *CTR* (**e**)]. The expression of these genes in the experiment was compared in roots and leaves of soybean cultivars BR16 and EMBRAPA48 after different durations of drought stress. In the column graphs *1* and *2*, the statistical analysis was performed by comparing similar tissues in both cultivars under the same conditions of drought stress (same durations). The asterisks represent the level of statistical significance: (*) *p* ≤ 0.05; (**) 0.01 ≤ *p* < 0.05; (***) 0.001 ≤ *p* < 0.01. Each dot represents the average amount (± standard error) of three experimental replicates (same sample) in three biological samples (different plants), totaling nine replicates. The standard error is not presented with some of the dots because their absolute values are lower than the scale. After normalization based on housekeeping genes, the values given in the graph are relative to the lowest expression, whose value was set at 1 (one). Information about the target genes is presented (Additional file 6: Method S1)

The expression of the genes *MAT*, *ACS* and *ACO* were found to have the same differential trends as the data obtained *in silico,* although with variations in the expression profiles (Fig. 5a, b and c. Additional file 1: Figure S8a, S8b and S8c). This result could be due to limitations in the construction of the GENOSOJA subtractive libraries and general experimental variations. As an example, the expression of *ACS* is different in both cultivars and tissues with RT-qPCR, but it was detected in only the transcriptome of the roots of EMBRAPA48.

Induction kinetics analysis of soybean *ACS* and *ACO* genes confirmed the temporality of the metabolic reactions catalyzed by these enzymes in both cultivars because *ACS* gene expression reached its peak earlier than that of the *ACO* gene (Fig. 3b and c). Furthermore, when comparing the two soybean varieties, the expression of these two genes was observed earlier in the drought-tolerant cultivar. This fact could be evidence of ethylene participation in soybean responses to water stress.

The same analysis was performed with the genes coding ethylene receptors (*ETR*) and for the protein kinase *CTR* (Fig. 5d and e. Additional file 1: Figure S8d and S8e). Few differences were observed in the expression patterns of the transcripts of these genes between the cultivars. In the roots of both cultivars, there was a reduction in the level of *ETR* transcripts, comparing stressed and non-stressed plants. The maximal expression was achieved under the longest periods of stress. In the same tissue, the transcripts of *CTR* were reduced, with a significant increase detected only after 150 min of water deficit in both cultivars. In the leaves, when comparing the stress and no-stress conditions, we were able to observe a slight reduction in the levels of transcripts of *ETR* in the first 125 min of drought and a peak elevation at the end of the analysis (150 min). Relative to the *CTR* transcripts, it was observed that the expression of the drought-tolerant cultivar was higher in the non-stressed state (time zero).

### Levels of free ACC and ethylene production

To compare and correlate the data obtained *in silico* with the physiological data, we assessed the levels of free ACC and ethylene in both BR16 and EMBRAPA48 cultivars. The plants were grown under similar conditions as those used for the analysis of transcriptomes. The physiological data showed that both of the cultivars suffered under the water deficit but that the tolerant cultivar responded better, exhibiting increases in the photosynthetic rate, stomatal conductance and evapotranspiration after 75 min of stress (Additional file 1: Figure S9). The water consumption (WUE) showed that before 75 min had elapsed, the sensitive cultivar was using its water resources better than the tolerant cultivar, but subsequently, the situation was reversed; thus, the stress caused a greater impact on the susceptible cultivar.

**Fig. 6:**
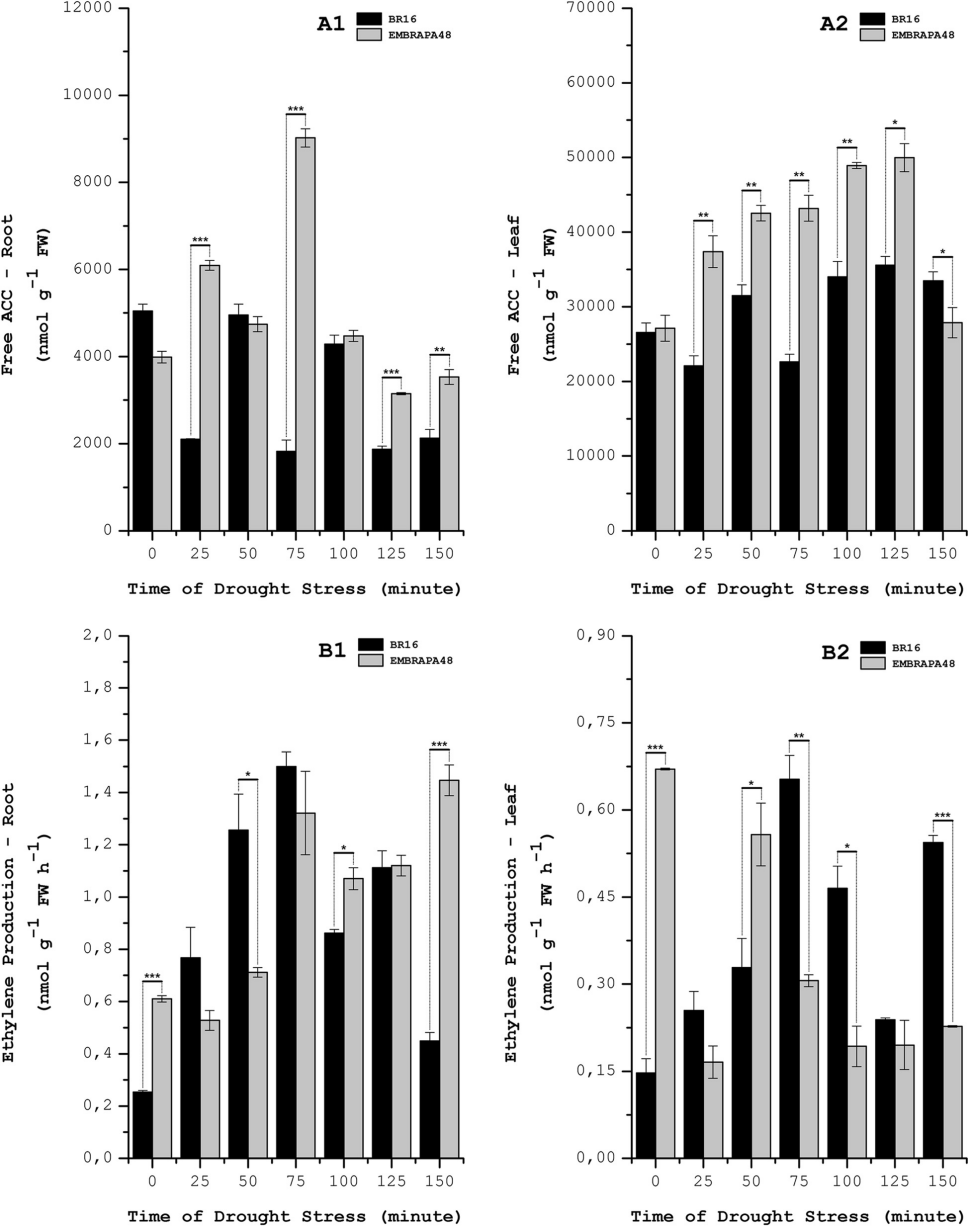
Levels of Ethylene Production and Free ACC in Soybean Under Drought Stress Conditions. Values were determined for ethylene production and free ACC (1-aminocyclopropane-1-carboxylic acid) in roots and leaves of soybean cultivars BR16 and EMBRAPA48 after the application of different durations of drought stress. The codes ***a1*** and ***a2*** represent levels of free ACC; ***b1*** and ***b2*** represent levels of ethylene production. The statistical analysis was performed by comparing similar tissues in both cultivars under the same conditions of drought stress (same durations). The asterisks represent the level of statistical significance: (*) *p* ≤ 0.05; (**) 0.05 < *p* ≤ 0.01; (***) 0.01 < *p* ≤ 0.001. Each dot represents the average amount (± standard error) of three replicates in different plants. The standard error is not presented with some dots because their absolute values are lower than the scale

We analyzed the levels of free ACC and ethylene production and found, in general terms, that free ACC was mostly increased in the leaves, whereas ethylene was mostly increased in the roots (Fig. 6 and Additional file 1: Figure S10). We observed that the EMBRAPA48 cultivar had higher levels of free ACC in the leaves and variable levels in the roots. In the roots of non-stressed plants (time zero), the free ACC was higher in BR16 plants, and the ethylene production was higher in the EMBRAPA48 cultivar (Fig. 6a and b. Additional file 1: Figure . S10a and S10b), whereas in the leaves, the level of free ACC was the same in both cultivars, and the quantity of ethylene production was higher in EMBRAPA48 (Fig. 6 a2 and b2). Except for the period of 25–50 min of stress, it was observed that both free ACC and ethylene production exhibited cyclic behavior in both the leaves and roots of BR16: when free ACC increased, ethylene decreased, and vice versa. Additionally, in BR16, we found that in both tissues, the peak of ethylene production (75 min) corresponded to the lowest value of free ACC. In the EMBRAPA48 cultivar, this cyclic pattern was much less evident. We observed that the highest peaks of free ACC were found after 75 min in the roots and after 125 min in the leaves, whereas the maximal production of ethylene corresponded to 75 and 150 min of stress, respectively. In the leaves, the maximal peak of ethylene production occurred at time zero.

When we compared the levels of ethylene production and free ACC, two different situations were observed. First, an increase in free ACC coincided with an increase in ethylene production. Although the hydrolysis of ACC aggregates remains contradictory, the high level of free ACC could be explained by the degradation of these aggregates of malonyl-ACC into free ACC, accompanied by an increase in free ACC production and the conversion of AdoMet into ACC by the ACS enzyme. Thus, the levels of free ACC would exceed the capacity of ACO enzymes to convert it into ethylene, which would be present at its maximal level [23, 76, 77]. Conversely, we observed a reduction of the levels of free ACC, together with a reduction in ethylene production. This finding could be explained by the formation of malonyl-ACC and glutamyl-ACC, accompanied by the degradation of the ethylene precursor by ACD enzymes. To support this conclusion, we simultaneously detected the differential expression of GmGGT#002 in the roots and leaves of both cultivars and GmACD#001 in the roots of EMBRAPA48. To understand this trend better, it would be necessary to characterize the molecular pathways involved in ACC conjugation and degradation *in vitro* and *in vivo* to determine the precise mechanisms underlying the regulation of ethylene biosynthesis in response to diverse signals, in addition to the identification of the actual role of the formation of ACC aggregates in this case.

In our work, transcriptome analysis, RT-qPCR and ethylene production revealed that ethylene synthesis depended on the tissue analyzed. After 75 min of water deficit, the maximal production of ethylene was observed in leaves of BR16, whereas after the same period of water deficit, in the leaves of EMBRAPA48, ethylene exhibited a significant decrease. In the roots, both cultivars had high levels of ethylene production (Fig. 6). Together with only the tolerant cultivar displaying an increase in stomatal conductance, photosynthetic rate and transpiration after the same stress period (Additional file 1: Fig. S9), these findings indicated that in this situation, leaves and roots undertake different responses to ethylene. Additionally, we can suggest that in the leaves, ethylene production could be associated with the response to drought stress because ethylene could regulate stomatal closure [78].

Nonetheless, studies have shown that the levels of this phytohormone are low when plants are exposed to water deficit [79, 80]. These conflicting observations could be attributed to the system in which the soybean plants were grown. The plants were grown hydroponically, with the roots submerged in a nutrient-containing solution. Some studies have shown that variations in gene expression could occur when hydroponic and soil cultures were compared [42]. Thus, it is believed that hydroponically grown roots have molecular responses similar to those of roots grown under flooding conditions and that when subjected to water deficit, they exhibit molecular responses different from those shown by roots grown via soil culture.

Additionally, other works have reported that in plants grown under flooding conditions, the levels of ethylene production were higher than those obtained under water deficit conditions [81]. We also believe that a natural elevation of the temperature caused by rapid water loss could be explained by an increase in ethylene biosynthesis because the activity of enzymes was also rapidly increased, as shown by Antunes and Sfakiotakis [82]. Because ethylene diffusion is more rapid in the air than in liquid (Hoagland’s solution) and because water deficit and dehydration are more rapid under hydroponic conditions, we believe that the plants do not have sufficient time to begin molecular responses before desiccation occurs. One explanation could be that when short intervals of water deficit (25–50 min) are applied, ethylene biosynthesis and signal transduction remain similar to those under normal growing conditions. Therefore, when the stress duration is increased, the signal transduction could be strongly decreased. In fact, the plants were switched from flooding stress to water deficit stress, possibly activating different responses that substituted for the normal water deficit responses because we observed the differential expression of many genes even before the stress was administered. Thus, the analysis of the GENOSOJA database would be best complemented by next-generation sequencing experiments to replace the SSH methodology and cultivation in pot systems, instead of under hydroponic conditions.

## Conclusions

This study was the first to propose accurate models for ethylene biosynthesis and signaling in the soybean. Based on the currently available databases, soybean genes and proteins homologous to almost all of the components of the pathways featured in *A. thaliana* were identified, with the exception of the *ETP* gene. The *cis*-acting elements present in soybean putative promoters were described to infer possible models and the regulation of signaling pathways linked directly to ethylene as well as their communication with other metabolic routes. RT-qPCR experiments were important to the validation of soybean transcriptome data and allowed for the evaluation of the induction kinetics of *ACS* and *ACO* soybean genes. Finally, changes were observed in the levels of production of ethylene and its precursor (in its free form) in soybean cultivars under water stress conditions.

By the integration of all data, many inferences could be made, among which the involvement of ethylene in soybean water stress responses stands out. Furthermore, this work showed that regulation of the ethylene-mediated response could be influenced by diverse exogenous and endogenous factors, indicating that the balance of these various factors determines the quality and intensity of different stimuli responses. Further studies are necessary to continue elucidating *in vivo* molecular mechanisms involved in ethylene coordination in soybean both to confirm our observations and to facilitate biotechnological strategies for the improvement of cultivar tolerance to various stresses.

## Methods

### Functional annotation

Based on the Genbank TAIR (*The Arabidopsis Information Resource*; http://www.arabidopsis.org/) [83], we selected the genes related to ethylene biosynthesis and signaling in *Arabidopsis thaliana*. A BLAST (*Basic Alignment Search Tool)* search was performed with the amino acid (amino acid sequences, equal or over 200 bits score, against protein sequence databases: *Glycine max* [L.] Merrill (*GENOSOJA*: http://www.lge.ibi.unicamp.br/soybean/; *SoyBase*: http://soybase.org/; *Phytozome version 9.1*: http://www.phytozome.net/) and *Oryza sativa* Nipponbare (*Rice Genome Annotation Project*; http://rice.plantbiology.msu.edu) [84–86].

Subsequently, 176 soybean genes were ranked in three groups according to their ontology with *Blast2GO* software (Gene Ontology) [87]: (i) *cell componen*t, with suggestions about their active locations at the cellular and macromolecular complex substructure levels; (ii) *molecular function*, with descriptions of their the catalytic activity or binding at the molecular level; and (iii) *biological processes*, with descriptions of their biological objectives according to one or more ordered sets of molecular features. For this purpose, the soybean nucleotide sequences of each gene were processed with the aid of the *BlastX* tool (used to search the database according to the nucleotide sequences translated into all six possible reading phases) using the *A. thaliana* protein sequences as a database and only selecting those with an *e-value ≤ e*^*−10*^. After the annotation, the functionality of the sequences was analyzed with the aid of the online tool *InterproScan version 5.0* [88] and finally determined by the online software *GO Ontology-Slim* [89].

The protein domain analysis of the amino acid sequences of the genes selected in the three studied organisms was performed using the *PFAM* (*Protein Family*; http://pfam.xfam.org/) bioinformatic tool [90]. The selection parameter (*e-value* < 1.0) used was the same one defined by this program’s website.

The ideogram representing the location of the 176 soybean genes analyzed in the 20 chromosomes was built in proportion to the chromosome size (1.0 cm corresponds to 5.0 megabase), taking into account the location of each gene and the DNA strand in which they are localized (sense and antisense). The positions of the centromeres and the size of each chromosome were obtained from a reference genome [91].

The sequence alignment analysis and dendrogram construction were performed with the programs *BIOEDIT version 7.0.9.0* and *MEGA version 5*, respectively. The Neighbor-joining analyses were used to calculate the distance matrices for dendrogram construction. Bootstrap analysis with 10^4^ replicates was performed to test the robustness of the internal branches. The proposed models for ethylene biosynthesis and signaling in soybean were obtained from the *SoyCyC version 3.0* (*Soybean Metabolic Pathway Database*; *Soybase*; http://www.soybase.org:8082/) and *KEGG* (*Kyoto Encyclopedia of Genes and Genomes*; http://www.genome.jp/kegg/) [92, 93].

The possible protein phosphorylation sites by MAP kinases (MAPK) and calcium-dependent protein kinase present in the 1-aminocyclopropane-1-carboxylic acid synthase (ACSs) type I and type II, respectively, were determined by the online program *NetPhos version 2.0* (http://www.cbs.dtu.dk/services/NetPhos/) [94]. The presence of possible phosphorylation sites was analyzed in the C-terminal region using the amino acids tyrosine, serine and threonine.

Each protein sequence identified in the soybean databases (*A* sequence) was compared individually with those from *A. thaliana* (*TAIR*) and rice (*Rice Genome Annotation Project*) to obtain the homologous *B* and *C* sequences, respectively. The BBH (*Best Bidirectional Hit*) criterion was used, and positive hits were obtained when *B* and/or *C* sequences were compared with the soybean database; the best similarity was with the *A* sequence. Gene duplication was considered to avoid false negatives [95].

The presence of *cis*-acting elements in putative promoter regions was examined [2000 pairs upstream of the open reading frame bases (ORF)] for each soybean gene selected for this study. This analysis was performed using the bioinformatics tool *MatInspector version 8.0* (Genomatix®) using “*plants*” as matrix group, “*0.85*” as the value for the similarity of the main bases that constitute each *cis*-acting element (core similarity), and “*Optimized +1*” as the value for the similarity matrix (similarity matrix) [96].

The expression of each gene involved in the biosynthesis and signaling of the ethylene metabolic pathway was accessed in the GENOSOJA database [75]. The gene expression levels were represented in graphics indicating the *FPKM* (fragments per kilobase of exon per million fragments mapped) normalized read counts for each gene that was differentially expressed in the twelve cDNA libraries (25–50, 75–100 and 125–150 min of drought stress).

### Plant growth and physiological parameters

Soybean seeds from BR16 and EMBRAPA48, which are sensitive and tolerant to water deficit [97, 98], respectively, were germinated on filter paper (*Germitest*) for seven days in a growth chamber at 25.0 ± 1.0 °C and 100.0 % relative humidity (RH). The seedlings were transferred to 36 L boxes containing 50.0 % Hoagland’s solution [99], which was continuously aerated and replaced weekly. The plantlets were grown until V5 stage [100] in a greenhouse under a natural 12 h photoperiod at 30.0 ± 5.0 °C and 60.0 ± 10.0 % RH. The experimental design was a randomized complete block in a 2x7 factorial arrangement involving two cultivars (BR16 and EMBRAPA48) and seven water deficit periods (0, 25, 50, 75, 100, 125 and 150 min), respectively, with three replicates. The stress was imposed by removing the plants from the hydroponic solution and leaving them without nutrient solution for up to 150 min under air exposure conditions. For each water deficit period, root and leaf samples were collected from three plants, pooled and frozen in liquid nitrogen before storage at −80 °C.

The photosynthetic rate (*A*), photosynthetically active radiation (*PAR*), internal CO_2_ concentration (*C*_*i*_), stomatal conductance (*g*_*s*_) and transpiration rate (*E*) were evaluated using a *LI-6400* Portable Photosynthesis System (Li-Cor, Inc.). The parameters were measured in triplicate on the youngest trifoliate leaf that was totally expanded under a photon flux density of 1300 μmol m^−2^ s^−1^. The temperature variation (ΔT) was measured by the difference between the air (*T*_*ar*_) and leaf (*T*_*leaf*_) temperatures. The water use efficiency (*WUE*) was determined by the ratio between *A* and *E*. The data were statistically analyzed by *ANOVA* using the *SAS* and *SANEST* (*Statistical Analysis System version 8.0*) softwares, and the treatments were compared by *Tukey’s* test (*p* ≤ 0.05).

### Total RNA extraction and quantitative real time PCR (RT-qPCR)

Total root and leaf RNA from BR-16 and EMBRAPA48 of each treatment was extracted in triplicate using the Trizol (Invitrogen) protocol and treated with DNAse I (Invitrogen, Inc.). Total mRNAs were utilized as templates for cDNA synthesis using the enzyme *Moloney Murine Leukemia Virus Reverse Transcriptase* (M-MLV RT) (Invitrogen, Inc.).

Quantitative real-time PCR was performed to validate the genes related to ethylene biosynthesis (*MAT*, *ACS* and *ACO*) and signaling (*ETR* and *CTR*) pathways in soybean. Primers were designed by *Primer 3 Plus* [101] software and checked for the presence of putative amplicons from 120 to 200 pb and melting temperature (T_M_) of 60.0 ± 2.0 °C (see Additional file 6: Method S1). To establish the normalization factor, two reference genes were used for root samples (*ACT11* and *UBC2*) and two for leaf samples (*CYP2* and *ELF1A*) [102, 103]. All experiments were carried out in experimental and biological triplicate. The quantitative real-time PCR amplifications were performed using the *ABI Real Time PCR System 7500 Fast* (Applied Biosystem, Inc.) thermal cycler with a comparative cycle threshold (ΔΔC_t_*). Rox Plus SYBR Green Master Mix 2X* (LGC Inc.) was combined with 4.0 or 10.0 μM of each primer (sense and antisense) and 2.0 μL of cDNA (40 or 80-fold dilution) for each experimental condition (Method S1). The PCR cycling conditions were 95 °C for 15 min to activate the hot-start Taq DNA polymerase, 40 cycles at 95 °C for 30 s, 60 °C for 30 s and 72 °C for 3 min (final extension). The raw fluorescence data for all runs were imported into the *Real-Time PCR Miner* software [104] to determine the *C*_*t*_ value and the PCR efficiency. The *C*_*t*_ values were converted by *qBASE v.1.3.5* software [105]. The statistical analysis was performed using the *REST 2009* (*Relative Expression Software Tool* - Qiagen, Inc.) software [106] in two ways: first by comparing the relative gene expression values between both cultivars in the same tissue under the same stress conditions and second by comparing the control (without stress) with the stressed samples of the same cultivar.

### Determination of ethylene production

For ethylene analysis, 0.5 g root and leaf samples were collected for each stress period in 50 mL glass recipients and sealed with a silicone lid. After 24 h, the ethylene analysis was performed. First, a 1.0 mL sample of each treatment was obtained using a gastight syringe, and its concentration was determined by a gas chromatograph (GC) equipped with a flame ionization detector (FID), as described by Mainardi et al. [107]. The GC column used was HP-Plot Q (30 m, D.I. 0.53 mm), and the injection conditions were a pressure of 20.0 psi for 2 min, ventilation flux of 20.0 mL.min^−1^ after 30 s and injector temperature of 200 °C. An isothermal program was run at 30 °C, employing constant fluxes of helium gas of 1.0 mL min^−1^, a detector temperature of 250 °C and detector air and hydrogen fluxes of 400.0 mL min^−1^ and 40.0 mL min^−1^, respectively. The ethylene production was estimated in relation to the injection of 0.1 μL L^−1^ of ethylene in synthetic air (Air Liquid Ltd.), and it was represented in nmoles for grams of fresh weight for hour (nmol g^−1^ FW h^−1^).

### Determination of free 1-Aminocyclopropane-1-Carboxylic Acid (ACC)

Liu and coworkers [108] proposed the method for the determination of free ACC [108]. The samples were composed of roots and leaves from both cultivars, collected in triplicate, from different plants, stored in 50.0 mL Falcon tubes and frozen in liquid nitrogen (N_2_). Approximately 0.5 g of each sample was crushed with N_2_, and the powder was transferred to 5.0 mL of a 60.0 % methanol solution (v/v). The samples were stirred for one hour under ambient temperature and centrifuged at 14000 x g for 10 min at 25 °C. The supernatant was transferred to another tube. The residue was resuspended in 200.0 μL of ultrapure water and transferred to a 1.5-mL microcentrifuge tube, to which was added 300.0 μL of 200.0 mM borate buffer at pH 8.0 and 360.0 μL of 1.0 mM fluorescamine dissolved in acetone. The mixture was vigorously stirred, maintained at 25 °C for 10 min and then filtered through a 0.45 micron porous membrane into a 2.0 mL glass vial. A 20.0 μL aliquot of the filtered mixture was injected into a liquid chromatograph coupled with a fluorescence detector (Agilent 1100). The sample was eluted through a *C18 Luna* column (5.0 microns, 300 x 4 mm, *Supelco, Sigma-Aldrich, USA*), and the effluent was monitored at an excitation wavelength of 378 nm and an emission wavelength of 475 nm. The results were calculated according to an external standard curve of standard ACC (Sigma-Aldrich, USA) in the range from 0.1 to 10.0 μg. The determination of free ACC is given in nmoles for gram of fresh weight (nmol g^−1^ FW).

## Additional files

Additional file 1:Supplementary Figures (Figs. S1-S10).
**Figure S1. ** Soybean Chromosomal Ideogram; **Figure S2**. Gene Ontology Classification; **Figure S3**. Protein Orthology by Best Bidirectional Hit (BBH) Analysis; **Figure S4**. ACSs Classification; **Figure S5**. Differential Expression of Genes Related to Soybean Ethylene Biosynthesis in Transcriptomes Under Drought Stress Conditions; **Figure S6**. Differential Expression of Genes Related to Soybean Ethylene Signal Transduction in Transcriptomes Under Drought Stress Conditions; **Figure S7**. Comparison of Ethylene Biosynthesis and Signaling Differential Gene Expression Among Similar Tissues in Soybean Cultivars Under Drought Stress Conditions; **Figure S8**. Expression of Ethylene-Related Genes in Soybean Under Drought Stress Conditions; **Figure S9**. Evaluation of Physiological Parameters in Soybean Cultivars Under Drought Stress Conditions; **Figure S10**. Levels of Ethylene Production and Free ACC in Soybean Under Drought Stress Conditions. (PDF 3938 kb) Additional file 2:
**Protein Summarys and Best Bidirectional Hit (BBH) Results (Tables S1-S8). Table S1. ** Protein Summary and Best Bidirectional Hit (BBH) Results. *Arabidopsis thaliana* ethylene biosynthesis protein list; **Table S2.** *Arabidopsis thaliana* ethylene signal transduction protein list; **Table S3.** *Oryza sativa* ethylene biosynthesis protein list; **Table S4.** *Oryza sativa* ethylene signal transduction protein list; **Table S5.** Soybean ethylene biosynthesis protein summary; **Table S6.** Soybean ethylene signal transduction protein summary; **Table S7.** BBH Experiment - Soybean ethylene biosynthesis proteins; **Table S8.** BBH Experiment - Soybean proteins related with ethylene signal transduction. (PDF 1233 kb) Additional file 3:
***In silico*** **Characterization of Ethylene Soybean Genes.** Detailed description of characterization, gene localization and gene onthology (GO) of ethylene soybean genes. (PDF 12 kb) Additional file 4:
**Identification of** *cis* **-Acting Elements in Soybean Putative Gene Promoters (Table S9).** In this table are shown the *cis*-acting elements present in the putative promoters of 176 analyzed genes. The analysis matrix was composed by 100 different elements, distributed in 29 families. Cells highlighted in different colors corresponding to elements in each promoter sequence identified, associated with the number of the identified elements. Thus, sequences with green and red cells respectively represent putative gene promoters in ethylene biosynthesis and signal transduction mediated by this plant hormone. All results are presented in three ways: the total number of matches for each *cis*-acting element in the matrix analyzed (column E); the number of different sequences of putative promoters that represent each *cis*-acting element that compose the matrix (column F); and, at last, the number of different sequences of putative promoters that represent each *cis*-acting element family (column G). These values are totaled, and the total number of matches identified in each sequence analyzed (line 107). (******) N.A. corresponds to not applicable. (XLSX 84 kb) Additional file 5:
**Real Time PCR (RT-qPCR) Cycle Threshold (C**_t_ **) (Tables S10-S11). Table S10.** Target and endogenous gene cycle threshold in soybean leaf under drought stress – RT-qPCR; **Table S11.** Target and endogenous gene cycle threshold in soybean root under drought stress – RT-qPCR. (PDF 326 kb) Additional file 6:
**Real Time PCR (RT-qPCR) Primers (Method S1).** Gene summary and primers for Real Time PCR. (PDF 227 kb)

## Abbreviations

AAT: Amino acid transferase
ABA: Abscisic acid
ABR: ABA repressor
ABRE: ABA response elements
ACC: 1-aminocyclopropane-1-carboxylic acid
ACD: ACC deaminase
ACO (EFE): ACC oxidase (ethylene forming enzyme)
ACS: ACC synthase
ACT: Acyltransferase
AdoMet or SAM: S-adenosylmethionine
AP2: APETALA2
ARD: Acireductone dioxigenase
AREF: Auxin response elements
ASP: Aspartate aminotransferase
ATAF: ATAF-like NAC domain containing proteins
BBH: Best bidirectional hit
BRRE: Brassinosteroid response elements
CAAT: CCAAT binding factors
CBF: *C* repeat/interaction factor with DRE
CDC5: *A. thaliana* CDC5 homologs
CDPK (CPK): Calcium-dependent protein kinase
CE1F: Coupling elements 1 binding factors
ChIP: Chromatin immunoprecipitation
CNAC: Calcium regulated NAC-factors
CTR: Constitutive triple response
DEP: Dehydratase-enolase-phosphatase complex
DPBF: *Dc3* promoter binding factors
DRE: Drought response element
DREB: Drought response element binding protein
GACC: 1-glutamyl-ACC
EAR: Ethylene responsive element binding factor associated with amphiphilic repression
EBF: EIN3 binding F-box protein
EIL: EIN3-like
EIN: Ethylene insensitive
EINL: EIN3-like factors
EOL: ETO-like
EREBEP: Ethylene responsive element binding protein
EREF: Ethylene response element factors
ERF: Ethylene response factor
ETO: Ethylene overproducer
ETP: EIN2 targeting protein
ETR: Ethylene receptor
FLO2: Floral homeotic protein APETALA2
GARP: MYB-related DNA binding proteins - Golden2, ARR, Psr
GBOX: plant *G*-box/*C*-box bZIP proteins
GCCF: *GCC-*box family
GO: Gene ontology
GTT: γ-glutamyl transpeptidase
HEAT: Heat shock factors
JARE: Jasmonate response elements
LREM: Light responsive element motifs, not modulated by different light qualities
MACC: 1-malonyl-ACC
MIIG: MYB IIG-type binding sites
MKK: MAP kinase kinase
MKKK: MAP kinase kinase kinase
MPK: Mitogen-activated protein kinase
MTA: 5-methylthioadenosine
MTI: 5-methylthioribose-1-phosphate isomerase
MTK: 5-methylthioribose kinase
MTN: 5-methylthioadenosine nucleosidase
MYBL: MYB-like proteins
MYBS: MYB proteins with single DNA binding repeat
MYCL: MYC-like basic helix-loop-helix binding factors
NACF: Plant specific NAC transcriptional factors
PTBP: Plant *TATA* binding protein factors
SAMS (MAT): S-adenosylmethionine synthetase (methionine adenosyltransferase)
RAN: Responsive to agonist
RAV: Related to ABI3/VP1
RAV3: 3’-part of bipartite RAV1 binding site
RAV5: 5’-part of bipartite RAV1 binding site
RTE: Reversion to ethylene sensitivity
SALT: Salt/drought responsive elements
SSH: Subtraction library hybridization
SWNS: Secondary wall NACS
WUE: Water consumption
