# Supplementary material for: Implications of ethylene biosynthesis and signaling in soybean drought stress tolerance
Source: BMC Plant Biol. 2015 Sep 3;15:213. doi: 10.1186/s12870-015-0597-z (PMC4557918; doi:10.1186/s12870-015-0597-z)
Supplement: Additional file 1:Supplementary Figures (Figs. S1-S10). — Figure S1. Soybean Chromosomal Ideogram; Figure S2. Gene Ontology Classification; Figure S3. Protein Orthology by Best Bidirectional Hit (BBH) Analysis; Figure S4. ACSs Classification; Figure S5. Differential Expression of Genes Related to Soybean Ethylene Biosynthesis in Transcriptomes Under Drought Stress Conditions; Figure S6. Differential Expression of Genes Related to Soybean Ethylene Signal Transduction in Transcriptomes Under Drought Stress Conditions; Figure S7. Comparison of Ethylene Biosynthesis and Signaling Differential Gene Expression Among Similar Tissues in Soybean Cultivars Under Drought Stress Conditions; Figure S8. Expression of Ethylene-Related Genes in Soybean Under Drought Stress Conditions; Figure S9. Evaluation of Physiological Parameters in Soybean Cultivars Under Drought Stress Conditions; Figure S10. Levels of Ethylene Production and Free ACC in Soybean Under Drought Stress Conditions. (PDF 3938 kb) [file 12870_2015_597_MOESM1_ESM.pdf]

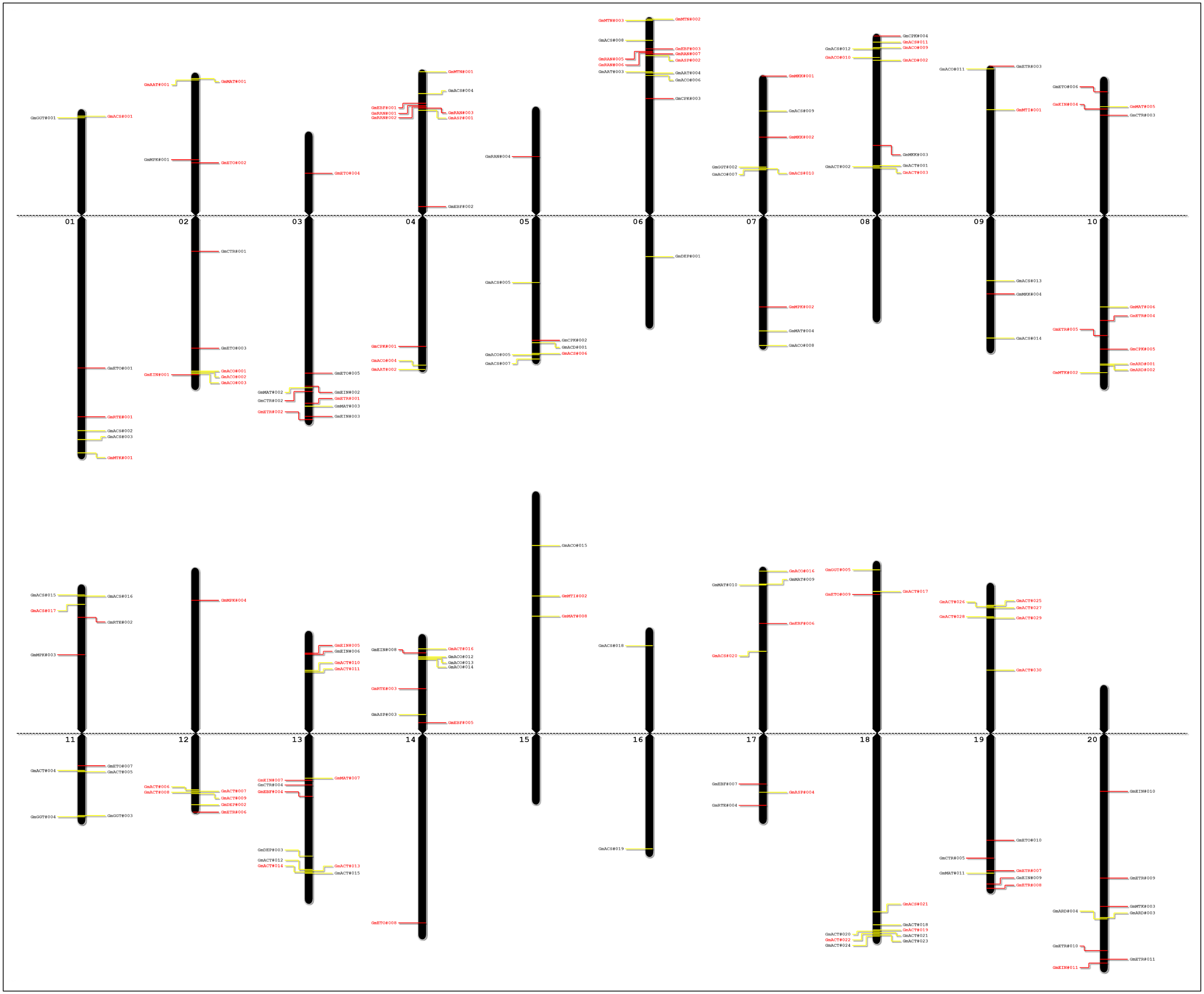

Figure S1. <u>Soybean Chromosomal Ideogram.</u> In figure, are represented the positions of 176 genes identified in 20 soybean chromosome. Each gene is represented by a generic name (see Tables S5 and S6), and the relative position in the chromosome is determined by the color code (names in **black** - plus strand; names in **red** - minus strand). The **yellow** and **red** lines represent respectively genes related to ethylene biosynthesis and signal transduction mediated by this phytohormone. The **dotted black lines** pass through the centromere midpoint in each chromosome. Scale: 1.0 cm equates to 5.0 Mb (megabase).

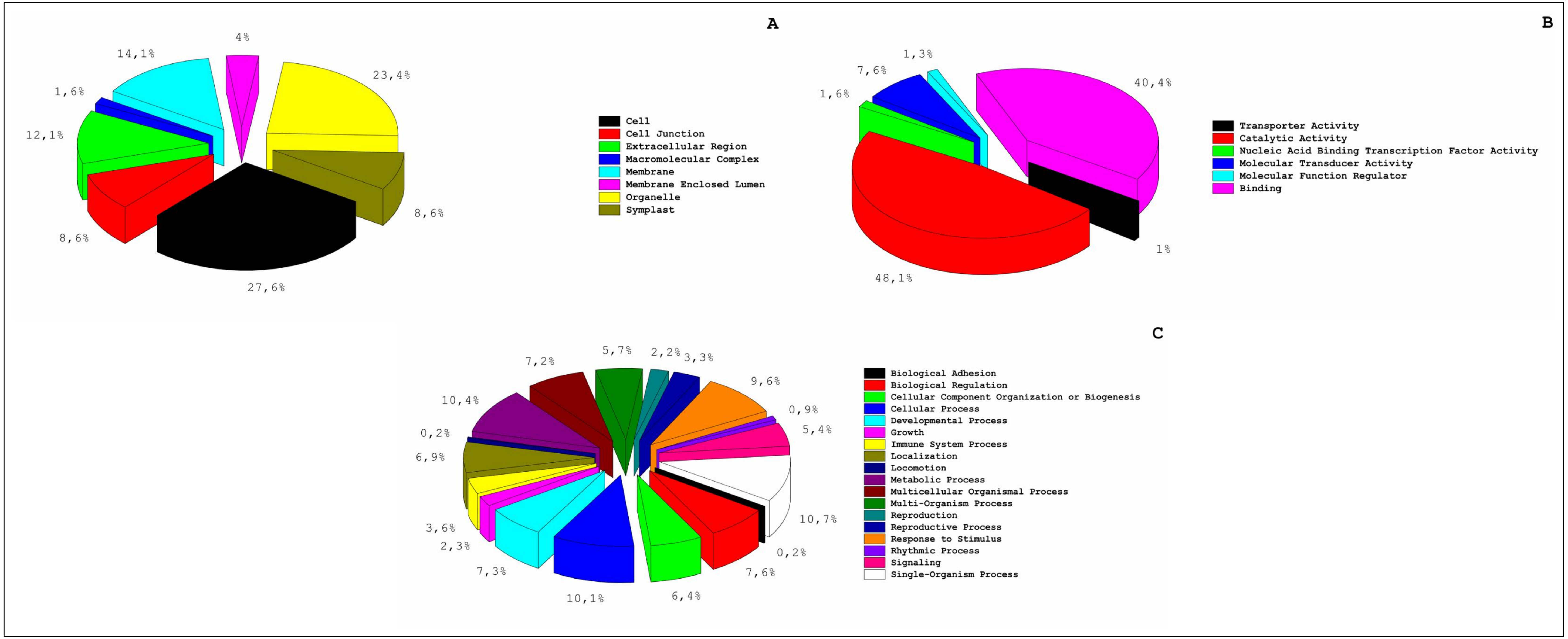

Figure S2. **Gene Ontology Classification.** The three graphs show the ontological subgroups (level 2) of the 176 soybean selected sequences. *Legends*: **A** - cellular component; **B** - molecular function; **C** - biological process.

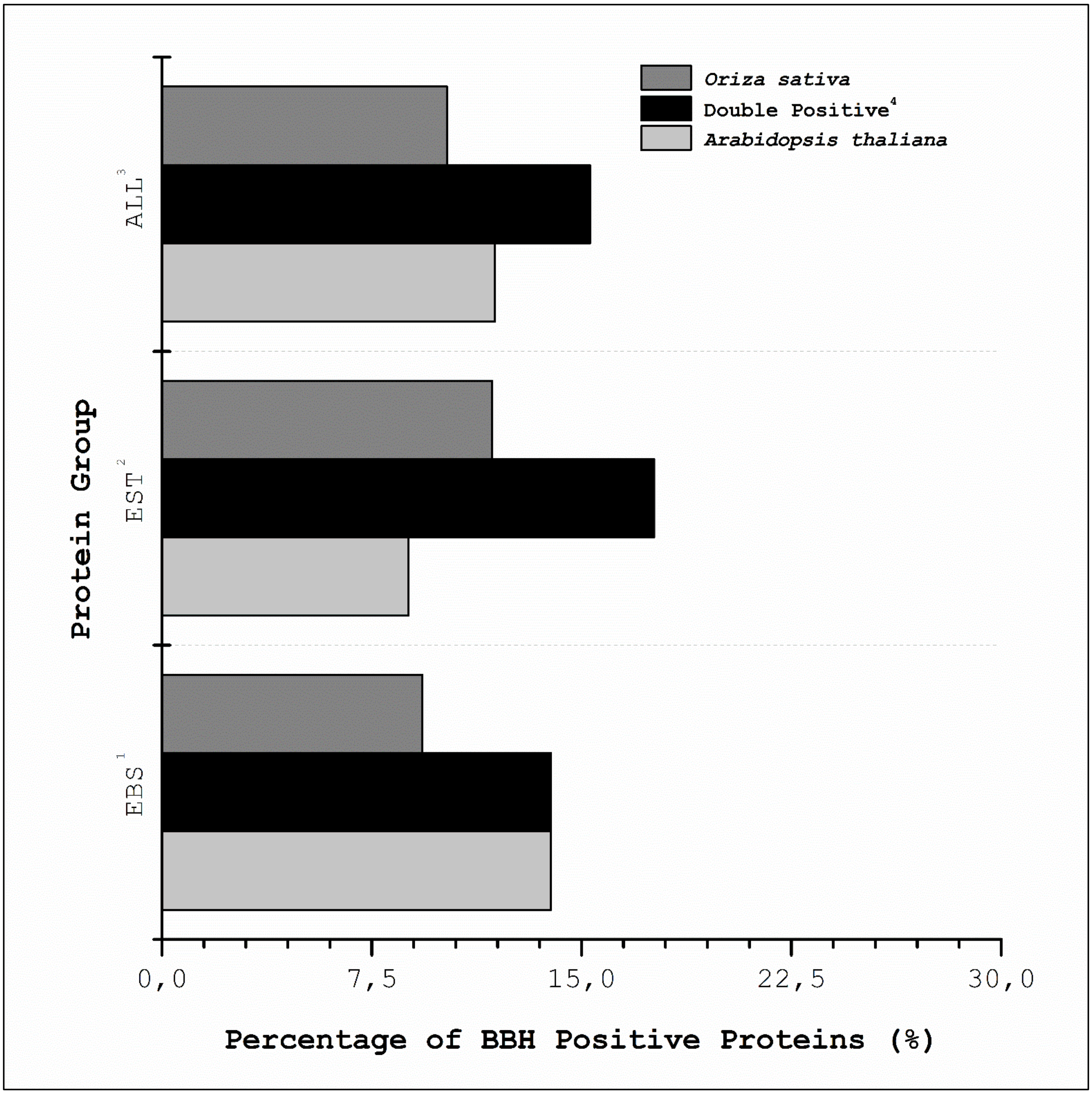

Figure S3. **Protein Orthology by Best Bidirectional Hit (BBH) Analysis.** Percentage of soybean BBH positive proteins present in each group analyzed. [1] **EBS** and [2] **EST** - ethylene biosynthesis and signal transduction proteins, respectively; [3] **ALL** - overall BBH positive percentage (considering all soybean proteins analyzed); [4] **Double Positive** - soybean proteins BBH positive with *A. thaliana* and *O. sativa* simultaneously. The proteins identified in this experiment are listed in Tables S7 and S8 (Additional File 2).

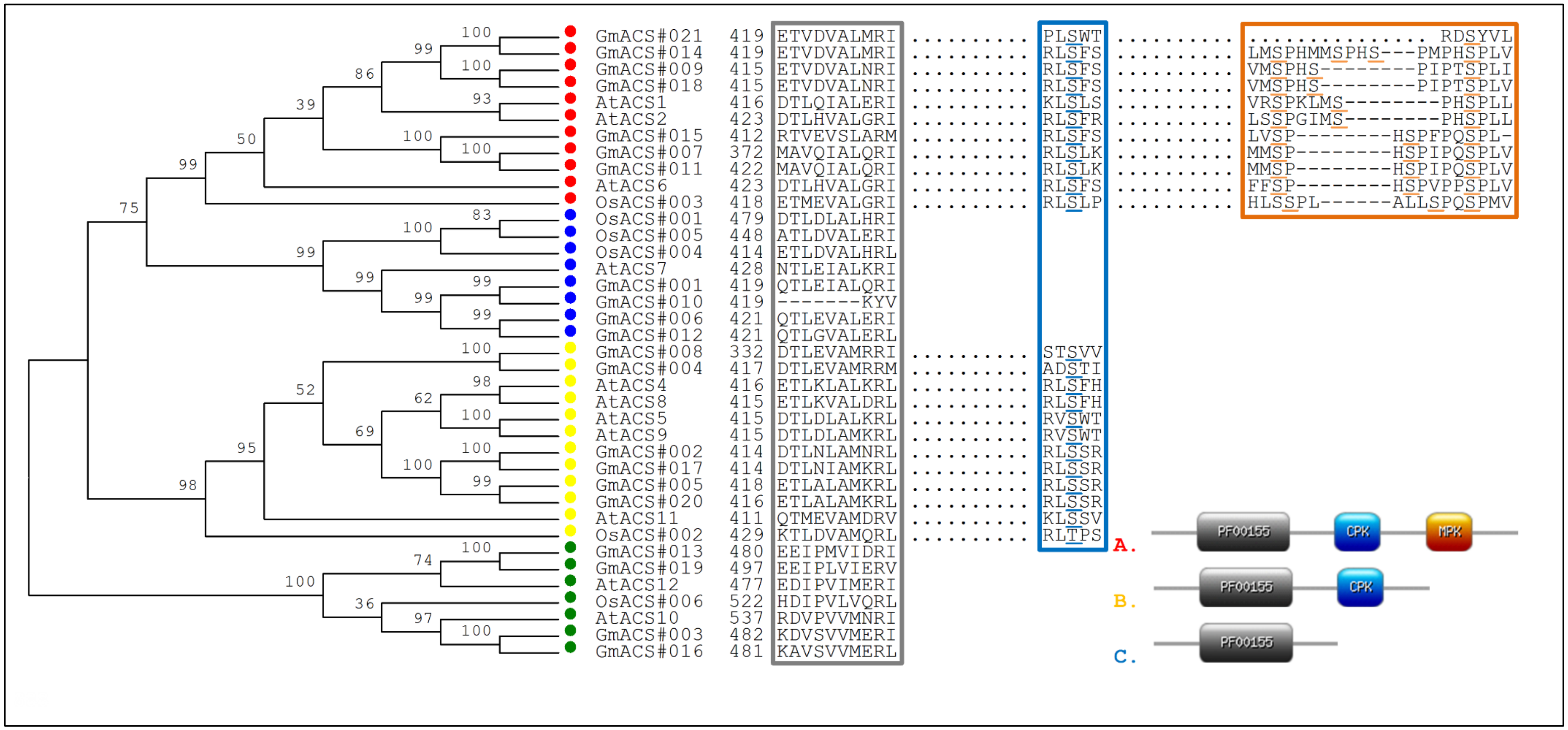

Figure S4. ACSs Classification. The figure shows the relationship among amino acid sequence of ACSs (1-aminocyclopropane-1-carboxilic acid synthase) identified in soybean, *Arabidopsis thaliana* and *Oryza sativa*. This relation allows the classification according to the presence/absence of potential sites of phosphorylation by calcium dependent protein kinase (CPK or CDPK) and/or MPK6 protein (mitogen-activated protein kinase 6 - MAPK6) in C-terminal of these proteins. Thus, these amino acid sequences can be divided into three classes: *type I* (**red circles**; **model A**) - proteins which exhibit extended C-terminal with conserved residues that are targets for phosphorylation by MPK6, as well as a conserved residue that is a phosphorylation site for CPK; *type II* (**yellow circles**; **model B**) - proteins which exhibit only CPK sites; and *type III* (**blue circles**; **model C**) – proteins that lacking both phosphorylation sites [18,55]. The proteins represented by **green circles** are classified as ACS-like since AtACS10 and AtACS12 possibly do not have ACS activity and are most probably amino acid transferases (AATs) [17]. AtACS1 also does not have ACS activity, by deletions in catalytic core, but AtACS2 does [17]. The **gray rectangle** highlights C-terminal of catalytic core (position at left), the **blue rectangle** the CPK phosphorylation sites and the **orange rectangle** the MPK6 sites. The **underlined amino acid residues** are the most likely to be phosphorylated in each sequence. Each protein is identified by generic name (see Tables S1, S3 and S5 in Additional File 2).

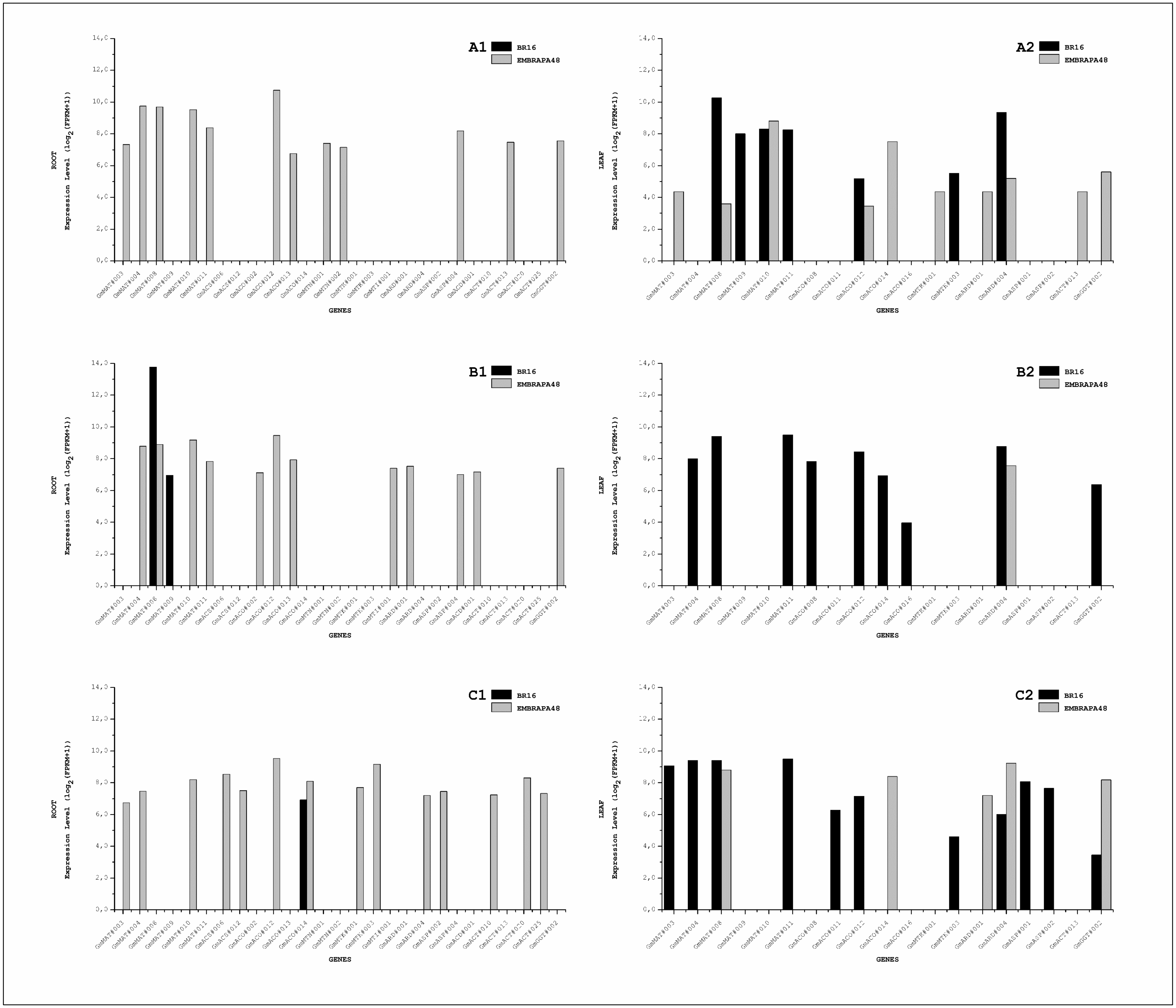

Figure S5. Differential Expression of Genes Related to Soybean Ethylene Biosynthesis in Transcriptomes Under Drought Stress Conditions. The graphics represent the expression levels of genes related to ethylene biosynthesis in root and leaf transcriptomes of two soybean cultivars: BR16 and EMBRAPA48, sensitive and tolerant to drought stress, respectively. Each gene is identified by generic name (see Table S5 in Additional File 2). The symbols correspond to: **A1** - root/25 at 50 minutes under drought conditions; **A2** - leaf/25 at 50 minutes under drought conditions; **B1** - root/75 at 100 minutes under drought conditions; **B2** - leaf/75 at 100 minutes under drought conditions; **C1** - root/125 at 150 minutes under drought conditions; **C2** - leaf/125 at 150 minutes under drought conditions; **FPKM** - fragments per kilobase of transcript per million fragments mapped.

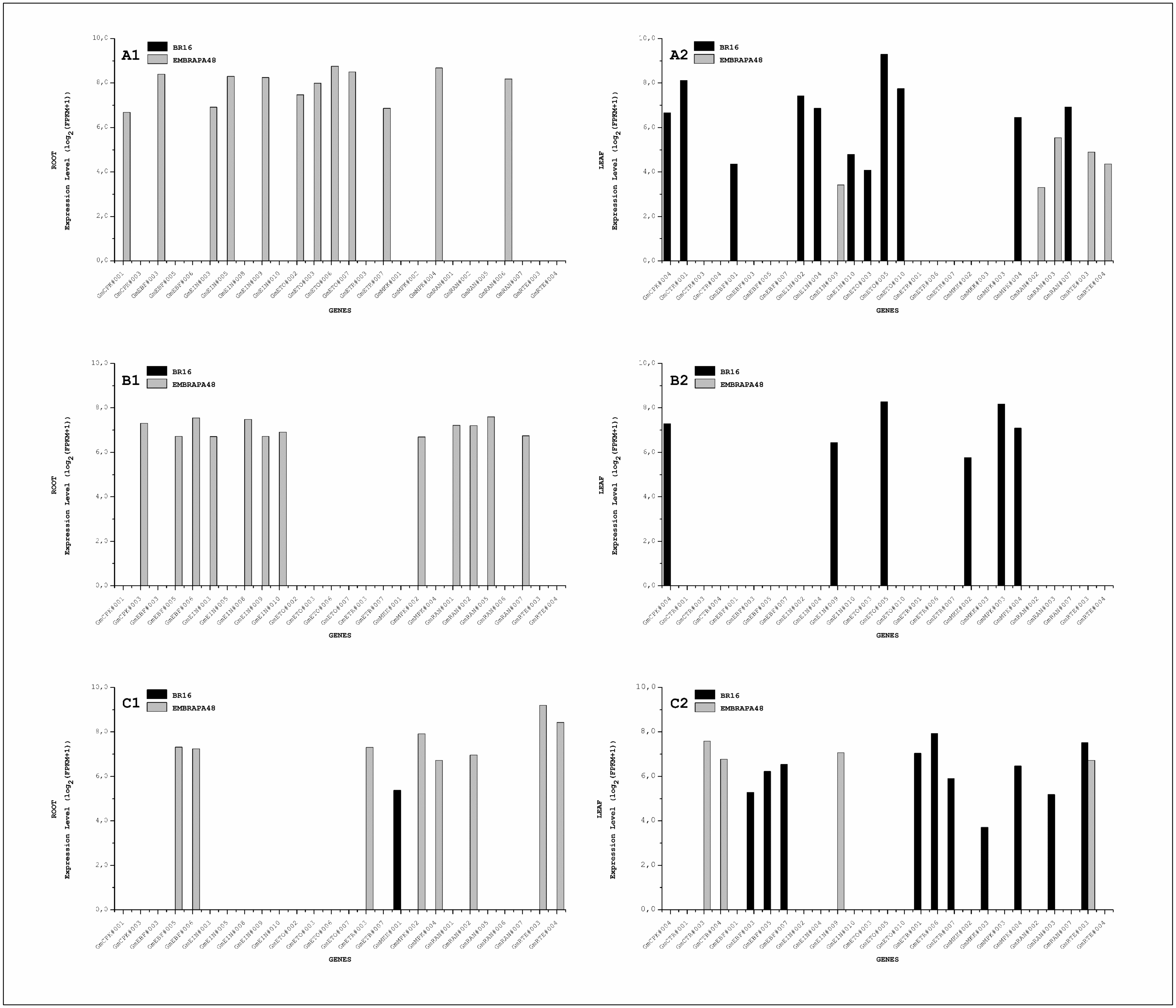

Figure S6. <u>Differential Expression of Genes Related to Soybean Ethylene Signal Transduction in Transcriptomes Under Drought Stress Conditions.</u> The graphics represent the expression levels of genes related to ethylene signal transduction in root and leaf transcriptomes of two soybean cultivars: BR16 and EMBRAPA48, sensitive and tolerant to drought stress, respectively. Each gene is identified by generic name (see Table S6 in Additional File 2). The symbols correspond to: **A1** - root/25 at 50 minutes under drought conditions; **A2** - leaf/25 at 50 minutes under drought conditions; **B1** - root/75 at 100 minutes under drought conditions; **B2** - leaf/75 at 100 minutes under drought conditions; **C1** - root/125 at 150 minutes under drought conditions; **C2** - leaf/125 at 150 minutes under drought conditions; **FPKM** - fragments per kilobase of transcript per million fragments mapped.

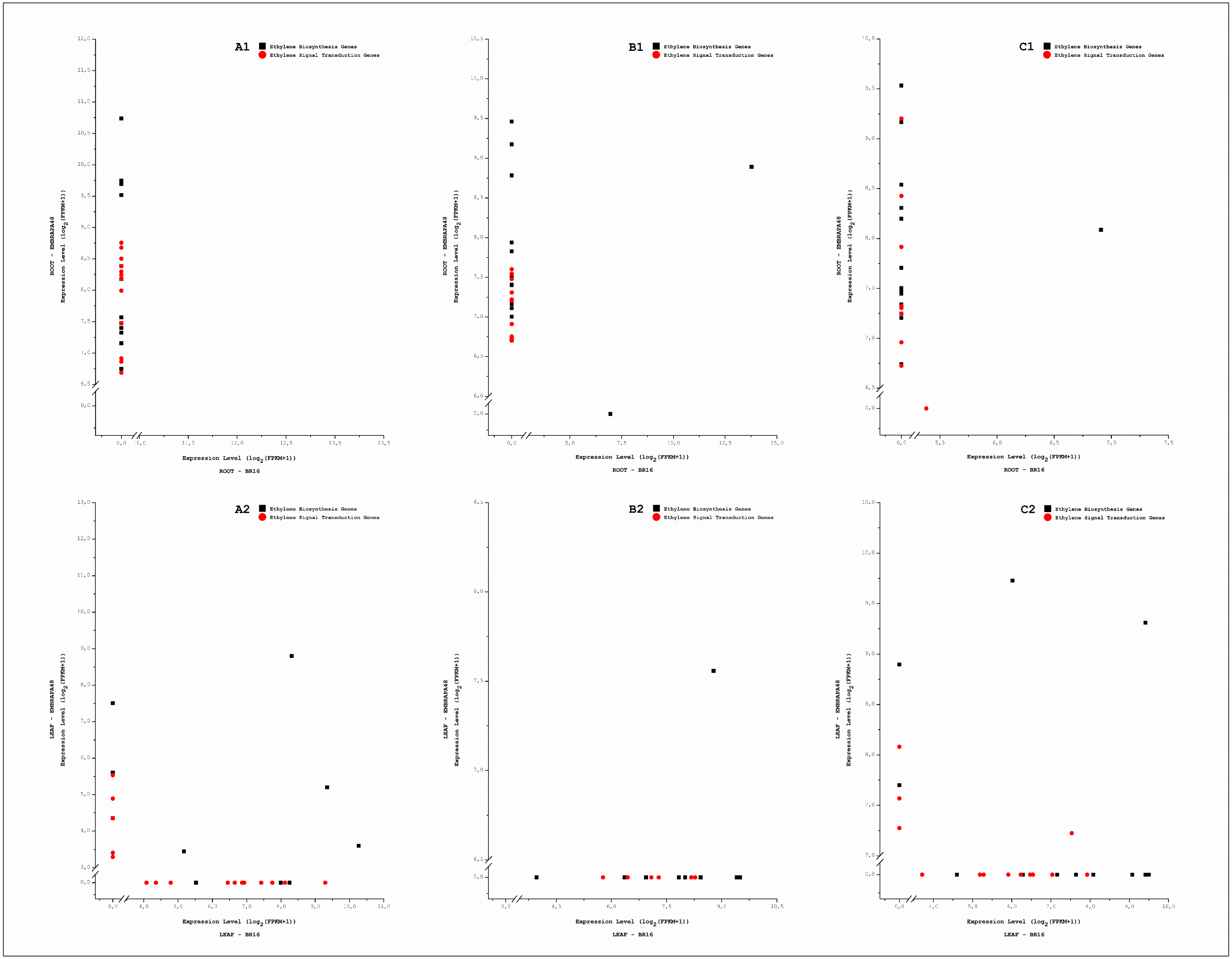

Figure S7. **Comparison of Ethylene Biosynthesis and Signaling Differential Gene Expression Among Similar Tissues in Soybean Cultivars Under Drought Stress Conditions.** The scatter plots compare the expression levels of ethylene biosynthesis and signal transduction genes among similar tissues of BR16 and EMBRAPA48 soybean cultivars, sensitive and tolerant to drought stress, respectively. Every expressed gene (see transcriptome data in Figures S5 and S6) is represented in each plot by one point whose coordinates correspond to expression levels in similar tissues of both cultivars. The symbols correspond to: **A1** - root/25 at 50 minutes under drought conditions; **A2** - leaf/25 at 50 minutes under drought conditions; **B1** - root/75 at 100 minutes under drought conditions; **B2** - leaf/75 at 100 minutes under drought conditions; **C1** - root/125 at 150 minutes under drought conditions; **C2** - leaf/125 at 150 minutes under drought conditions; **FPKM** - fragments per kilobase of transcript per million fragments mapped.

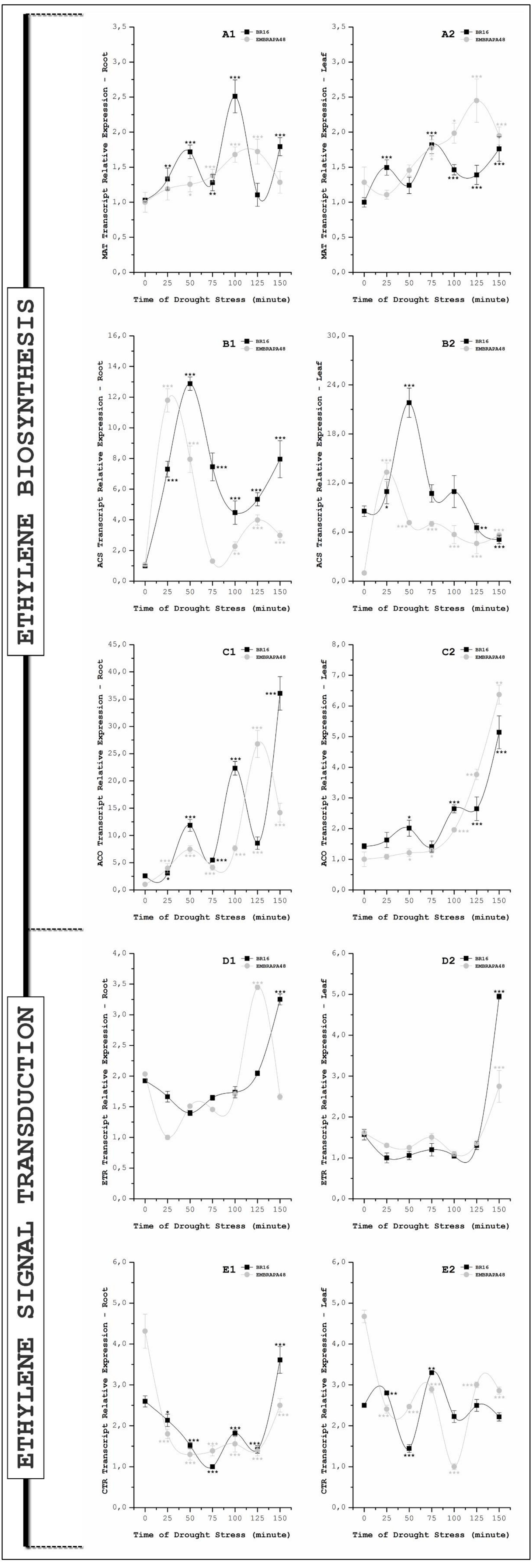

Figure S8. **Expression of Ethylene-Related Genes in Soybean Under Drought Stress Conditions.** The graphs show the expression levels, obtained by RT-qPCR, of five soybean genes related to ethylene biosynthesis [*MAT* (**A**), *ACS* (**B**) and *ACO* (**C**)] and ethylene signal transduction [*ETR* (**D**) and *CTR* (**E**)]. The expression of these genes in the experiment was compared in roots and leaves of soybean cultivars BR16 and EMBRAPA48 after different durations of drought stress. The statistics were obtained by comparing non-stressed plants (time zero) with stressed plants (at different times of drought stress). The asterisks represent the level of statistical significance: (*****) $p \leq 0.05$; (******) $0.01 \leq p < 0.05$; (*******) $0.001 \leq p < 0.01$. Each dot represents the average amount (± standard error) of three experimental replicates (same sample) in three biological samples (different plants), totaling nine replicates. The standard error is not presented with some of the dots because their absolute values are lower than the scale. After normalization based on housekeeping genes, the values given in the graph are relative to the lowest expression, whose value was set at 1 (one). Information about the target genes is presented in Method S1 (Additional File 6).

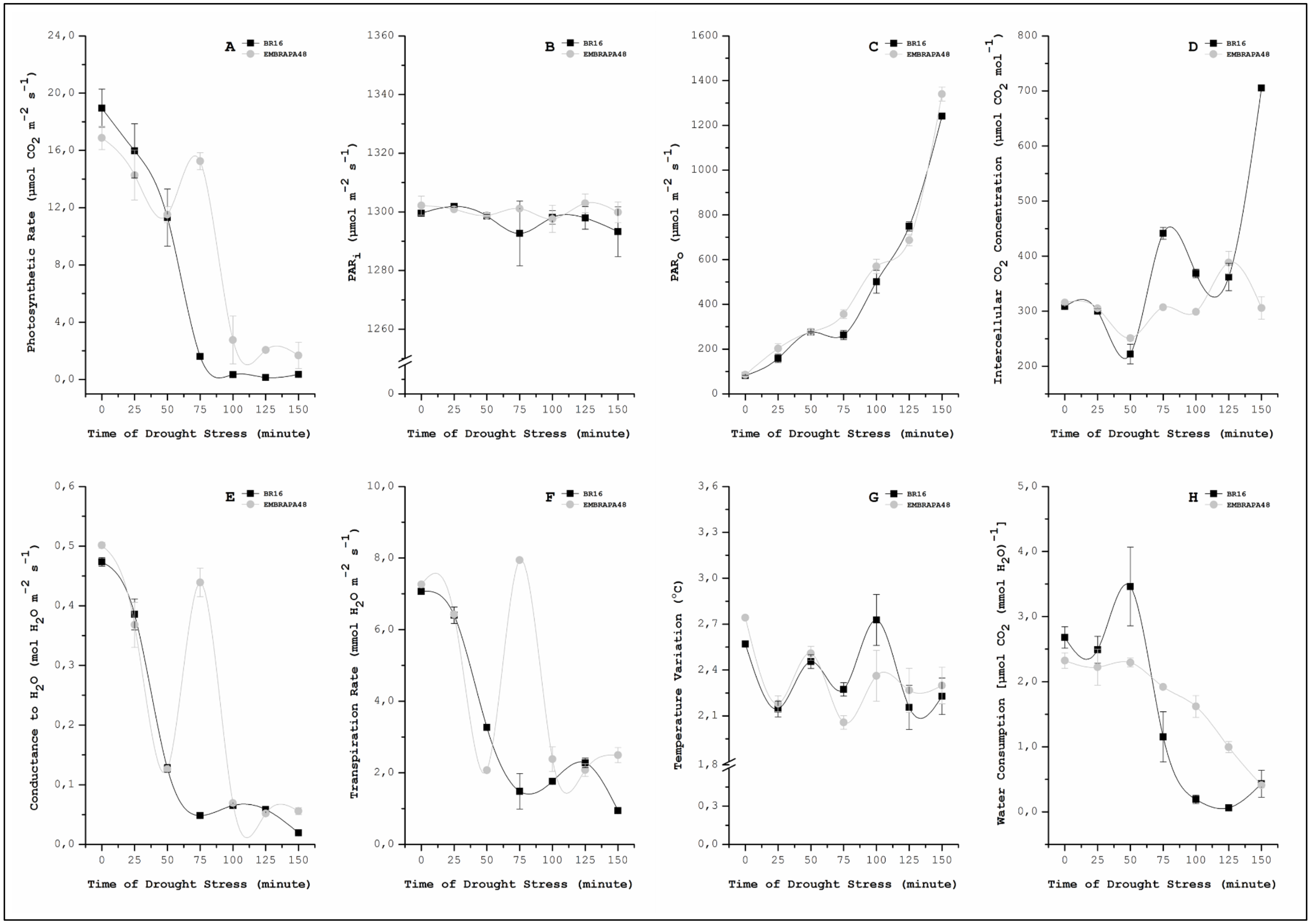

Figure S9. <u>Evaluation of Physiological Parameters in Soybean Cultivars Under Drought Stress Conditions.</u> During the drought stress experiments in BR16 and EMBRAPA48 soybean cultivars, grown under hydroponic conditions, were determined some relevant physiological parameters: **A** - photosynthetic rate (*A*); **B** - photosynthetically active radiation (internal to the reading chamber - PAR_i); **C** - photosynthetically active radiation (external to the reading chamber - PAR_o); **D** - intercellular CO_2 concentration (C_i); **E** - conductance to H_2O (or estomatic conductance - g_s); **F** - transpiration rate (*E*); **G** - temperature variation (ΔT), where [$\Delta T = T_{air}$ (internal to the reading chamber - T_{air}) – T_{leaf}; average air temperature = 29.7±1.9ºC]; **H** - water use efficiency (*WUE*, ratio among photosynthetic and transpiration rates - *A/E*). Each dot represents the average amount (± standard error) of three replicates in different plants. The absence of representation of standard error occurs in some dots by the fact of their absolute values are lower than the scale.

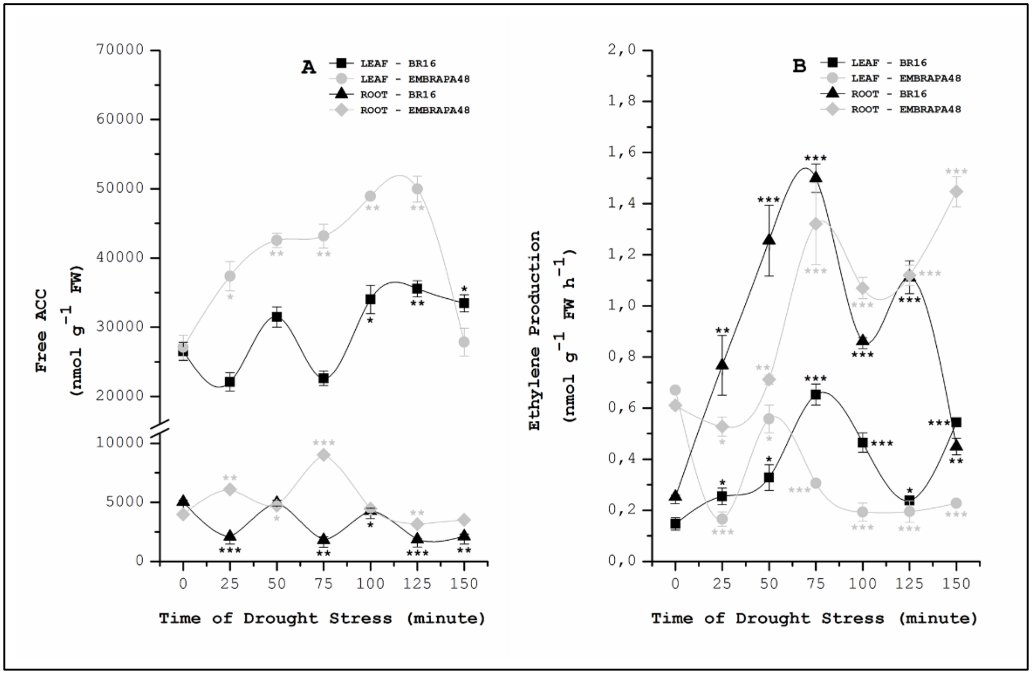

Figure S10. <u>Levels of Ethylene Production and Free ACC in Soybean Under Drought Stress Conditions.</u> Values were determined for ethylene production and free ACC (1-aminocyclopropane-1-carboxylic acid) in roots and leaves of soybean cultivars BR16 and EMBRAPA48 after the application of different durations of drought stress. The codes **A** represents levels of free ACC; **B** represents levels of ethylene production. The statistics were obtained by comparing non-stressed plants (time zero) with stressed plants (at different durations of drought stress). The asterisks represent the level of statistical significance: (*****) $p \leq 0.05$; (******) $0.05 < p \leq 0.01$; (*******) $0.01 < p \leq 0.001$. Each dot represents the average amount (± standard error) of three replicates in different plants. The standard error is not presented with some dots because their absolute values are lower than the scale.
