# Supplementary material for: Implications of ethylene biosynthesis and signaling in soybean drought stress tolerance
Source: BMC Plant Biol. 2015 Sep 3;15:213. doi: 10.1186/s12870-015-0597-z (PMC4557918; doi:10.1186/s12870-015-0597-z)
Supplement: Additional file 2: — Protein Summarys and Best Bidirectional Hit (BBH) Results (Tables S1-S8). Table S1. Protein Summary and Best Bidirectional Hit (BBH) Results. Arabidopsis thaliana ethylene biosynthesis protein list; Table S2. Arabidopsis thaliana ethylene signal transduction protein list; Table S3. Oryza sativa ethylene biosynthesis protein list; Table S4. Oryza sativa ethylene signal transduction protein list; Table S5. Soybean ethylene biosynthesis protein summary; Table S6. Soybean ethylene signal transduction protein summary; Table S7. BBH Experiment - Soybean ethylene biosynthesis proteins; Table S8. BBH Experiment - Soybean proteins related with ethylene signal transduction. (PDF 1233 kb) [file 12870_2015_597_MOESM2_ESM.pdf]

Table S1. *Arabidopsis thaliana* Ethylene Biosynthesis Protein List

Arabidopsis thaliana Protein			Protein Domain			Pathway
Generic Name	Locus ID	Size[1]	PFAM Code	Description	Position	
AtAAT	At1g77670	440	PF00155	Aminotransferase class I and II	079 - 432	Yang Cycle
AtACD1	At1g48420	401	PF00291	Pyridoxal-phosphate dependent enzyme	054 - 363	ACC Degradation
AtACO1	At2g19590	310	PF03171	2OG-Fe(II) oxygenase superfamily	158 - 259	Ethylene Biosynthesis
AtACO2	At1g62380	320	PF03171	2OG-Fe(II) oxygenase superfamily	156 - 256	Ethylene Biosynthesis
AtACO4	At1g05010	323	PF03171	2OG-Fe(II) oxygenase superfamily	153 - 254	Ethylene Biosynthesis
AtACO#001	At1g12010	320	PF03171	2OG-Fe(II) oxygenase superfamily	156 - 256	Ethylene Biosynthesis
AtACO#002	At1g77330	307	PF03171	2OG-Fe(II) oxygenase superfamily	156 - 256	Ethylene Biosynthesis
AtACS1	At3g61510	488	PF00155	Aminotransferase class I and II	047 - 425	Ethylene Biosynthesis
AtACS2	At1g01480	496	PF00155	Aminotransferase class I and II	048 - 432	Ethylene Biosynthesis
AtACS4	At2g22810	474	PF00155	Aminotransferase class I and II	040 - 425	Ethylene Biosynthesis
AtACS5	At5g65800	470	PF00155	Aminotransferase class I and II	040 - 424	Ethylene Biosynthesis
AtACS6	At4g11280	495	PF00155	Aminotransferase class I and II	051 - 432	Ethylene Biosynthesis
AtACS7	At4g26200	447	PF00155	Aminotransferase class I and II	054 - 437	Ethylene Biosynthesis
AtACS8	At4g37770	469	PF00155	Aminotransferase class I and II	040 - 424	Ethylene Biosynthesis
AtACS9	At3g49700	470	PF00155	Aminotransferase class I and II	040 - 424	Ethylene Biosynthesis
AtACS10	At1g62960	557	PF00155	Aminotransferase class I and II	169 - 546	Ethylene Biosynthesis[2]
AtACS11	At4g08040	460	PF00155	Aminotransferase class I and II	038 - 419	Ethylene Biosynthesis
AtACS12	At5g51690	495	PF00155	Aminotransferase class I and II	105 - 486	Ethylene Biosynthesis[2]
AtACT#001	At1g03940	469	PF02458	Transferase family	003 - 463	MACC Synthesis[3]
AtACT#002	At1g03495	465	PF02458	Transferase family	006 - 459	MACC Synthesis[3]
AtACT#003	At2g30200	393	PF02458	Transferase family	091 - 390	MACC Synthesis[3]
AtACT#004	At3g29670	451	PF02458	Transferase family	001 - 448	MACC Synthesis[3]
AtACT#005	At5g39050	469	PF02458	Transferase family	008 - 466	MACC Synthesis[3]
AtACT#006	At5g39090	448	PF02458	Transferase family	003 - 443	MACC Synthesis[3]
AtARD1	At4g14716	192	PF03079	ARD/ARD' family	014 - 168	Yang Cycle
			PF07883	Cupin domain	086 - 162	
AtARD2	At4g14710	199	PF03079	ARD/ARD' family	014 - 168	Yang Cycle
			PF07883	Cupin domain	086 - 162	
AtARD3	At2g26400	199	PF03079	ARD/ARD' family	014 - 168	Yang Cycle
			PF07883	Cupin domain	086 - 162	

Table S1. *Arabidopsis thaliana* Ethylene Biosynthesis Protein List (cont.)

Arabidopsis thaliana Protein			Protein Domain			Pathway
Generic Name	Locus ID	Size[1]	PFAM Code	Description	Position	
AtARD4	At5g43850	187	PF03079	ARD/ARD' family	004 - 158	Yang Cycle
			PF07883	Cupin domain	078 - 152	
AtASP2	At5g19550	405	PF00155	Aminotransferase class I and II	029 - 397	Yang Cycle
ATASP3	At5g11520	449	PF00155	Aminotransferase class I and II	073 - 441	Yang Cycle
AtASP5	At4g31990	453	PF00155	Aminotransferase class I and II	077 - 445	Yang Cycle
AtDEP1	At5g53850	507	PF00596	Class II aldolase and adducin N-terminal domain	026 - 229	Yang Cycle
			PF00702	Haloacid dehalogenase-like hydrolase	265 - 478	
AtGGT1	At4g39640	572	PF01019	Gamma-glutamyltranspeptidase	048 - 567	GACC Synthesis[3]
AtGGT2	At4g39650	578	PF01019	Gamma-glutamyltranspeptidase	052 - 573	GACC Synthesis[3]
AtGGT3	At1g69820	191	PF01019	Gamma-glutamyltranspeptidase	001 - 191	GACC Synthesis[3]
AtGGT4	At4g29210	637	PF01019	Gamma-glutamyltranspeptidase	095 - 632	GACC Synthesis[3]
AtMAT1	At1g02500	393	PF00438	S-adenosylmethionine synthetase, N-terminal domain	002 - 101	Ethylene Biosynthesis
			PF02772	S-adenosylmethionine synthetase, central domain	116 - 238	
			PF02773	S-adenosylmethionine synthetase, C-terminal domain	240 - 382	
AtMAT2	At4g01850	393	PF00438	S-adenosylmethionine synthetase, N-terminal domain	002 - 101	Ethylene Biosynthesis
			PF02772	S-adenosylmethionine synthetase, central domain	116 - 238	
			PF02773	S-adenosylmethionine synthetase, C-terminal domain	240 - 382	
AtMAT3	At2g36880	390	PF00438	S-adenosylmethionine synthetase, N-terminal domain	002 - 101	Ethylene Biosynthesis
			PF02772	S-adenosylmethionine synthetase, central domain	116 - 238	
			PF02773	S-adenosylmethionine synthetase, C-terminal domain	240 - 382	
AtMAT4	At3g17390	393	PF00438	S-adenosylmethionine synthetase, N-terminal domain	002 - 101	Ethylene Biosynthesis
			PF02772	S-adenosylmethionine synthetase, central domain	116 - 238	
			PF02773	S-adenosylmethionine synthetase, C-terminal domain	240 - 382	
AtMTI1	At2g05830	374	PF01008	Initiation factor 2 subunit family	050 - 353	Yang Cycle
AtMTK1	At1g49820	420	PF01636	Phosphotransferase enzyme family	038 - 295	Yang Cycle
AtMTN1	At4g38800	267	PF01048	Phosphorylase superfamily	028 - 257	Yang Cycle
AtMTN2	At4g34840	254	PF01048	Phosphorylase superfamily	015 - 244	Yang Cycle

[1] Amino acid residues.
[2] Putative amino acid transferase (AAT). No ACC synthase activity demonstrated.
[3] The primary function of this protein is not initially associated with this metabolic pathway.

Table S2. *Arabidopsis thaliana* Ethylene Signal Transduction Protein List

Arabidopsis thaliana Protein			Protein Domain			Function
Generic Name	Locus ID	Size[1]	PFAM Code	Description	Position	
AtCDPK2	At1g35670	495	PF00069	Protein kinase domain	028 - 284	ACS Type II Activation
			PF00036	EF hand	331 - 392	
					398 - 462	
AtCTR1	At5g03730	821	PF14381	Ethylene responsive protein kinase Le-CTR1	205 - 414	Negative Regulator of Ethylene Response
			PF07714	Protein tyrosine kinase	551 - 805	
AtEBF1	At2g25490	628	PF00646	F-box domain	064 - 104	Directs EIN3 to Proteolysis
AtEBF2	At5g25350	625	PF00646	F-box domain	054 - 095	Directs EIN3 to Proteolysis
AtEIL1	At2g27050	584	PF04873	Ethylene insensitive 3	049 - 431	Nuclear Transcription Factor
AtEIL2	At5g21120	518	PF04873	Ethylene insensitive 3	048 - 408	Nuclear Transcription Factor
AtEIN2	At5g03280	1294	PF01566	Natural resistance-associated macrophage protein	038 - 390	Acts Downstream of CTR1
AtEIN3	At3g20770	628	PF04873	Ethylene insensitive 3	049 - 422	Nuclear Transcription Factor
AtEIN5	At1g54490	947	PF03159	XRN 5'-3' exonuclease N-terminus	001 - 256	EBF RNA Silencing
			PF00098	Zinc knuckle	263 - 280	
AtEOL1	At4g02680	888	PF00515	Tetratricopeptide repeat	519 - 544	ACS Type II Negative Regulator
AtEOL2	At5g58550	925	PF00515	Tetratricopeptide repeat	738 - 771	ACS Type II Negative Regulator
AtETO1	At3g51770	959	PF13414	TPR repeat	875 - 940	ACS Type II Negative Regulator
AtEIN4	At3g04580	766	PF01590	GAF domain	181 - 331	Ethylene Receptor
			PF00512	Histidine kinase A (phosphoacceptor) domain	367 - 432	
			PF00072	Response regulator receiver domain	644 - 758	
AtERS1	At2g40940	613	PF01590	GAF domain	158 - 307	Ethylene Receptor
			PF00512	Histidine kinase A (phosphoacceptor) domain	343 - 408	
			PF02518	Histidine kinase-, DNA gyrase B- and HSP90-like ATPase	455 - 586	
AtERS2	At1g04310	645	PF01590	GAF domain	190 - 346	Ethylene Receptor
AtETP1	At3g18980	415	PF00646	F-box domain	001 - 043	Directs EIN2 to Proteolysis
			PF07734	F-box associated	214 - 398	
AtETP2	At3g18910	388	PF00646	F-box domain	007 - 044	Directs EIN2 to Proteolysis
			PF07734	F-box associated	206 - 373	
AtETR1	At1g66340	738	PF01590	GAF domain	158 - 307	Ethylene Receptor
			PF00512	Histidine kinase A (phosphoacceptor) domain	343 - 408	
			PF02518	Histidine kinase-, DNA gyrase B- and HSP90-like ATPase	455 - 582	
			PF00072	Response regulator receiver domain	612 - 726	

Table S2. *Arabidopsis thaliana* Ethylene Signal Transduction Protein List (cont.)

Arabidopsis thaliana Protein			Protein Domain			Function
Generic Name	Locus ID	Size[1]	PFAM Code	Description	Position	
AtETR2	At3g23150	773	PF01590	GAF domain	187 - 331	Ethylene Receptor
			PF00512	Histidine kinase A (phosphoacceptor) domain	367 - 427	
			PF00072	Response regulator receiver domain	648 - 763	
AtMKK4	At1g51660	366	PF00069	Protein kinase domain	079 - 334	ACS Type I Activation
AtMKK5	At3g21220	348	PF00069	Protein kinase domain	070 - 325	ACS Type I Activation
AtMKK9	At1g73500	310	PF00069	Protein kinase domain	047 - 306	EIN3 Activation
AtMPK3	At3g45640	370	PF00069	Protein kinase domain	041 - 324	EIN3 Activation
AtMPK6	At2g43790	395	PF00069	Protein kinase domain	065 - 348	ACS Type I/EIN3 Activation
AtRAN1	At5g20010	221	PF00071	Ras family	015 - 173	Copper Transporter
AtRAN2	At5g20020	221	PF00071	Ras family	015 - 173	Copper Transporter
AtRAN3	At5g55190	221	PF00071	Ras family	015 - 173	Copper Transporter
AtRAN4	At5g55080	222	PF00071	Ras family	015 - 173	Copper Transporter
AtRTE1	At2g26070	250	PF05608	Protein of unknown function (DUF778)	060 - 195	Positive Regulator of Ethylene Receptor
AtRTH1	At3g51040	231	PF05608	Protein of unknown function (DUF778)	038 - 175	Positive Regulator of Ethylene Receptor

[1] Amino acid residues.

Table S3. *Oryza sativa* Ethylene Biosynthesis Protein List

Oryza sativa Protein			Protein Domain			Pathway[2]
Generic Name	Locus ID	Size[1]	PFAM Code	Description	Position	
OsAAT#001	Os09g28050	466	PF00155	Aminotransferase class I and II	109 - 458	Yang Cycle
OsAAT#002	Os10g34350	412	PF00155	Aminotransferase class I and II	030 - 381	Yang Cycle
OsACD#001	Os02g53330	565	PF00291	Pyridoxal-phosphate dependent enzyme	082 - 388	ACC Degradation
OsACO#001	Os01g39860	312	PF03171	2OG-Fe(II) oxygenase superfamily	169 - 264	Ethylene Biosynthesis
OsACO#002	Os02g53180	344	PF03171	2OG-Fe(II) oxygenase superfamily	184 - 280	Ethylene Biosynthesis
OsACO#003	Os05g05680	308	PF03171	2OG-Fe(II) oxygenase superfamily	163 - 259	Ethylene Biosynthesis
OsACO#004	Os06g37590	293	PF03171	2OG-Fe(II) oxygenase superfamily	150 - 246	Ethylene Biosynthesis
OsACO#005	Os09g27750	322	PF03171	2OG-Fe(II) oxygenase superfamily	164 - 259	Ethylene Biosynthesis
OsACO#006	Os09g27820	322	PF03171	2OG-Fe(II) oxygenase superfamily	164 - 259	Ethylene Biosynthesis
OsACO#007	Os11g08380	309	PF03171	2OG-Fe(II) oxygenase superfamily	163 - 262	Ethylene Biosynthesis
OsACS#001	Os01g09700	510	PF00155	Aminotransferase class I and II	104 - 488	Ethylene Biosynthesis
OsACS#002	Os03g51740	487	PF00155	Aminotransferase class I and II	051 - 438	Ethylene Biosynthesis
OsACS#003	Os04g48850	483	PF00155	Aminotransferase class I and II	046 - 427	Ethylene Biosynthesis
OsACS#004	Os05g10780	437	PF00155	Aminotransferase class I and II	035 - 423	Ethylene Biosynthesis
OsACS#005	Os05g25490	496	PF00155	Aminotransferase class I and II	056 - 457	Ethylene Biosynthesis
OsACS#006	Os06g03990	542	PF00155	Aminotransferase class I and II	147 - 531	Ethylene Biosynthesis[3]
OsACT#001	Os02g57480	453	PF02458	Transferase family	006 - 444	MACC Synthesis[4]
OsACT#002	Os03g18590	387	PF02458	Transferase family	086 - 357	MACC Synthesis[4]
OsACT#003	Os07g23150	525	PF02458	Transferase family	067 - 510	MACC Synthesis[4]
OsARD#001	Os03g06620	198	PF03079	ARD/ARD' family	015 - 168	Yang Cycle
			PF07883	Cupin domain	097 - 153	
OsARD#002	Os04g27830	284	PF03079	ARD/ARD' family	079 - 247	Yang Cycle
			PF07883	Cupin domain	164 - 219	
OsARD#003	Os10g28350	199	PF03079	ARD/ARD' family	015 - 168	Yang Cycle
			PF07883	Cupin domain	096 - 152	
OsARD#004	Os10g28360	184	PF03079	ARD/ARD' family	015 - 155	Yang Cycle
			PF07883	Cupin domain	085 - 143	
OsASP#001	Os01g55540	460	PF00155	Aminotransferase class I and II	086 - 452	Yang Cycle
OsASP#002	Os02g14110	432	PF00155	Aminotransferase class I and II	060 - 425	Yang Cycle
OsASP#003	Os02g55420	458	PF00155	Aminotransferase class I and II	084 - 450	Yang Cycle
OsASP#004	Os06g35540	463	PF00155	Aminotransferase class I and II	091 - 456	Yang Cycle

Table S3. *Oryza sativa* Ethylene Biosynthesis Protein List (cont.)

Oryza sativa Protein			Protein Domain			Pathway[2]
Generic Name	Locus ID	Size[1]	PFAM Code	Description	Position	
OsDEP#001	Os11g29370	518	PF00596	Class II aldolase and adducin N-terminal domain	032 - 232	Yang Cycle
			PF00702	Haloacid dehalogenase-like hydrolase	279 - 494	
OsGGT#001	Os01g05810	634	PF01019	Gamma-glutamyltranspeptidase	098 - 628	GACC Synthesis[4]
OsGGT#002	Os01g05820	563	PF01019	Gamma-glutamyltranspeptidase	094 - 457	GACC Synthesis[4]
OsGGT#003	Os04g38450	574	PF01019	Gamma-glutamyltranspeptidase	057 - 569	GACC Synthesis[4]
OsMAT#001	Os01g18860	396	PF00438	S-adenosylmethionine synthetase, N-terminal domain	006 - 104	Ethylene Biosynthesis
			PF02772	S-adenosylmethionine synthetase, central domain	119 - 241	
			PF02773	S-adenosylmethionine synthetase, C-terminal domain	243 - 384	
OsMAT#002	Os01g22010	394	PF00438	S-adenosylmethionine synthetase, N-terminal domain	005 - 103	Ethylene Biosynthesis
			PF02772	S-adenosylmethionine synthetase, central domain	118 - 240	
			PF02773	S-adenosylmethionine synthetase, C-terminal domain	242 - 382	
OsMAT#003	Os05g04510	396	PF00438	S-adenosylmethionine synthetase, N-terminal domain	006 - 104	Ethylene Biosynthesis
			PF02772	S-adenosylmethionine synthetase, central domain	119 - 241	
			PF02773	S-adenosylmethionine synthetase, C-terminal domain	243 - 384	
OsMTI#001	Os11g11050	508	PF01008	Initiation factor 2 subunit family	183 - 484	Yang Cycle
OsMTK#001	Os04g57400	430	PF01636	Phosphotransferase enzyme family	047 - 297	Yang Cycle
OsMTK#002	Os04g57410	427	PF01636	Phosphotransferase enzyme family	044 - 294	Yang Cycle
OsMTN#001	Os06g02220	259	PF01048	Phosphorylase superfamily	020 - 248	Yang Cycle

[1] Amino acid residues.
[2] This parameter was determinated by homology with equivalent *Arabidopsis thaliana* amino acid sequence.
[3] Putative amino acid transferase (AAT). No ACC synthase activity demonstrated.
[4] The primary function of this protein is not initially associated with this metabolic pathway.

Table S4. *Oryza sativa* Ethylene Signal Transduction Protein List

Oryza sativa Protein			Protein Domain			Putative Function[2]
Generic Name	Locus ID	Size[1]	PFAM Code	Description	Position	
OsCPK#001	Os11g07040	513	PF00069	Protein kinase domain	047 - 305	ACS Type II Activation
			PF00036	EF hand	352 - 413	
					418 - 483	
OsCPK#002	Os12g07230	526	PF00069	Protein kinase domain	049 - 307	ACS Type II Activation
			PF00036	EF hand	354 - 415	
					420 - 485	
OsCTR#001	Os02g32610	781	PF07714	Protein tyrosine kinase	167 - 377	Negative Regulator of Ethylene Response
			PF14381	Ethylene responsive protein kinase Le-CTR1	512 - 766	
OsCTR#002	Os09g39320	1220	PF07714	Protein tyrosine kinase	121 - 329	Negative Regulator of Ethylene Response
			PF14381	Ethylene responsive protein kinase Le-CTR1	481 - 723	
OsEBF#001	Os02g10700	660	PF00646	F-box domain	066 - 111	Directs EIN3 to Proteolysis
OsEBF#002	Os06g40360	664	PF00646	F-box domain	077 - 113	Directs EIN3 to Proteolysis
OsEIN#001	Os02g28074	822	PF03159	XRN 5'-3' exonuclease N-terminus	001 - 230	EBF RNA Silencing
OsEIN#002	Os03g20780	643	PF04873	Ethylene insensitive 3	078 - 464	Nuclear Transcription Factor
OsEIN#003	Os03g20790	640	PF04873	Ethylene insensitive 3	078 - 464	Nuclear Transcription Factor
OsEIN#004	Os03g49400	1299	PF01566	Natural resistance-associated macrophage protein	039 - 391	Acts Downstream of CTR1
OsEIN#005	Os03g58060	988	PF03159	XRN 5'-3' exonuclease N-terminus	001 - 256	EBF RNA Silencing
			PF00098	Zinc knuckle	263 - 280	
OsEIN#006	Os07g06130	1281	PF01566	Natural resistance-associated macrophage protein	047 - 399	Acts Downstream of CTR1
OsEIN#007	Os07g06190	1367	PF01566	Natural resistance-associated macrophage protein	258 - 584	Acts Downstream of CTR1
OsEIN#008	Os07g06300	1367	PF01566	Natural resistance-associated macrophage protein	258 - 584	Acts Downstream of CTR1
OsETO#001	Os03g18360	958	PF07719	Tetratricopeptide repeat	704 - 737	ACS Type II Negative Regulator
OsETO#002	Os07g08120	886	PF13414	TPR Repeat	707 - 772	ACS Type II Negative Regulator
OsETO#003	Os11g37520	886	PF13414	TPR Repeat	804 - 869	ACS Type II Negative Regulator
OsETR#001	Os02g57530	836	PF01590	GAF domain	269 - 413	Ethylene Receptor
			PF00512	Histidine kinase A (phosphoacceptor) domain	449 - 514	
			PF00072	Response regulator receiver domain	717 - 830	
OsETR#002	Os03g49500	636	PF01590	GAF domain	158 - 307	Ethylene Receptor
			PF00512	Histidine kinase A (phosphoacceptor) domain	343 - 408	
			PF02518	Histidine kinase-, DNA gyrase B- and HSP90-like ATPase	455 - 586	

Table S4. *Oryza sativa* Ethylene Signal Transduction Protein List (cont.)

Oryza sativa Protein			Protein Domain			Putative Function[2]
Generic Name	Locus ID	Size[1]	PFAM Code	Description	Position	
OsETR#003	Os04g08740	763	PF01590	GAF domain	190 - 339	Ethylene Receptor
			PF00072	Response regulator receiver domain	642 - 757	
OsETR#004	Os05g06320	518	PF01590	GAF domain	159 - 308	Ethylene Receptor
			PF00512	Histidine kinase A (phosphoacceptor) domain	344 - 409	
OsETR#005	Os07g15540	777	PF01590	GAF domain	184 - 344	Ethylene Receptor
			PF00072	Response regulator receiver domain	646 - 770	
OsMKK#001	Os01g32660	355	PF00069	Protein kinase domain	071 - 330	ACS Type I Activation
OsMKK#002	Os02g54600	369	PF00069	Protein kinase domain	093 - 350	ACS Type I Activation
OsMKK#003	Os03g12390	339	PF00069	Protein kinase domain	049 - 314	EIN3 Activation
OsMKK#004	Os03g50550	345	PF00069	Protein kinase domain	060 - 326	EIN3 Activation
OsMKK#005	Os06g09180	342	PF00069	Protein kinase domain	070 - 327	ACS Type I Activation
OsMPK#001	Os03g17700	369	PF00069	Protein kinase domain	038 - 322	EIN3 Activation
OsMPK#002	Os06g06090	398	PF00069	Protein kinase domain	070 - 352	ACS Type I/EIN3 Activation
OsRAN#001	Os05g49890	221	PF00071	Ras family	015 - 173	Copper Transporter
OsRAN#002	Os06g39875	226	PF00071	Ras family	019 - 177	Copper Transporter
OsRTE#001	Os01g51430	241	PF05608	Protein of unknown function (DUF778)	051 - 186	Positive Regulator of Ethylene Receptor
OsRTE#002	Os03g58520	204	PF05608	Protein of unknown function (DUF778)	027 - 148	Positive Regulator of Ethylene Receptor
OsRTE#003	Os05g46240	233	PF05608	Protein of unknown function (DUF778)	043 - 178	Positive Regulator of Ethylene Receptor

[1] Amino acid residues.
[2] This parameter was determinated by homology with equivalent *Arabidopsis thaliana* amino acid sequence.

Table S5. Soybean Ethylene Biosynthesis Protein Summary

Glycine max Protein			Best Amino Acid Sequence Homology				Soybean Protein Domain			Pathway[2]
			Arabidopsis thaliana		*Oryza sativa*					
Generic Name	Locus ID	Size[1]	Generic Name	Locus ID	Generic Name	Locus ID	PFAM Code	Description	Position	
GmAAT#001	Glyma02g01830	401	AtAAT	At1g77670	OsAAT#002	Os10g34350	PF00155	Aminotransferase class I and II	027 - 388	Yang Cycle
GmAAT#002	Glyma04g43080	450	AtAAT	At1g77670	OsAAT#001	Os09g28050	PF00155	Aminotransferase class I and II	091 - 444	Yang Cycle
GmAAT#003	Glyma06g11630	254	AtAAT	At1g77670	OsAAT#001	Os09g28050	PF00155	Aminotransferase class I and II	001 - 250	Yang Cycle
GmAAT#004	Glyma06g11640	439	AtAAT	At1g77670	OsAAT#001	Os09g28050	PF00155	Aminotransferase class I and II	080 - 433	Yang Cycle
GmACD#001	Glyma05g33540	379	AtACD1	At1g48420	OsACD#001	Os02g53330	PF00291	Pyridoxal-phosphate dependent enzyme	032 - 341	ACC Degradation
GmACD#002	Glyma08g06170	223	AtACD1	At1g48420	OsACD#001	Os02g53330	PF00291	Pyridoxal-phosphate dependent enzyme	013 - 185	ACC Degradation
GmACO#001	Glyma02g43560	315	AtACO4	At1g05010	OsACO#006	Os09g27820	PF03171	2OG-Fe(II) oxygenase superfamily	153 - 253	Ethylene Biosynthesis
GmACO#002	Glyma02g43580	307	AtACO4	At1g05010	OsACO#006	Os09g27820	PF03171	2OG-Fe(II) oxygenase superfamily	150 - 250	Ethylene Biosynthesis
GmACO#003	Glyma02g43600	291	AtACO4	At1g05010	OsACO#006	Os09g27820	PF03171	2OG-Fe(II) oxygenase superfamily	134 - 234	Ethylene Biosynthesis
GmACO#004	Glyma04g42460	308	AtACO#002	At1g77330	OsACO#003	Os05g05680	PF03171	2OG-Fe(II) oxygenase superfamily	154 - 254	Ethylene Biosynthesis
GmACO#005	Glyma05g36310	307	AtACO1	At2g19590	OsACO#004	Os06g37590	PF03171	2OG-Fe(II) oxygenase superfamily	152 - 253	Ethylene Biosynthesis
GmACO#006	Glyma06g12340	307	AtACO#002	At1g77330	OsACO#003	Os05g05680	PF03171	2OG-Fe(II) oxygenase superfamily	153 - 253	Ethylene Biosynthesis
GmACO#007	Glyma07g15480	306	AtACO1	At2g19590	OsACO#004	Os06g37590	PF03171	2OG-Fe(II) oxygenase superfamily	151 - 252	Ethylene Biosynthesis
GmACO#008	Glyma07g39420	318	AtACO4	At1g05010	OsACO#005	Os09g27750	PF03171	2OG-Fe(II) oxygenase superfamily	153 - 253	Ethylene Biosynthesis
GmACO#009	Glyma08g03310	307	AtACO1	At2g19590	OsACO#004	Os06g37590	PF03171	2OG-Fe(II) oxygenase superfamily	152 - 253	Ethylene Biosynthesis
GmACO#010	Glyma08g05500	310	AtACO4	At1g05010	OsACO#006	Os09g27820	PF03171	2OG-Fe(II) oxygenase superfamily	153 - 253	Ethylene Biosynthesis
GmACO#011	Glyma09g01110	318	AtACO4	At1g05010	OsACO#005	Os09g27750	PF03171	2OG-Fe(II) oxygenase superfamily	153 - 253	Ethylene Biosynthesis
GmACO#012	Glyma14g05350	307	AtACO4	At1g05010	OsACO#006	Os09g27820	PF03171	2OG-Fe(II) oxygenase superfamily	150 - 250	Ethylene Biosynthesis
GmACO#013	Glyma14g05360	307	AtACO4	At1g05010	OsACO#006	Os09g27820	PF03171	2OG-Fe(II) oxygenase superfamily	150 - 250	Ethylene Biosynthesis
GmACO#014	Glyma14g05390	315	AtACO4	At1g05010	OsACO#002	Os02g53180	PF03171	2OG-Fe(II) oxygenase superfamily	153 - 253	Ethylene Biosynthesis
GmACO#015	Glyma15g11930	318	AtACO4	At1g05010	OsACO#005	Os09g27750	PF03171	2OG-Fe(II) oxygenase superfamily	153 - 253	Ethylene Biosynthesis
GmACO#016	Glyma17g01330	319	AtACO4	At1g05010	OsACO#005	Os09g27750	PF03171	2OG-Fe(II) oxygenase superfamily	154 - 254	Ethylene Biosynthesis
GmACS#001	Glyma01g00700	442	AtACS7	At4g26200	OsACS#004	Os05g10780	PF00155	Aminotransferase class I and II	044 - 428	Ethylene Biosynthesis
GmACS#002	Glyma01g40400	470	AtACS8	At4g37770	OsACS#002	Os03g51740	PF00155	Aminotransferase class I and II	040 - 423	Ethylene Biosynthesis
GmACS#003	Glyma01g42290	502	AtACS10	At1g62960	OsACS#006	Os06g03990	PF00155	Aminotransferase class I and II	113 - 491	Ethylene Biosynthesis[3]
GmACS#004	Glyma04g05150	437	AtACS8	At4g37770	OsACS#002	Os03g51740	PF00155	Aminotransferase class I and II	040 - 426	Ethylene Biosynthesis
GmACS#005	Glyma05g23020	480	AtACS8	At4g37770	OsACS#002	Os03g51740	PF00155	Aminotransferase class I and II	040 - 427	Ethylene Biosynthesis
GmACS#006	Glyma05g36250	440	AtACS7	At4g26200	OsACS#005	Os05g25490	PF00155	Aminotransferase class I and II	050 - 430	Ethylene Biosynthesis
GmACS#007	Glyma05g37410	434	AtACS1	At3g61510	OsACS#003	Os04g48850	PF00155	Aminotransferase class I and II	001 - 381	Ethylene Biosynthesis
GmACS#008	Glyma06g05240	354	AtACS8	At4g37770	OsACS#002	Os03g51740	PF00155	Aminotransferase class I and II	001 - 341	Ethylene Biosynthesis

Table S5. Soybean Ethylene Biosynthesis Protein Summary (cont.)

Glycine max Protein			Best Amino Acid Sequence Homology				Soybean Protein Domain			Pathway[2]
			Arabidopsis thaliana		*Oryza sativa*					
Generic Name	Locus ID	Size[1]	Generic Name	Locus ID	Generic Name	Locus ID	PFAM Code	Description	Position	
GmACS#009	Glyma07g07160	474	AtACS1	At3g61510	OsACS#003	Os04g48850	PF00155	Aminotransferase class I and II	043 - 424	Ethylene Biosynthesis
GmACS#010	Glyma07g15380	426	AtACS7	At4g26200	OsACS#004	Os05g10780	PF00155	Aminotransferase class I and II	044 - 425	Ethylene Biosynthesis
GmACS#011	Glyma08g02130	484	AtACS6	At4g11280	OsACS#003	Os04g48850	PF00155	Aminotransferase class I and II	048 - 431	Ethylene Biosynthesis
GmACS#012	Glyma08g03400	440	AtACS7	At4g26200	OsACS#004	Os05g10780	PF00155	Aminotransferase class I and II	050 - 430	Ethylene Biosynthesis
GmACS#013	Glyma09g28000	500	AtACS12	At5g51690	OsACS#006	Os06g03990	PF00155	Aminotransferase class I and II	110 - 489	Ethylene Biosynthesis[3]
GmACS#014	Glyma09g39060	485	AtACS1	At3g61510	OsACS#003	Os04g48850	PF00155	Aminotransferase class I and II	047 - 428	Ethylene Biosynthesis
GmACS#015	Glyma11g02390	465	AtACS6	At4g11280	OsACS#003	Os04g48850	PF00155	Aminotransferase class I and II	038 - 421	Ethylene Biosynthesis
GmACS#016	Glyma11g03070	501	AtACS10	At1g62960	OsACS#006	Os06g03990	PF00155	Aminotransferase class I and II	112 - 490	Ethylene Biosynthesis[3]
GmACS#017	Glyma11g04890	471	AtACS8	At4g37770	OsACS#002	Os03g51740	PF00155	Aminotransferase class I and II	040 - 423	Ethylene Biosynthesis
GmACS#018	Glyma16g03600	474	AtACS1	At3g61510	OsACS#003	Os04g48850	PF00155	Aminotransferase class I and II	043 - 424	Ethylene Biosynthesis
GmACS#019	Glyma16g32860	517	AtACS12	At5g51690	OsACS#006	Os06g03990	PF00155	Aminotransferase class I and II	127 - 506	Ethylene Biosynthesis[3]
GmACS#020	Glyma17g16990	475	AtACS8	At4g37770	OsACS#002	Os03g51740	PF00155	Aminotransferase class I and II	040 - 425	Ethylene Biosynthesis
GmACS#021	Glyma18g47280	495	AtACS1	At3g61510	OsACS#003	Os04g48850	PF00155	Aminotransferase class I and II	047 - 428	Ethylene Biosynthesis
GmACT#001	Glyma08g27120	430	AtACT#006	At5g39090	OsACT#001	Os02g57480	PF02458	Transferase family	002 - 429	MACC Synthesis[4]
GmACT#002	Glyma08g27130	447	AtACT#004	At3g29670	OsACT#003	Os07g23150	PF02458	Transferase family	242 - 239	MACC Synthesis[4]
GmACT#003	Glyma08g27500	469	AtACT#001	At1g03940	OsACT#001	Os02g57480	PF02458	Transferase family	005 - 461	MACC Synthesis[4]
GmACT#004	Glyma11g29490	354	AtACT#003	At2g30200	OsACT#002	Os03g18590	PF02458	Transferase family	053 - 353	MACC Synthesis[4]
GmACT#005	Glyma11g29520	230	AtACT#003	At2g30200	OsACT#002	Os03g18590	PF02458	Transferase family	002 - 227	MACC Synthesis[4]
GmACT#006	Glyma12g32630	421	AtACT#002	At1g03495	OsACT#001	Os02g57480	PF02458	Transferase family	002 - 421	MACC Synthesis[4]
GmACT#007	Glyma12g32640	466	AtACT#002	At1g03495	OsACT#003	Os07g23150	PF02458	Transferase family	008 - 453	MACC Synthesis[4]
GmACT#008	Glyma12g32650	443	AtACT#001	At1g03940	OsACT#001	Os02g57480	PF02458	Transferase family	213 - 435	MACC Synthesis[4]
GmACT#009	Glyma12g32660	467	AtACT#001	At1g03940	OsACT#001	Os02g57480	PF02458	Transferase family	008 - 461	MACC Synthesis[4]
GmACT#010	Glyma13g06230	467	AtACT#006	At5g39090	OsACT#001	Os02g57480	PF02458	Transferase family	005 - 462	MACC Synthesis[4]
GmACT#011	Glyma13g06550	449	AtACT#005	At5g39050	OsACT#001	Os02g57480	PF02458	Transferase family	015 - 448	MACC Synthesis[4]
GmACT#012	Glyma13g37810	469	AtACT#001	At1g03940	OsACT#001	Os02g57480	PF02458	Transferase family	002 - 462	MACC Synthesis[4]
GmACT#013	Glyma13g37830	462	AtACT#002	At1g03495	OsACT#001	Os02g57480	PF02458	Transferase family	008 - 450	MACC Synthesis[4]
GmACT#014	Glyma13g37840	405	AtACT#002	At1g03495	OsACT#001	Os02g57480	PF02458	Transferase family	001 - 405	MACC Synthesis[4]
GmACT#015	Glyma13g37850	441	AtACT#002	At1g03495	OsACT#001	Os02g57480	PF02458	Transferase family	010 - 433	MACC Synthesis[4]
GmACT#016	Glyma14g03820	473	AtACT#001	At1g03940	OsACT#003	Os07g23150	PF02458	Transferase family	005 - 464	MACC Synthesis[4]
GmACT#017	Glyma18g06500	358	AtACT#003	At2g30200	OsACT#002	Os03g18590	PF02458	Transferase family	056 - 356	MACC Synthesis[4]

Table S5. Soybean Ethylene Biosynthesis Protein Summary (cont.)

Glycine max Protein			Best Amino Acid Sequence Homology				Soybean Protein Domain			Pathway[2]
			Arabidopsis thaliana		*Oryza sativa*					
Generic Name	Locus ID	Size[1]	Generic Name	Locus ID	Generic Name	Locus ID	PFAM Code	Description	Position	
GmACT#018	Glyma18g49240	511	AtACT#004	At3g29670	OsACT#001	Os02g57480	PF02458	Transferase family	004 - 507	MACC Synthesis[4]
GmACT#019	Glyma18g50310	479	AtACT#005	At5g39050	OsACT#001	Os02g57480	PF02458	Transferase family	006 - 475	MACC Synthesis[4]
GmACT#020	Glyma18g50320	476	AtACT#005	At5g39050	OsACT#001	Os02g57480	PF02458	Transferase family	004 - 473	MACC Synthesis[4]
GmACT#021	Glyma18g50330	452	AtACT#005	At5g39050	OsACT#001	Os02g57480	PF02458	Transferase family	001 - 452	MACC Synthesis[4]
GmACT#022	Glyma18g50340	450	AtACT#006	At5g39090	OsACT#001	Os02g57480	PF02458	Transferase family	001 - 444	MACC Synthesis[4]
GmACT#023	Glyma18g50350	450	AtACT#006	At5g39090	OsACT#001	Os02g57480	PF02458	Transferase family	003 - 444	MACC Synthesis[4]
GmACT#024	Glyma18g50360	389	AtACT#004	At3g29670	OsACT#001	Os02g57480	PF02458	Transferase family	019 - 154	MACC Synthesis[4]
GmACT#025	Glyma19g03730	460	AtACT#006	At5g39090	OsACT#001	Os02g57480	PF02458	Transferase family	004 - 456	MACC Synthesis[4]
GmACT#026	Glyma19g03760	476	AtACT#006	At5g39090	OsACT#001	Os02g57480	PF02458	Transferase family	016 - 472	MACC Synthesis[4]
GmACT#027	Glyma19g03770	464	AtACT#006	At5g39090	OsACT#001	Os02g57480	PF02458	Transferase family	019 - 461	MACC Synthesis[4]
GmACT#028	Glyma19g05220	457	AtACT#002	At1g03495	OsACT#001	Os02g57480	PF02458	Transferase family	012 - 412	MACC Synthesis[4]
GmACT#029	Glyma19g05290	477	AtACT#002	At1g03495	OsACT#001	Os02g57480	PF02458	Transferase family	011 - 471	MACC Synthesis[4]
GmACT#030	Glyma19g11320	451	AtACT#002	At1g03495	OsACT#001	Os02g57480	PF02458	Transferase family	008 - 304	MACC Synthesis[4]
GmARD#001	Glyma10g38940	200	AtARD2	At4g14710	OsARD#001	Os03g06620	PF03079	ARD/ARD' family	014 - 168	Yang Cycle
							PF07883	Cupin domain	088 - 160	
GmARD#002	Glyma10g38960	201	AtARD4	At5g43850	OsARD#004	Os10g28360	PF03079	ARD/ARD' family	018 - 172	Yang Cycle
							PF07883	Cupin domain	092 - 164	
GmARD#003	Glyma20g28850	187	AtARD4	At5g43850	OsARD#001	Os03g06620	PF03079	ARD/ARD' family	004 - 158	Yang Cycle
							PF07883	Cupin domain	078 - 150	
GmARD#004	Glyma20g28860	200	AtARD2	At4g14710	OsARD#001	Os03g06620	PF03079	ARD/ARD' family	014 - 168	Yang Cycle
							PF07883	Cupin domain	088 - 160	
GmASP#001	Glyma04g08560	416	AtASP3	At5g11520	OsASP#001	Os01g55540	PF00155	Aminotransferase class I and II	040 - 408	Yang Cycle
GmASP#002	Glyma06g08670	456	AtASP3	At5g11520	OsASP#001	Os01g55540	PF00155	Aminotransferase class I and II	080 - 448	Yang Cycle
GmASP#003	Glyma14g13480	463	AtASP5	At4g31990	OsASP#002	Os02g55420	PF00155	Aminotransferase class I and II	087 - 455	Yang Cycle
GmASP#004	Glyma17g33050	466	AtASP5	At4g31990	OsASP#002	Os02g55420	PF00155	Aminotransferase class I and II	090 - 458	Yang Cycle
GmDEP#001	Glyma06g36620	518	AtDEP1	At5g53850	OsDEP#001	Os11g29370	PF00596	Class II aldolase and adducin N-terminal domain	027 - 230	Yang Cycle
							PF00702	Haloacid dehalogenase-like hydrolase	276 - 489	
GmDEP#002	Glyma12g35390	522	AtDEP1	At5g53850	OsDEP#001	Os11g29370	PF00596	Class II aldolase and adducin N-terminal domain	030 - 233	Yang Cycle
							PF00702	Haloacid dehalogenase-like hydrolase	280 - 493	

Table S5. Soybean Ethylene Biosynthesis Protein Summary (cont.)

Glycine max Protein			Best Amino Acid Sequence Homology				Soybean Protein Domain			Pathway[2]
			Arabidopsis thaliana		*Oryza sativa*					
Generic Name	Locus ID	Size[1]	Generic Name	Locus ID	Generic Name	Locus ID	PFAM Code	Description	Position	
GmDEP#003	Glyma13g35120	522	AtDEP1	At5g53850	OsDEP#001	Os11g29370	PF00596	Class II aldolase and adducin N-terminal domain	030 - 233	Yang Cycle
							PF00702	Haloacid dehalogenase-like hydrolase	280 - 493	
GmGGT#001	Glyma01g00850	626	AtGGT4	At4g29210	OsGGT#001	Os01g05810	PF01019	Gamma-glutamyltranspeptidase	089 - 621	GACC Synthesis[4]
GmGGT#002	Glyma07g15150	618	AtGGT4	At4g29210	OsGGT#001	Os01g05810	PF01019	Gamma-glutamyltranspeptidase	081 - 613	GACC Synthesis[4]
GmGGT#003	Glyma11g35950	613	AtGGT1	At4g39640	OsGGT#003	Os04g38450	PF01019	Gamma-glutamyltranspeptidase	086 - 608	GACC Synthesis[4]
GmGGT#004	Glyma11g35990	595	AtGGT1	At4g39640	OsGGT#003	Os04g38450	PF01019	Gamma-glutamyltranspeptidase	086 - 590	GACC Synthesis[4]
GmGGT#005	Glyma18g02450	624	AtGGT1	At4g39640	OsGGT#003	Os04g38450	PF01019	Gamma-glutamyltranspeptidase	098 - 619	GACC Synthesis[4]
GmMAT#001	Glyma02g01170	299	AtMAT2	At4g01850	OsMAT#002	Os01g22010	PF00438	S-adenosylmethionine synthetase, N-terminal domain	011 - 084	Ethylene Biosynthesis
							PF02772	S-adenosylmethionine synthetase, central domain	099 - 164	
							PF02773	S-adenosylmethionine synthetase, C-terminal domain	166 - 288	
GmMAT#002	Glyma03g34120	390	AtMAT3	At2g36880	OsMAT#002	Os01g22010	PF00438	S-adenosylmethionine synthetase, N-terminal domain	002 - 101	Ethylene Biosynthesis
							PF02772	S-adenosylmethionine synthetase, central domain	116 - 238	
							PF02773	S-adenosylmethionine synthetase, C-terminal domain	240 - 382	
GmMAT#003	Glyma03g38190	394	AtMAT2	At4g01850	OsMAT#002	Os01g22010	PF00438	S-adenosylmethionine synthetase, N-terminal domain	004 - 103	Ethylene Biosynthesis
							PF02772	S-adenosylmethionine synthetase, central domain	118 - 240	
							PF02773	S-adenosylmethionine synthetase, C-terminal domain	242 - 383	
GmMAT#004	Glyma07g36150	392	AtMAT4	At3g17390	OsMAT#003	Os05g04510	PF00438	S-adenosylmethionine synthetase, N-terminal domain	003 - 102	Ethylene Biosynthesis
							PF02772	S-adenosylmethionine synthetase, central domain	117 - 239	
							PF02773	S-adenosylmethionine synthetase, C-terminal domain	241 - 383	
GmMAT#005	Glyma10g06170	390	AtMAT3	At2g36880	OsMAT#001	Os01g18860	PF00438	S-adenosylmethionine synthetase, N-terminal domain	002 - 101	Ethylene Biosynthesis
							PF02772	S-adenosylmethionine synthetase, central domain	116 - 238	
							PF02773	S-adenosylmethionine synthetase, C-terminal domain	240 - 382	
GmMAT#006	Glyma10g28500	389	AtMAT2	At4g01850	OsMAT#001	Os01g18860	PF00438	S-adenosylmethionine synthetase, N-terminal domain	002 - 101	Ethylene Biosynthesis
							PF02772	S-adenosylmethionine synthetase, central domain	116 - 238	
							PF02773	S-adenosylmethionine synthetase, C-terminal domain	240 - 382	
GmMAT#007	Glyma13g20480	390	AtMAT3	At2g36880	OsMAT#001	Os01g18860	PF00438	S-adenosylmethionine synthetase, N-terminal domain	002 - 101	Ethylene Biosynthesis
							PF02772	S-adenosylmethionine synthetase, central domain	116 - 238	
							PF02773	S-adenosylmethionine synthetase, C-terminal domain	240 - 382	

Table S5. Soybean Ethylene Biosynthesis Protein Summary (cont.)

Glycine max Protein			Best Amino Acid Sequence Homology				Soybean Protein Domain			Pathway[2]
			Arabidopsis thaliana		*Oryza sativa*					
Generic Name	Locus ID	Size[1]	Generic Name	Locus ID	Generic Name	Locus ID	PFAM Code	Description	Position	
GmMAT#008	Glyma15g21890	395	AtMAT4	At3g17390	OsMAT#002	Os01g22010	PF00438	S-adenosylmethionine synthetase, N-terminal domain	004 - 103	Ethylene Biosynthesis
							PF02772	S-adenosylmethionine synthetase, central domain	118 - 240	
							PF02773	S-adenosylmethionine synthetase, C-terminal domain	242 - 384	
GmMAT#009	Glyma17g04330	392	AtMAT4	At3g17390	OsMAT#003	Os05g04510	PF00438	S-adenosylmethionine synthetase, N-terminal domain	003 - 102	Ethylene Biosynthesis
							PF02772	S-adenosylmethionine synthetase, central domain	117 - 239	
							PF02773	S-adenosylmethionine synthetase, C-terminal domain	241 - 383	
GmMAT#010	Glyma17g04340	392	AtMAT4	At3g17390	OsMAT#003	Os05g04510	PF00438	S-adenosylmethionine synthetase, N-terminal domain	003 - 102	Ethylene Biosynthesis
							PF02772	S-adenosylmethionine synthetase, central domain	117 - 239	
							PF02773	S-adenosylmethionine synthetase, C-terminal domain	241 - 383	
GmMAT#011	Glyma19g40810	394	AtMAT2	At4g01850	OsMAT#002	Os01g22010	PF00438	S-adenosylmethionine synthetase, N-terminal domain	004 - 103	Ethylene Biosynthesis
							PF02772	S-adenosylmethionine synthetase, central domain	118 - 240	
							PF02773	S-adenosylmethionine synthetase, C-terminal domain	242 - 383	
GmMTI#001	Glyma09g08190	286	AtMTI1	At2g05830	OsMTI#001	Os11g11050	PF01008	Initiation factor 2 subunit family	054 - 272	Yang Cycle
GmMTI#002	Glyma15g19690	200	AtMTI1	At2g05830	OsMTI#001	Os11g11050	PF01008	Initiation factor 2 subunit family	001 - 189	Yang Cycle
GmMTK#001	Glyma01g45520	425	AtMTK1	At1g49820	OsMTK#001	Os04g57400	PF01636	Phosphotransferase enzyme family	037 - 251	Yang Cycle
GmMTK#002	Glyma10g40730	417	AtMTK1	At1g49820	OsMTK#001	Os04g57400	PF01636	Phosphotransferase enzyme family	036 - 296	Yang Cycle
GmMTK#003	Glyma20g26580	417	AtMTK1	At1g49820	OsMTK#001	Os04g57400	PF01636	Phosphotransferase enzyme family	036 - 296	Yang Cycle
GmMTN#001	Glyma04g00770	265	AtMTN1	At4g38800	OsMTN#001	Os06g02220	PF01048	Phosphorylase superfamily	026 - 255	Yang Cycle
GmMTN#002	Glyma06g00790	266	AtMTN1	At4g38800	OsMTN#001	Os06g02220	PF01048	Phosphorylase superfamily	027 - 256	Yang Cycle
GmMTN#003	Glyma06g00800	253	AtMTN1	At4g38800	OsMTN#001	Os06g02220	PF01048	Phosphorylase superfamily	014 - 243	Yang Cycle

[1] Amino acid residues.
[2] This parameter was determinated by homology with equivalent *Arabidopsis thaliana* amino acid sequence.
[3] Putative amino acid transferase (AAT). No ACC synthase activity demonstrated.
[4] The primary function of this protein is not initially associated with this metabolic pathway.

Table S6. Soybean Ethylene Signal Transduction Protein Summary

Glycine max Protein			Best Amino Acid Sequence Homology				Soybean Protein Domain			Putative Function[2]
			Arabidopsis thaliana		*Oryza sativa*					
Generic Name	Locus ID	Size[1]	Generic Name	Locus ID	Generic Name	Locus ID	PFAM Code	Description	Position	
GmCPK#001	Glyma04g38150	496	AtCDPK2	At1g35670	OsCPK#001	Os11g07040	PF00069	Protein kinase domain	030 - 288	ACS Type II Activation
							PF00036	EF hand	335 - 396	
									401 - 466	
GmCPK#002	Glyma05g33240	507	AtCDPK2	At1g35670	OsCPK#001	Os11g07040	PF00069	Protein kinase domain	033 - 291	ACS Type II Activation
							PF00036	EF hand	338 - 399	
									404 - 469	
GmCPK#003	Glyma06g16920	497	AtCDPK2	At1g35670	OsCPK#001	Os11g07040	PF00069	Protein kinase domain	031 - 289	ACS Type II Activation
							PF00036	EF hand	336 - 397	
									402 - 467	
GmCPK#004	Glyma08g00840	508	AtCDPK2	At1g35670	OsCPK#001	Os11g07040	PF00069	Protein kinase domain	034 - 292	ACS Type II Activation
							PF00036	EF hand	339 - 400	
									405 - 470	
GmCPK#005	Glyma10g36090	482	AtCDPK2	At1g35670	OsCPK#002	Os12g07230	PF00069	Protein kinase domain	021 - 279	ACS Type II Activation
							PF00036	EF hand	326 - 387	
									392 - 457	
GmCTR#001	Glyma02g27680	660	AtCTR1	At5g03730	OsCTR#001	Os02g32610	PF14381	Ethylene responsive protein kinase Le-CTR1	064 - 273	Negative Regulator of Ethylene Response
							PF07714	Protein tyrosine kinase	397 - 651	
GmCTR#002	Glyma03g34890	803	AtCTR1	At5g03730	OsCTR#001	Os02g32610	PF14381	Ethylene responsive protein kinase Le-CTR1	172 - 371	Negative Regulator of Ethylene Response
							PF07714	Protein tyrosine kinase	529 - 783	
GmCTR#003	Glyma10g07610	793	AtCTR1	At5g03730	OsCTR#001	Os02g32610	PF14381	Ethylene responsive protein kinase Le-CTR1	197 - 396	Negative Regulator of Ethylene Response
							PF07714	Protein tyrosine kinase	505 - 760	
GmCTR#004	Glyma13g21480	836	AtCTR1	At5g03730	OsCTR#001	Os02g32610	PF14381	Ethylene responsive protein kinase Le-CTR1	176 - 386	Negative Regulator of Ethylene Response
							PF07714	Protein tyrosine kinase	562 - 816	
GmCTR#005	Glyma19g37570	803	AtCTR1	At5g03730	OsCTR#001	Os02g32610	PF14381	Ethylene responsive protein kinase Le-CTR1	167 - 376	Negative Regulator of Ethylene Response
							PF07714	Protein tyrosine kinase	529 - 783	
GmEBF#001	Glyma04g07110	636	AtEBF1	At2g25490	OsEBF#001	Os02g10700	PF00646	F-box domain	065 - 113	Directs EIN3 to Proteolysis
GmEBF#002	Glyma04g20330	650	AtEBF1	At2g25490	OsEBF#001	Os02g10700	PF00646	F-box domain	068 - 106	Directs EIN3 to Proteolysis
GmEBF#003	Glyma06g07200	638	AtEBF1	At2g25490	OsEBF#001	Os02g10700	PF00646	F-box domain	067 - 114	Directs EIN3 to Proteolysis
GmEBF#004	Glyma13g23510	639	AtEBF1	At2g25490	OsEBF#001	Os02g10700	PF00646	F-box domain	065 - 103	Directs EIN3 to Proteolysis
GmEBF#005	Glyma14g14410	644	AtEBF1	At2g25490	OsEBF#001	Os02g10700	PF00646	F-box domain	065 - 111	Directs EIN3 to Proteolysis

Table S6. Soybean Ethylene Signal Transduction Protein Summary (cont.)

Glycine max Protein			Best Amino Acid Sequence Homology				Soybean Protein Domain			Putative Function[2]
			Arabidopsis thaliana		*Oryza sativa*					
Generic Name	Locus ID	Size[1]	Generic Name	Locus ID	Generic Name	Locus ID	PFAM Code	Description	Position	
GmEBF#006	Glyma17g12270	639	AtEBF1	At2g25490	OsEBF#001	Os02g10700	PF00646	F-box domain	065 - 103	Directs EIN3 to Proteolysis
GmEBF#007	Glyma17g31940	610	AtEBF1	At2g25490	OsEBF#001	Os02g10700	PF00646	F-box domain	067 - 106	Directs EIN3 to Proteolysis
GmEIN#001	Glyma02g44220	614	AtEIN3	At3g20770	OsEIN#002	Os03g20780	PF04873	Ethylene insensitive 3	048 - 426	Nuclear Transcription Factor
GmEIN#002	Glyma03g33850	1281	AtEIN2	At5g03280	OsEIN#006	Os07g06130	PF01566	Natural resistance-associated macrophage protein	038 - 392	Acts Downstream of CTR1
GmEIN#003	Glyma03g40500	966	AtEIN5	At1g54490	OsEIN#005	Os03g58060	PF03159	XRN 5'-3' exonuclease N-terminus	001 - 257	EBF RNA Silencing
							PF00098	Zinc knuckle	264 - 281	
GmEIN#004	Glyma10g06610	1298	AtEIN2	At5g03280	OsEIN#006	Os07g06130	PF01566	Natural resistance-associated macrophage protein	038 - 392	Acts Downstream of CTR1
GmEIN#005	Glyma13g03660	618	AtEIN3	At3g20770	OsEIN#003	Os03g20790	PF04873	Ethylene insensitive 3	049 - 426	Nuclear Transcription Factor
GmEIN#006	Glyma13g03700	621	AtEIN3	At3g20770	OsEIN#002	Os03g20780	PF04873	Ethylene insensitive 3	049 - 427	Nuclear Transcription Factor
GmEIN#007	Glyma13g20810	1334	AtEIN2	At5g03280	OsEIN#006	Os07g06130	PF01566	Natural resistance-associated macrophage protein	038 - 392	Acts Downstream of CTR1
GmEIN#008	Glyma14g04550	610	AtEIN3	At3g20770	OsEIN#003	Os03g20790	PF04873	Ethylene insensitive 3	048 - 426	Nuclear Transcription Factor
GmEIN#009	Glyma19g43160	957	AtEIN5	At1g54490	OsEIN#005	Os03g58060	PF03159	XRN 5'-3' exonuclease N-terminus	001 - 255	EBF RNA Silencing
							PF00098	Zinc knuckle	262 - 279	
GmEIN#010	Glyma20g12250	624	AtEIN3	At3g20770	OsEIN#002	Os03g20780	PF04873	Ethylene insensitive 3	048 - 423	Nuclear Transcription Factor
GmEIN#011	Glyma20g37260	932	AtEIN5	At1g54490	OsEIN#005	Os03g58060	PF03159	XRN 5'-3' exonuclease N-terminus	001 - 256	EBF RNA Silencing
							PF00098	Zinc knuckle	263 - 280	
GmETO#001	Glyma01g31170	954	AtETO1	At3g51770	OsETO#001	Os03g18360	PF13414	TPR repeat	871 - 934	ACS Type II Negative Regulator
GmETO#002	Glyma02g16310	886	AtEOL1	At4g02680	OsETO#003	Os11g37520	PF00515	Tetratricopeptide repeat	709 - 742	ACS Type II Negative Regulator
GmETO#003	Glyma02g39460	937	AtETO1	At3g51770	OsETO#001	Os03g18360	PF07719	Tetratricopeptide repeat	560 - 593	ACS Type II Negative Regulator
GmETO#004	Glyma03g06600	960	AtETO1	At3g51770	OsETO#001	Os03g18360	PF13414	TPR repeat	875 - 940	ACS Type II Negative Regulator
GmETO#005	Glyma03g31370	888	AtEOL1	At4g02680	OsETO#002	Os07g08120	PF13414	TPR repeat	709 - 777	ACS Type II Negative Regulator
GmETO#006	Glyma10g03520	887	AtEOL1	At4g02680	OsETO#003	Os11g37520	PF00515	Tetratricopeptide repeat	710 - 743	ACS Type II Negative Regulator
GmETO#007	Glyma11g29000	935	AtETO1	At3g51770	OsETO#001	Os03g18360	PF13414	TPR repeat	853 - 918	ACS Type II Negative Regulator
GmETO#008	Glyma14g37640	924	AtETO1	At3g51770	OsETO#001	Os03g18360	PF13414	TPR repeat	745 - 810	ACS Type II Negative Regulator
GmETO#009	Glyma18g06680	873	AtETO1	At3g51770	OsETO#001	Os03g18360	PF13414	TPR repeat	742 - 807	ACS Type II Negative Regulator
GmETO#010	Glyma19g34200	886	AtEOL1	At4g02680	OsETO#003	Os11g37520	PF13414	TPR repeat	707 - 772	ACS Type II Negative Regulator
GmETR#001	Glyma03g37470	636	AtERS1	At2g40940	OsETR#002	Os03g49500	PF01590	GAF domain	159 - 308	Ethylene Receptor
							PF00512	Histidine kinase A (phosphoacceptor) domain	344 - 409	
							PF02518	Histidine kinase$^-$, DNA gyrase B$^-$ and HSP90-like ATPase	456 - 587	

Table S6. Soybean Ethylene Signal Transduction Protein Summary (cont.)

Glycine max Protein			Best Amino Acid Sequence Homology				Soybean Protein Domain			Putative Function[2]
			Arabidopsis thaliana		*Oryza sativa*					
Generic Name	Locus ID	Size[1]	Generic Name	Locus ID	Generic Name	Locus ID	PFAM Code	Description	Position	
GmETR#002	Glyma03g41220	760	AtEIN4	At3g04580	OsETR#003	Os04g08740	PF01590	GAF domain	183 - 334	Ethylene Receptor
							PF00512	Histidine kinase A (phosphoacceptor) domain	370 - 435	
							PF00072	Response regulator receiver domain	636 - 750	
GmETR#003	Glyma09g00490	740	AtETR1	At1g66340	OsETR#002	Os03g49500	PF01590	GAF domain	158 - 307	Ethylene Receptor
							PF00512	Histidine kinase A (phosphoacceptor) domain	343 - 408	
							PF02518	Histidine kinase-, DNA gyrase B- and HSP90-like ATPase	455 - 586	
							PF00072	Response regulator receiver domain	616 - 728	
GmETR#004	Glyma10g31040	767	AtEIN4	At3g04580	OsETR#003	Os04g08740	PF01590	GAF domain	192 - 341	Ethylene Receptor
							PF00512	Histidine kinase A (phosphoacceptor) domain	377 - 442	
							PF00072	Response regulator receiver domain	643 - 757	
GmETR#005	Glyma10g33240	751	AtETR2	At3g23150	OsETR#003	Os04g08740	PF01590	GAF domain	182 - 331	Ethylene Receptor
							PF00512	Histidine kinase A (phosphoacceptor) domain	367 - 432	
							PF00072	Response regulator receiver domain	627 - 741	
GmETR#006	Glyma12g37050	739	AtETR1	At1g66340	OsETR#002	Os03g49500	PF01590	GAF domain	158 - 307	Ethylene Receptor
							PF00512	Histidine kinase A (phosphoacceptor) domain	343 - 408	
							PF02518	Histidine kinase-, DNA gyrase B- and HSP90-like ATPase	455 - 585	
							PF00072	Response regulator receiver domain	615 - 727	
GmETR#007	Glyma19g40090	636	AtERS1	At2g40940	OsETR#002	Os03g49500	PF01590	GAF domain	159 - 308	Ethylene Receptor
							PF00512	Histidine kinase A (phosphoacceptor) domain	344 - 409	
							PF02518	Histidine kinase-, DNA gyrase B- and HSP90-like ATPase	456 - 587	
GmETR#008	Glyma19g43840	731	AtEIN4	At3g04580	OsETR#003	Os04g08740	PF01590	GAF domain	183 - 333	Ethylene Receptor
							PF00512	Histidine kinase A (phosphoacceptor) domain	369 - 434	
							PF00072	Response regulator receiver domain	635 - 713	
GmETR#009	Glyma20g21780	682	AtETR2	At3g23150	OsETR#003	Os04g08740	PF01590	GAF domain	186 - 332	Ethylene Receptor
							PF00512	Histidine kinase A (phosphoacceptor) domain	368 - 433	
							PF00072	Response regulator receiver domain	559 - 672	
GmETR#010	Glyma20g34420	798	AtETR2	At3g23150	OsETR#003	Os04g08740	PF01590	GAF domain	182 - 331	Ethylene Receptor
							PF00512	Histidine kinase A (phosphoacceptor) domain	367 - 432	
							PF00072	Response regulator receiver domain	674 - 788	

Table S6. Soybean Ethylene Signal Transduction Protein Summary (cont.)

Glycine max Protein			Best Amino Acid Sequence Homology				Soybean Protein Domain			Putative Function[2]
			Arabidopsis thaliana		*Oryza sativa*					
Generic Name	Locus ID	Size[1]	Generic Name	Locus ID	Generic Name	Locus ID	PFAM Code	Description	Position	
GmETR#011	Glyma20g36440	734	AtEIN4	At3g04580	OsETR#003	Os04g08740	PF01590	GAF domain	159 - 308	Ethylene Receptor
							PF00512	Histidine kinase A (phosphoacceptor) domain	344 - 409	
							PF00072	Response regulator receiver domain	610 - 724	
GmMKK#001	Glyma07g00520	351	AtMKK4	At1g51660	OsMKK#005	Os06g09180	PF00069	Protein kinase domain	069 - 324	ACS Type I Activation
GmMKK#002	Glyma07g11910	318	AtMKK9	At1g73500	OsMKK#003	Os03g12390	PF00069	Protein kinase domain	049 - 311	EIN3 Activation
GmMKK#003	Glyma08g23900	364	AtMKK4	At1g51660	OsMKK#005	Os06g09180	PF00069	Protein kinase domain	082 - 337	ACS Type I Activation
GmMKK#004	Glyma09g30300	319	AtMKK9	At1g73500	OsMKK#003	Os03g12390	PF00069	Protein kinase domain	050 - 312	EIN3 Activation
GmMPK#001	Glyma02g15690	391	AtMPK6	At2g43790	OsMPK#002	Os06g06090	PF00069	Protein kinase domain	061 - 344	ACS Type I/EIN3 Activation
GmMPK#002	Glyma07g32750	433	AtMPK6	At2g43790	OsMPK#002	Os06g06090	PF00069	Protein kinase domain	103 - 386	ACS Type I/EIN3 Activation
GmMPK#003	Glyma11g15700	371	AtMPK3	At3g45640	OsMPK#001	Os03g17700	PF00069	Protein kinase domain	041 - 325	EIN3 Activation
GmMPK#004	Glyma12g07770	371	AtMPK3	At3g45640	OsMPK#001	Os03g17700	PF00069	Protein kinase domain	041 - 325	EIN3 Activation
GmRAN#001	Glyma04g07350	221	AtRAN3	At5g55190	OsRAN#001	Os05g49890	PF00071	Ras family	015 - 173	Copper Transporter
GmRAN#002	Glyma04g07360	221	AtRAN3	At5g55190	OsRAN#001	Os05g49890	PF00071	Ras family	015 - 173	Copper Transporter
GmRAN#003	Glyma04g07370	221	AtRAN3	At5g55190	OsRAN#001	Os05g49890	PF00071	Ras family	015 - 173	Copper Transporter
GmRAN#004	Glyma05g08260	221	AtRAN3	At5g55190	OsRAN#001	Os05g49890	PF00071	Ras family	015 - 173	Copper Transporter
GmRAN#005	Glyma06g07400	221	AtRAN3	At5g55190	OsRAN#001	Os05g49890	PF00071	Ras family	015 - 173	Copper Transporter
GmRAN#006	Glyma06g07410	221	AtRAN3	At5g55190	OsRAN#001	Os05g49890	PF00071	Ras family	015 - 173	Copper Transporter
GmRAN#007	Glyma06g07420	221	AtRAN3	At5g55190	OsRAN#001	Os05g49890	PF00071	Ras family	015 - 173	Copper Transporter
GmRTE#001	Glyma01g37560	231	AtRTH1	At3g51040	OsRTE#002	Os03g58520	PF05608	Protein of unknown function (DUF778)	040 - 175	Positive Regulator of Ethylene Receptor
GmRTE#002	Glyma11g07740	230	AtRTH1	At3g51040	OsRTE#002	Os03g58520	PF05608	Protein of unknown function (DUF778)	039 - 174	Positive Regulator of Ethylene Receptor
GmRTE#003	Glyma14g10770	235	AtRTE1	At2g26070	OsRTE#001	Os01g51430	PF05608	Protein of unknown function (DUF778)	044 - 179	Positive Regulator of Ethylene Receptor
GmRTE#004	Glyma17g34740	235	AtRTE1	At2g26070	OsRTE#001	Os01g51430	PF05608	Protein of unknown function (DUF778)	044 - 179	Positive Regulator of Ethylene Receptor

[1] Amino acid residues.
[2] This parameter was determinated by homology with equivalent *Arabidopsis thaliana* amino acid sequence.

Table S7. BBH Experiment - Soybean Ethylene Biosynthesis Proteins[1,2]

Glycine max Protein		*Arabidopsis thaliana* Protein		*Oryza sativa* Protein	
Generic Name	Locus ID	Generic Name	Locus ID	Generic Name	Locus ID
GmAAT#001	Glyma02g01830	–	–	OsAAT#002	Os10g34350
GmAAT#004	Glyma06g11640	AtAAT	At1g77670	OsAAT#001	Os09g28050
GmACD#001	Glyma05g33540	AtACD1	At1g48420	OsACD#001	Os02g53330
GmACO#004	Glyma04g42460	AtACO#002	At1g77330	OsACO#003	Os05g05680
GmACO#006	Glyma06g12340	AtACO#002	At1g77330	OsACO#003	Os05g05680
GmACO#007	Glyma07g15480	AtACO1	At2g19590	–	–
GmACO#008	Glyma07g39420	–	–	OsACO#005	Os09g27750
GmACO#009	Glyma08g03310	–	–	OsACO#004	Os06g37590
GmACO#014	Glyma14g05390	AtACO4	At1g05010	–	–
GmACS#006	Glyma05g36250	AtACS7	At4g26200	–	–
GmACS#011	Glyma08g02130	AtACS6	At4g11280	OsACS#003	Os04g48850
GmACS#012	Glyma08g03400	–	–	OsACS#004	Os05g10780
GmACS#013	Glyma09g28000	AtACS12	At5g51690	OsACS#006	Os06g03990
GmACS#014	Glyma09g39060	AtACS1	At3g61510	–	–
GmACS#016	Glyma11g03070	AtACS10	At1g62960	–	–
GmACS#017	Glyma11g04890	AtACS8	At4g37770	–	–
GmACS#020	Glyma17g16990	–	–	OsACS#002	Os03g51740
GmACT#003	Glyma08g27500	AtACT#001	At1g03940	–	–
GmACT#006	Glyma12g32630	AtACT#002	At1g03495	–	–
GmACT#017	Glyma18g06500	AtACT#003	At2g30200	OsACT#002	Os03g18590
GmACT#020	Glyma18g50320	AtACT#005	At5g39050	–	–
GmACT#023	Glyma18g50350	–	–	OsACT#001	Os02g57480
GmARD#001	Glyma10g38940	–	–	OsARD#001	Os03g06620
GmARD#002	Glyma10g38960	AtARD4	At5g43850	OsARD#004	Os10g28360
GmARD#004	Glyma20g28860	AtARD2	At4g14710	–	–
GmASP#002	Glyma06g08670	AtASP3	At5g11520	OsASP#001	Os01g55540
GmASP#004	Glyma17g33050	AtASP5	At4g31990	OsASP#003	Os02g55420
GmDEP#001	Glyma06g36620	AtDEP1	At5g53850	–	–
GmDEP#002	Glyma12g35390	–	–	OsDEP#001	Os11g29370
GmGGT#001	Glyma01g00850	AtGGT4	At4g29210	OsGGT#001	Os01g05810
GmGGT#003	Glyma11g35950	AtGGT1	At4g39640	OsGGT#003	Os04g38450
GmMAT#007	Glyma13g20480	AtMAT3	At2g36880	–	–
GmMAT#008	Glyma15g21890	AtMAT4	At3g17390	–	–
GmMAT#009	Glyma17g04330	–	–	OsMAT#003	Os05g04510
GmMAT#011	Glyma19g40810	AtMAT2	At4g01850	OsMAT#002	Os01g22010
GmMTI#001	Glyma09g08190	AtMTI1	At2g05830	OsMTI#001	Os11g11050
GmMTI#002	Glyma15g19690	AtMTI1	At2g05830	–	–
GmMTK#003	Glyma20g26580	AtMTK1	At1g49820	OsMTK#001	Os04g57400
GmMTN#001	Glyma04g00770	AtMTN1	At4g38800	–	–
GmMTN#002	Glyma06g00790	–	–	OsMTN#001	Os06g02220

[1] Soybean ethylene biosynthesis proteins that have a homologous BBH positive with *Arabidopsis thaliana* and/or *Oryza sativa*.
[2] BBH: Best Bidirectional Hit.

Table S8. BBH Experiment - Soybean Proteins Related with Ethylene Signal Transduction[1,2]

Glycine max Protein		*Arabidopsis thaliana* Protein		*Oryza sativa* Protein	
Generic Name	Locus ID	Generic Name	Locus ID	Generic Name	Locus ID
GmCPK#004	Glyma08g00840	–	–	OsCPK#001	Os11g07040
GmCTR#004	Glyma13g21480	AtCTR1	At5g03730	OsCTR#001	Os02g32610
GmEBF#005	Glyma14g14410	AtEBF1	At2g25490	OsEBF#001	Os02g10700
GmEIN#007	Glyma13g20810	AtEIN2	At5g03280	OsEIN#006	Os07g06130
GmEIN#008	Glyma14g04550	–	–	OsEIN#003	Os03g20790
GmEIN#009	Glyma19g43160	AtEIN5	At1g54490	–	–
GmEIN#010	Glyma20g12250	AtEIN3	At3g20770	OsEIN#002	Os03g20780
GmEIN#011	Glyma20g37260	–	–	OsEIN#005	Os03g58060
GmETO#001	Glyma01g31170	AtETO1	At3g51770	OsETO#001	Os03g18360
GmETO#002	Glyma02g16310	–	–	OsETO#003	Os11g37520
GmETO#010	Glyma19g34200	AtEOL1	At4g02680	–	–
GmETR#002	Glyma03g41220	AtEIN4	At3g04580	OsETR#003	Os04g08740
GmETR#003	Glyma09g00490	–	–	OsETR#002	Os03g49500
GmETR#006	Glyma12g37050	AtETR1	At1g66340	–	–
GmETR#007	Glyma19g40090	AtERS1	At2g40940	–	–
GmETR#010	Glyma20g34420	AtETR2	At3g23150	–	–
GmMKK#001	Glyma07g00520	AtMKK4	At1g51660	OsMKK#005	Os06g09180
GmMKK#002	Glyma07g11910	AtMKK9	At1g73500	OsMKK#001	Os03g12390
GmMPK#001	Glyma02g15690	AtMPK6	At2g43790	OsMPK#002	Os06g06090
GmMPK#004	Glyma12g07770	AtMPK3	At3g45640	OsMPK#001	Os03g17700
GmRAN#002	Glyma04g07360	–	–	OsRAN#001	Os05g49890
GmRAN#003	Glyma04g07370	–	–	OsRAN#001	Os05g49890
GmRAN#005	Glyma06g07400	AtRAN3	At5g55190	–	–
GmRAN#006	Glyma06g07410	–	–	OsRAN#001	Os05g49890
GmRTE#002	Glyma11g07740	AtRTH1	At3g51040	OsRTE#002	Os03g58520
GmRTE#003	Glyma14g10770	AtRTE1	At2g26070	OsRTE#001	Os01g51430

[1] Soybean proteins related with ethylene signal transduction that have a homologous BBH positive with *Arabidopsis thaliana* and/or *Oryza sativa*.
[2] BBH: Best Bidirectional Hit.
