# Supplementary material for: Implications of ethylene biosynthesis and signaling in soybean drought stress tolerance
Source: BMC Plant Biol. 2015 Sep 3;15:213. doi: 10.1186/s12870-015-0597-z (PMC4557918; doi:10.1186/s12870-015-0597-z)
Supplement: Additional file 3: — In silico Characterization of Ethylene Soybean Genes. Detailed description of characterization, gene localization and gene onthology (GO) of ethylene soybean genes. (PDF 12 kb) [file 12870_2015_597_MOESM3_ESM.pdf]

In silico Characterization of Ethylene Soybean Genes

Characterization of Ethylene Soybean Genes

The genes were divided into 2 principal groups: ethylene biosynthesis and ethylene signal transduction. The ethylene biosynthesis genes in *A. thaliana*, *O. sativa* and *Glycine max* corresponded to 59.5%, 52.8% and 61.4% of the total of identified genes in each species, respectively. Regarding ethylene signal transduction, they corresponded to 40.5%, 47.2% and 38.6%, respectively. As can be observed, the relative quantity of genes in each group is similar between the three species. In addition, the *cis*-acting elements present in putative soybean promoters were analyzed, in order to further refine the models obtained.

Gene Localization

The 176 soybean genes are shown as they are situated among the 20 *G. max* chromosomes (Fig. S1), revealing that the majority are located in the extremities. Among them, 48.9% are in the sense strand and 51.1% in the anti-sense strand of DNA. Chromosome 13 has the largest number of identified genes (13 genes). Chromosomes 18 and 3 have the highest number of genes related to ethylene biosynthesis (10 genes) and signal transduction (7 genes). Chromosomes 15 and 16 do not have any member of the signal transduction group. Normalization (number of genes per megabase in each chromosome) revealed that the highest density of genes associated with ethylene biosynthesis is within chromosomes 13 and 18, as chromosome 3 presented the highest density of genes associated with ethylene signal transduction.

Gene Ontology (GO)

The functional annotation of the ontology of the 176 soybean genes resulted in a distribution into three categories: cellular components, molecular function and biological processes (Fig. S2). With respect to the cellular components found for level 2 (Fig. S2A), 27.6% were in the cell, 8.6% in the cell junction, 12.1% in the extracellular region, 1.6% in the macromolecular complexes, 4.5% in the extracellular regions, 14.1% in the membrane, 4.0% in the membrane-enclosed-lumen, 23.4% in organelles and 8.6% in the symplast. With regard to the detected molecular function [also in level 2 (Fig. S2B)], 1.0% are related to transport, 48.1% to catalytic activity, 1.6% to nucleic acid binding, 7.6% to molecular transducer activity, 1.3% to molecular function regulation and 40.4% related to general binding. Lastly, genes for biological processes (Fig. S2C) were found to be related to biological adhesion and regulation (0.2% and 7.6%, respectively); cellular component organization or biogenesis (6.4%); cellular and developmental processes (10.1% and 7.3%,

respectively); growth (5.4%); immune system processes (3.6%); localization (6.9%); locomotion (0.2%); metabolic, multicellular organismal and multi-organism processes (10.4%, 7.2% and 5.7%, respectively); reproduction and reproductive processes (2.2% and 3.3%, respectively); response to stimulus (9.6%); rhythmic processes (0.9%); signaling (5.4%); and single organism processes (10.7%). Some of the sequences were assigned to more than one category, resulting in a total number greater than 176. Additionally, sometimes, many functions were assigned to the same sequence such that the number of total functions per category exceeded 176.

Protein Characterization and Orthology Analysis

We analyzed the proteins codified by the genes identified in *A. thaliana* (Tables S1 and S2), *O. sativa* (Tables S3 and S4) and *Glycine max* (Tables S5 and S6), in which the principal characteristics of the amino acid residues of each sequence were determined: the length and presence/localization of conserved domains. We also distinguished the homologous sequences between soybean and *A. thaliana* and *O. sativa*, which facilitated the detection of these protein functions. According to the *PFAM* database, in the three species, a total of 33 conserved protein domains were observed, with 15 of them belonging the ethylene biosynthesis and 19 to the signal transduction group.

To identify possible soybean orthologous proteins in *A. thaliana* and *O. sativa,* we performed a BBH (best bidirectional hit) analysis comparing the three species databases. Within the 176 proteins codified by genes, 66 positive BHH events were identified with *A. thaliana* and/or *O. sativa,* with 60.6% related to ethylene biosynthesis and 39.4% related to ethylene signal transduction (Tables S7 and S8). Concerning the total number of soybean proteins identified in each group, 13.9% of those belonging to ethylene biosynthesis were BHH positive with *A. thaliana* proteins, 9.3% with *O. sativa* and 13.9% with both species. Among the proteins involved in ethylene signal transduction, 8.8% BHH were positive with *A. thaliana*, 11.8% with *O. sativa* and 17.6% with both species (Fig. S3). Hence, with the exception of signal transduction proteins interacting with ethylene, the other protein group studied (ethylene biosynthesis) presented more orthologs with *A. thaliana* than with *O. sativa*, indicating a closer phylogenetic relationship between soybean and *A. thaliana* and suggesting high similarity between the metabolic pathways of both plants.
