# Supplementary material for: Implications of ethylene biosynthesis and signaling in soybean drought stress tolerance
Source: BMC Plant Biol. 2015 Sep 3;15:213. doi: 10.1186/s12870-015-0597-z (PMC4557918; doi:10.1186/s12870-015-0597-z)
Supplement: Additional file 5: — Real Time PCR (RT-qPCR) Cycle Threshold (C t ) (Tables S10-S11). Table S10. Target and endogenous gene cycle threshold in soybean leaf under drought stress – RT-qPCR; Table S11. Target and endogenous gene cycle threshold in soybean root under drought stress – RT-qPCR. (PDF 326 kb) [file 12870_2015_597_MOESM5_ESM.pdf]

Table S10. Target and Endogenous Gene Cycle Threshold in Soybean Leaf Under Drought Stress – RT-qPCR[1]

Symbol	Generic Name	c.D.[2]	Time of Drought Stress (minute)													
			0		25		50		75		100		125		150	
			BR16[3]	E48[4]	BR16[3]	E48[4]	BR16[3]	E48[4]	BR16[3]	E48[4]	BR16[3]	E48[4]	BR16[3]	E48[4]	BR16[3]	E48[4]
ACO	GmACO#012	1:80	22.1±0.2	23.4±1.1	20.6±0.4	22.4±0.4	21.6±0.4	22.5±0.5	22.3±0.4	22.5±0.2	22.1±0.1	20.7±0.1	20.6±0.4	19.9±0.1	18.9±0.1	20.6±0.1
ACS	GmACS#006	1:40	30.9±0.2	35.0±0.8	29.8±0.2	30.2±0.2	29.5±0.2	31.4±0.1	31.1±0.1	31.3±0.2	30.4±0.2	30.7±0.6	31.4±0.3	32.9±1.2	32.4±0.4	33.2±0.4
CTR	GmCTR#003	1:40	30.2±0.1	30.6±0.1	29.9±0.1	30.7±0.1	30.9±0.2	30.5±0.1	31.1±0.1	30.4±0.1	31.2±0.2	29.8±0.2	30.0±0.2	31.9±0.1	31.1±0.1	31.8±0.1
ETR	GmETR#007	1:40	21.9±0.3	22.9±0.2	22.0±0.1	22.3±0.1	22.7±0.4	22.5±0.1	22.7±0.3	22.1±0.3	23.3±0.1	22.2±0.2	22.3±0.2	22.3±0.1	20.6±0.1	22.6±0.7
MAT	GmMAT#008	1:80	19.2±0.2	19.3±0.4	17.9±0.3	19.0±0.2	18.7±0.1	18.8±0.1	18.1±0.3	18.5±0.2	19.6±0.1	17.2±0.3	17.7±0.3	19.4±0.5	17.8±0.4	18.0±0.2
–	ACT11	1:40	18.4±0.5	18.9±0.8	17.7±0.6	18.3±0.1	18.5±0.4	18.5±0.1	19.1±0.5	18.4±0.2	18.7±1.2	18.1±0.6	18.8±0.3	19.7±0.5	19.6±0.1	20.3±0.4
–	UBC2	1:40	21.2±0.1	22.1±0.5	20.7±1.0	21.5±0.3	21.4±0.5	21.6±0.2	21.8±0.7	21.5±0.1	20.8±1.2	19.8±1.3	21.0±0.5	22.1±0.9	21.2±0.1	22.3±0.3
–	ACT11	1:80	20.6±0.4	21.1±0.9	19.3±0.7	20.3±0.1	20.7±0.6	20.6±0.3	21.0±0.6	20.7±0.4	21.9±0.3	20.1±0.1	20.4±0.7	21.7±0.9	20.2±0.6	21.3±0.5
–	UBC2	1:80	23.2±0.2	24.2±0.6	22.2±1.1	23.4±0.3	23.3±0.7	23.7±0.4	23.4±0.7	23.7±0.3	24.0±0.1	22.2±0.1	22.5±0.9	24.0±1.3	21.7±0.5	23.1±0.7

[1] Cycle threshold (C_t) presented in the table was obtained by the average of all technical and biological RT-qPCR replicates ± standard error value.
[2] cDNA dilution.
[3] BR16 soybean cultivar.
[4] EMBRAPA48 soybean cultivar.

Table S11. Target and Endogenous Gene Cycle Threshold in Soybean Root Under Drought Stress – RT-qPCR[1]

Symbol	Generic Name	c.D.[2]	Time of Drought Stress (minute)													
			0		25		50		75		100		125		150	
			BR16[3]	E48[4]	BR16[3]	E48[4]	BR16[3]	E48[4]	BR16[3]	E48[4]	BR16[3]	E48[4]	BR16[3]	E48[4]	BR16[3]	E48[4]
ACO	GmACO#012	1:80	23.3±0.1	24.2±0.7	23.0±0.2	22.2±1.0	21.0±0.4	21.2±0.3	22.4±0.3	22.9±0.5	21.3±0.1	22.0±0.3	21.3±0.4	20.5±0.3	19.8±0.3	20.6±0.2
ACS	GmACS#006	1:40	30.5±0.3	30.3±0.3	27.8±0.2	26.4±0.1	27.2±0.1	27.6±0.1	28.9±0.2	30.9±0.3	32.9±0.1	30.6±0.3	29.4±0.2	30.0±0.2	29.6±0.6	31.5±0.3
CTR	GmCTR#003	1:40	29.1±0.2	28.1±0.1	30.0±0.3	29.5±0.1	30.9±0.2	30.7±0.3	31.8±0.1	30.9±0.4	33.3±0.2	31.4±0.4	31.7±0.3	31.9±0.4	32.1±0.4	32.0±0.1
ETR	GmETR#007	1:40	22.4±0.1	21.5±0.1	23.6±0.1	25.5±0.1	25.3±0.1	23.1±0.1	23.7±0.1	23.6±0.1	28.7±0.1	24.9±0.1	23.7±0.1	23.4±0.1	24.0±0.1	25.7±0.1
MAT	GmMAT#008	1:80	20.0±0.1	19.4±0.5	19.6±0.5	19.3±0.5	19.3±0.2	19.3±0.3	20.0±0.4	19.9±0.1	20.1±0.4	19.7±0.3	19.9±0.5	20.2±0.4	19.9±0.2	19.7±0.2
–	CYP2	1:40	19.1±0.1	19.0±0.6	19.6±0.4	18.9±0.3	20.1±0.1	19.3±0.5	20.5±0.7	19.6±0.3	24.2±1.4	20.0±0.8	20.2±0.3	20.3±0.4	20.8±0.1	21.4±0.5
–	ELF1A	1:40	19.2±0.2	19.1±0.7	19.7±0.3	19.1±0.4	19.9±0.1	19.9±0.6	21.1±0.7	20.3±0.2	23.9±0.9	21.1±0.6	21.3±0.2	21.4±0.4	22.3±0.2	22.5±0.4
–	CYP2	1:80	21.6±0.1	20.4±0.1	21.7±0.3	21.3±0.3	22.1±0.3	21.3±0.5	21.8±0.2	22.3±0.5	23.0±0.5	21.7±0.2	21.4±0.8	22.2±0.4	22.0±0.4	21.5±1.1
–	ELF1A	1:80	22.5±0.1	20.9±0.1	22.4±0.3	22.0±0.8	22.5±0.3	22.2±0.4	23.0±0.1	23.0±0.3	24.2±0.2	23.2±0.3	22.8±0.7	23.4±0.5	23.4±0.1	23.1±0.6

[1] Cycle threshold (C_t) presented in the table was obtained by the average of all technical and biological RT-qPCR replicates ± standard error value.
[2] cDNA dilution.
[3] BR16 soybean cultivar.
[4] EMBRAPA48 soybean cultivar.
