# Supplementary material for: Implications of ethylene biosynthesis and signaling in soybean drought stress tolerance
Source: BMC Plant Biol. 2015 Sep 3;15:213. doi: 10.1186/s12870-015-0597-z (PMC4557918; doi:10.1186/s12870-015-0597-z)
Supplement: Additional file 6: — Real Time PCR (RT-qPCR) Primers (Method S1). Gene summary and primers for Real Time PCR. (PDF 227 kb) [file 12870_2015_597_MOESM6_ESM.pdf]

Method S1. Gene Summary and Primers for Real Time PCR

Symbol	Generic Name	Locus ID	Forward Primer Sequence (5’-3’)	Reverse Primer Sequence (5’-3’)	A.S. (bp)[1]	[ ] nM[2]	Efficiency (%)
ACO	GmACO#012	Glyma14g05350	CCA ATG CGC CAT TCC ATT GTT G	TGA GGC TAC GGA CAT TCT GGT C	126	500	82.5±0.8
ACS	GmACS#006	Glyma05g36250	CTC TTA ACC TTC ATT CTT GCT AAC C	TTG CTT CTG CTT CTT TGT ATG C	187	200	86.3±0.3
CTR	GmCTR#003	Glyma10g07610	ATG ATC CCA ATT TCC TCG ATC C	GGA ATC CAT CCC GTG AAT TAG G	188	200	81.4±0.2
ETR	GmETR#007	Glyma19g40090	ATG GAT GCC TTC AAG AAG TGG	GCA CAT ATC TTC CCA CAA GAG G	188	500	85.8±0.3
MAT	GmMAT#008	Glyma15g21890	TGA TGA GAA GAC CAT TTT CCA TTT G	CAC AAT GTA GGC ACC ACT CCT A	187	500	81.7±0.3
–	ACT11[3]	Glyma18g52780	CGG TGG TTC TAT CTT GGC ATC	GTC TTT CGC TTC AAT AAC CCT A	142	500	84.6±0.5
–	CYP2[4]	Glyma12g02790	CGG GAC CAG TGT GCT TCT TCA	CCC CTC CAC TAC AAA GGC TCG	154	500	83.3±0.3
–	ELF1A[4]	Glyma05g24110	GAC CTT CTT CGT TTC TCG CA	CGA ACC TCT CAA TCA CAC GC	161	500	84.4±0.3
–	UBC2[3]	Glyma18g16160	TCC CCT CAC ACC CTT CCT C	CCA TCC CAA GGG GTG TCAT	185	500	83.6±0.3

[1] Amplicon size (base pair).
[2] Final concentration of primers used in experiments.
[3] Housekeeping genes for leaf samples normalization.
[4] Housekeeping genes for root samples normalization.
